# Investigating eukaryotic and prokaryotic diversity and functional potential in the cold and alkaline ikaite columns in Greenland

**DOI:** 10.3389/fmicb.2024.1358787

**Published:** 2024-04-09

**Authors:** Mariane Schmidt Thøgersen, Athanasios Zervas, Peter Stougaard, Lea Ellegaard-Jensen

**Affiliations:** Department of Environmental Science, Aarhus University, Roskilde, Denmark

**Keywords:** ikaite columns, Greenland, biodiversity, prokaryotes, eukaryotes, metagenome, biotechnological potential

## Abstract

The ikaite columns in the Ikka Fjord, SW Greenland, represent a permanently cold and alkaline environment known to contain a rich bacterial diversity. 16S and 18S rRNA gene amplicon and metagenomic sequencing was used to investigate the microbial diversity in the columns and for the first time, the eukaryotic and archaeal diversity in ikaite columns were analyzed. The results showed a rich prokaryotic diversity that varied across columns as well as within each column. Seven different archaeal phyla were documented in multiple locations inside the columns. The columns also contained a rich eukaryotic diversity with 27 phyla representing microalgae, protists, fungi, and small animals. Based on metagenomic sequencing, 25 high-quality MAGs were assembled and analyzed for the presence of genes involved in cycling of nitrogen, sulfur, and phosphorous as well as genes encoding carbohydrate-active enzymes (CAZymes), showing a potentially very bioactive microbial community.

## Introduction

Ikaite columns in the Ikka fjord in SW Greenland constitute a rare, permanently cold environment that is characterized by low temperatures and alkaline pH (Buchardt et al., 1997; Seaman and Buchardt, 2006). The ikaite columns are located in a marine, Greenlandic environment and the temperature inside the columns is a constant 3°C–4°C year round (Buchardt et al., 2001). This is due to the constant flow of carbonate-rich, alkaline (pH 10.4) freshwater seeping through the columns causing precipitation of ikaite (CaCO_3_ · 6H_2_O) when mixing with the surrounding seawater (Buchardt et al., 2001; Hansen et al., 2011; Stockmann et al., 2018). Recent investigations using a combination of multibeam sonar imaging and drone images have shown a total of 938 ikaite columns (0.5–20 m in height; Seaman et al., 2022). Extreme environments are often defined as having temperatures below 15°C or above 45°C, a pH outside the neutral range of pH 6–8, salinity above 10%, and/or a hydrostatic pressure greater than 200 atm (Madigan, 2000). Thus, ikaite columns with an internal pH of 10.4 and low temperatures can be regarded as dual-extreme with a normal, Greenlandic marine environment on the outside and an internal high pH, freshwater environment.

As previously reported, the ikaite columns contain a rich diversity of extremophilic prokaryotic organisms adapted to life at low temperature and high pH (Stougaard et al., 2002; Schmidt et al., 2006a,b, 2007; Schmidt et al., 2012; Glaring et al., 2015). A few studies have previously identified eukaryotic organisms living in association with the ikaite columns (Kristiansen and Kristiansen, 1999; Sørensen and Kristensen, 2000; Thorbjørn and Petersen, 2003; Trampe et al., 2017), and a single study used a sequencing-based approach to briefly look into the eukaryotic diversity inside the columns (Stougaard et al., 2002). But aside from seven identified 18S rRNA gene sequences representing different phylotypes with low levels sequence similarities to (then) known 18S rRNA genes, the eukaryotic diversity inside the ikaite columns has not been investigated.

A few other cold, freshwater environments were included in the collection of publicly available metabarcoding data on freshwater diversity in general (Debroas et al., 2017), and a few targeted studies on eukaryotic microbial diversity in freshwater environments have been carried out in, e.g., Arctic and Antarctic cryoconite holes (Cameron et al., 2012), Patagonian and Antarctic lakes (Schiaffino et al., 2016), Lake Vostoc in the Antarctic (Gura and Rogers, 2020) and ice-covered, freshwater lakes in Subarctic Russia (Zakharova et al., 2022).

Metagenomes from Polar and high-altitude environments tend to be dominated by Pseudomonadota (Proteobacteria) and/or Actinomycetota (Actinobacteria; Glaring et al., 2015; Oh et al., 2019; Rai and Bhattacharjee, 2021; Zhang et al., 2021; Singh et al., 2022), indicating that these phyla are ubiquitously present in all cold habitats. Pseudomonadota, furthermore, seems to be relatively well represented among cultured isolates from polar regions in general (Groudieva et al., 2004; Schmidt et al., 2006a; García-Echauri et al., 2011; Sathyanarayana Reddy et al., 2016; Aguayo et al., 2017; Jaarsma et al., 2023). Attempts to bring more difficult-to-culture bacterial strains into culture have for years been carried out through, e.g., application of the iChip (Nichols et al., 2010; Jaarsma et al., 2023) and similar co-culture approaches, where *in situ* nutrients and signaling molecules are made available to the microbial cultures through a diffusion membrane. Other methods for bringing hard-to-culture microorganisms into culture comprise variants of the “dilution to extinction” or other cell separation approaches to reduce competition and fast-growing species (Connon and Giovannoni, 2002; Stingl et al., 2008; Yadav et al., 2022) or through fabrication of artificial substrates with a nutrient or chemical composition as similar to the natural environment as possible (Imazaki and Kobori, 2010; Rygaard et al., 2017; Mourad et al., 2018; Wu et al., 2020).

A high-quality metagenome with DNA extracted directly from individual locations within the ikaite columns has never before been produced. Therefore, analysis of functional genes involved in microbial processes including the cycling of nitrogen, phosphorous, and sulfur and utilization of carbohydrates has never been performed. In the present study, a thorough characterization of the total microbial community inside the ikaite columns was conducted through genomic approaches. The prokaryotic and eukaryotic diversities inside three ikaite columns were compared and furthermore, the culturability of bacteria from the interior of the ikaite columns was investigated. High-quality DNA was extracted directly from the ikaite material and from the enrichment cultures, and 16S and 18S rRNA gene amplicon sequences as well as metagenomes were obtained using Illumina and Nanopore sequencing. The metagenomic data was binned to produce 25 high-quality MAGs, and these MAGs were analyzed for the presence of genes involved in cycling of nitrogen, phosphorous, and sulfur, and for expression of enzymes involved in the utilization of carbohydrates (CAZymes).

## Materials and methods

**Figure 1:**
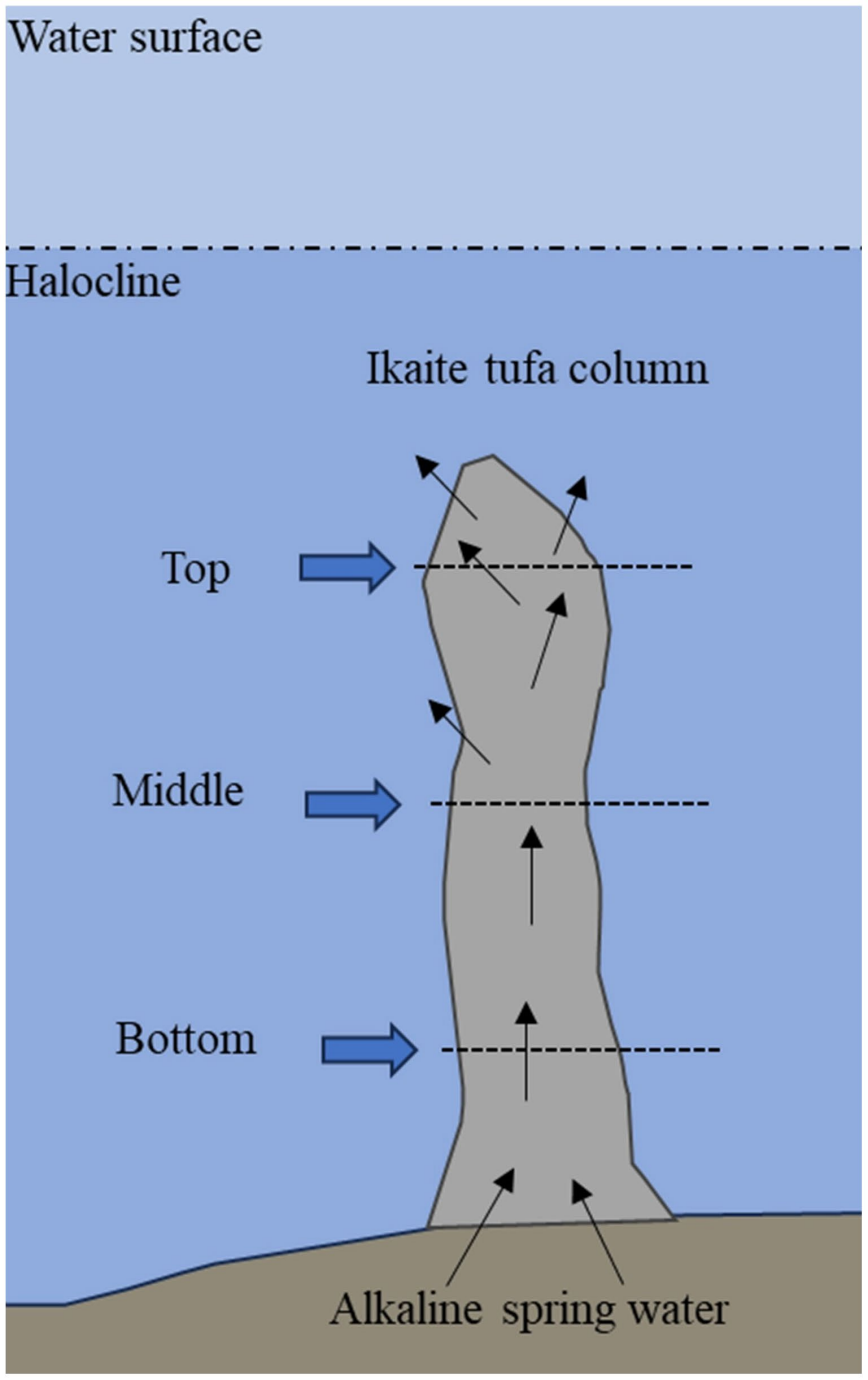
Schematic drawing of an ikaite column where sampling locations at top, middle, and bottom are indicated. Before harvesting, the top fragments were located 2.7–6.0 m below the surface, middle fragments were located 6.5–10.3 m below surface, and bottom fragments were located 10.0–13.3 m below surface. See Supplementary Table S1 for details for each column.

Three ikaite tufa columns were collected by scuba divers in the Ikka Fjord, Greenland (61°12’N, 48°00’W) in June 2019 with permission from the Greenlandic Government (License no. G19-032). The columns were between 7.5 and 10 meters in height, standing down to 13.3 meters depth at the bottom of the fjord. Each of the columns were cut into sections of 25–50 cm in length, and immediately stored at −20°C for transportation. The sections of ikaite taken from each column for this study are shown in Figure 1 and listed in Supplementary Table S1. The top sections represented the uppermost tips of each column, whereas the bottom sections were taken close to the seabed. The middle sections were taken approximately in the middle of each column—except for column 3, where the middle section was a branch off from the main column only about 3 meters from the bottom. Detailed information on sampling is available in Stockmann et al. (2022).

At the time of sampling, the temperature of the seawater surrounding the ikaite columns was between 6°C and 10°C depending on water depth. The water drained from the inside of the columns was alkaline with a pH of 10.4–10.5. In the lab, the cut-surface was removed by sawing off the outermost surface with a sterilized saw to avoid “contamination” from free-living marine organisms and from the handling of the columns during collection, packing, and storage. We thereafter drilled out ikaite material from the alkaline interior of the three individual ikaite columns. From each of the three columns, we extracted DNA from samples taken at the top, middle section, and the bottom of the columns. Since the sections of ikaite columns were visually very heterogeneous, samples were taken with five replicates distributed throughout the cut-surface to approach a fair representation of the diversity present in each section of column (Supplementary Figure S1).

Due to the physical, meta-stable state of ikaite, the drilled material resulted in a slurry of crushed ikaite mixed with alkaline ikaite water. This material was further used for inoculation of enrichment cultures and for DNA extractions (see details below).

### Culture conditions for enrichment cultures

In order to examine culturability and potential substrate-induced enrichment of bacteria from the ikaite columns, we tested a range of substrates based on R2 culture medium (per liter: 0.5 g yeast extract, 0.5 g Bacto peptone, 0.5 g Bacto casamino acids, 0.3 g sodium pyruvate, 0.3 g KH_2_PO_4_, 0.05 g MgSO_4_ · 7 H_2_O, 10 g NaCl, 0.5 g soluble starch, 0.5 g glucose) adjusted to pH 10 with Na_2_CO_3_/NaHCO_3_-buffer. Full strength R2 (“R2-gluc”), 10x diluted R2 (“R2-10-gluc”), and 100x diluted R2 (R2-100-gluc”) were used.

Variations of the 10x diluted R2 were made by substituting soluble starch and glucose with: 0.05 g/L soluble starch (“R2-10-starch”), 0.05 g/L cellulose (“R2-10-cel”), or algae polysaccharides (“R2-10-algae”: 0.05 g/L agar, 0.05 g/L fucoidan, 0.05 g/L *λ*-carrageenan, and 0.05 g/L *κ*-carrageenan). Furthermore, a substrate based on 100x diluted R2 supplemented with 2.5% olive oil and 1% Tween20 (“R2-100-lip”) was made. Finally, a substrate with water extracted directly from the inside of the ikaite columns (filtered through 0.45 μm filter) supplemented with 1 g/L casamino acids and 1 g/L sodium pyruvate was made (“IW”; Imazaki and Kobori, 2010).

To evaluate development of the enrichment cultures from a specific section of ikaite, all substrates were inoculated with ikaite material drilled from a single section (column 1, middle): As five samples (“replicas”) were drilled from the cut-surface and used for DNA extraction (see below), the remaining material (approx. 4 g) from the five replicas was pooled, suspended in 100 mL R2-100-gluc (pH 10), and vortexed thoroughly to separate the ikaite crystals. One milliliter ikaite-suspension was used to inoculate 100 mL substrate medium. Cultures were incubated aerobically at 5°C with 150 rpm in the dark, and samples were taken for DNA extraction after 195 days (T1) and 565 days (T2).

### DNA extraction and sequencing

To assess and compare the microbial diversity – prokaryotic as well as eukaryotic – inside the ikaite columns, DNA was extracted from five individual drills from each of the nine sections of ikaite and from the enrichment cultures (T1 = 195 days, T2 = 565 days) using the NucleoSpin® Soil kit (Machery-Nagel GmbH & Co., Düren, Germany) following the protocol supplied by the manufacturer, with the following modifications: For ikaite sample preparation, we used Buffer SL1 and Enhancer SX. For homogenization of the ikaite crystals and cell lysis, we performed mechanical lysis using an Omni Ruptor (Omni International, Kennesaw, GA, United States) for 2 × 45 s at 4.0 m/s. Final DNA was eluted from the spin column using 40 μL of Buffer SE, incubated 10 min at RT before final centrifugation.

The following primers were used for amplicon library building of prokaryotic 16S rRNA gene V3-V4 variable region and eukaryotic 18S rRNA gene V4-V5 hypervariable region: 16S forward 341F (5′-CCTAYGGGRBGCASCAG-3′) and reverse 806R (5′-GGACTACNNGGGTATCTAAT-3′; Hansen et al., 2012), and 18S forward 528F (5′-GCGGTAATTCCAGCTCCAA-3′) and reverse 706R (5′-AATCCRAGAATTTCACCTCT-3′; Cheung et al., 2010), all at 10 μM concentration. The amplicon library building was performed by a two-step PCR (Feld et al., 2016; Albers et al., 2018) with slight modifications. PCR reactions were conducted on a SimpliAmp Thermal Cycler (Applied Biosystems, Waltham, United States). In each reaction of the first PCR, the mix contained 12.5 μL of 2x PCRBIO Ultra Mix (PCR Biosystems), 0.5 μL of forward and reverse primer, 0.5 μL of bovine serum albumin (BSA) to a final concentration of 0.025 mg mL^−1^, 6 μL of PCR-grade water, and 5 μL of template DNA. The reaction mixture was pre-incubated at 95°C for 2 min, followed by 33 cycles of 95°C for 15 s, 55°C for 15 s, 72°C for 40 s, with a final extension at 72°C for 4 min. Samples were subsequently indexed by a second PCR. For this, amplification was performed in 28 μL reactions with 12.5 μL of 2x PCRBIO Ultra Mix (PCR Biosystems, London, United Kingdom), 2 μL of indexing primers (P7/P5), 6.5 μL of PCR-grade water, and 5 μL of PCR1 product. The cycling conditions included initial denaturation at 98°C for 1 min, followed by 13 cycles of denaturation at 98°C for 10 s, annealing at 55°C for 20 s, and extension at 72°C for 40 s, with a final extension at 72°C for 5 min. The final PCR products were purified with 15 μL magnetic beads (MagBio Genomics, Gaithersburg, United States) according to the manufacturer’s instructions and eluted in 27 μL buffer. Electrophoresis in 1% agarose gels and analysis on TapeStation 4150 with the D1000 DNA Screen-Tape (Agilent, Santa Clara, United States) were carried out to check the quality of the libraries. Finally, the DNA concentrations of the libraries were measured on a Qubit 4 fluorometer (Invitrogen, United States), and libraries were then equimolarly pooled. The pooled libraries were sequenced on an Illumina MiSeq using the 500-cycle Reagent Kit v2 yielding paired-end 2 × 250 bp reads.

To obtain a sufficient amount of high-quality DNA for metagenome sequencing, we pooled the extracted DNA from the individual samples. For shotgun metagenome sequencing, a library was constructed using the NEBNext Ultra II FS DNA library preparation kit (E6177, New England Biolabs) according to the manufacturer’s instructions and the resulting library was run on a NextSeq 500 instrument with the 300-cycles sequencing chemistry v2.5 in pair-end mode (2 x 151bp reads). From a pool of the same DNA, a library was constructed using the Ligation Sequencing Kit (SQK-LSK110, Oxford Nanopore Technologies) following the manufacturer’s instructions and sequenced on a MinION R9.4.1 flowcell controlled by MinKNOW (21.02.1). Basecalling was performed using GPU-Guppy version 6.4.6 + ae70e8f under default settings.

### Bioinformatics analysis of the microbial community

Demultiplexed reads from Illumina amplicon sequencing were analyzed using QIIME 2 v. 2022.8 (Hall and Beiko, 2018; Bolyen et al., 2019). Reads were filtered, denoised, merged, chimera checked, and dereplicated using the DADA2 (Callahan et al., 2016) with default settings. Alignment and phylogenetic trees were generated using MAFFT (Katoh and Standley, 2013) and FastTree (Price et al., 2010). Following inspection of rarefaction curves to check for saturation, the output was rarefied at 15.000 sequences for 16S and 18S of ikaite columns and 14.000 sequences for 16S of the enrichments (T0, T1, and T2). Samples with read counts lower than these values were omitted from further analysis.

Taxonomic classification was performed using qiime feature-classifier in which a pre-trained Naïve-Bayes classifier with Silva v. 138 (silva-138-99-nb-classifier) was applied (Quast et al., 2013). Features were then filtered using the qiime feature-table filter-features command to remove mitochondria, chloroplast, and eukaryotic sequences from the 16S sequences, and mammalian and bacterial sequences from the 18S sequences. Subsequently, the analyses were re-done from the alignment step as described above.

Following, taxonomic tables were constructed through the qiime taxa barplot command incorporating the metadata, and the tables were exported for further processing. The differences in the alpha diversity, based on number of observed features as amplicon sequence variants (ASVs), Faith’s phylogenetic diversity and Pielou’s evenness index were assessed by Kruskal-Wallis-Pairwise test. While the beta-diversity of the column samples, based on Bray–Curtis dissimilarity measure, was visualized by PCoA plots through EMPeror (Vázquez-Baeza et al., 2013) and analyzed using PERMANOVA (Anderson, 2001). Stacked bar charts of the communities and heatmaps of the most abundant ASVs were created in R v4.3.0^1^ using the PhyloSeq package v1.44.0 (McMurdie and Holmes, 2013).

Demultiplexed raw reads from Illumina metagenome sequencing were run through the in-house TotalRNA bioinformatics pipeline v1.1.0^2^ in order to trim the reads, perform quality control and assemble full-length rRNA genes. Information about program versions and flags are presented in detail on the Github page and on recent publications (Jaarsma et al., 2023; Scheel et al., 2023). Briefly, trimmed reads were sorted into a “Small Subunit” Pool with SortMeRNA (Kopylova et al., 2012) and the SILVA 138.1 SSU Ref NR 99 database, before being assembled in full-length rRNA contigs using MetaRib (Xue et al., 2020) and taxonomically classified with CREST4 (Lanzen et al., 2012) as described elsewhere (Anwar et al., 2019). Adapter sequences were removed from raw Nanopore reads using Porechop v0.2.4 under default settings.^3^ Trimmed Illumina and Nanopore reads were used for a hybrid metagenomic assembly using OPERA-MS v0.9.0 (Bertrand et al., 2019), under default settings. The final assembly was split into a prokaryotic and eukaryotic fraction using whokaryote v.1.1.2 (Pronk and Medema, 2022) under default settings. Contigs predicted to be of prokaryotic origin were annotated using PROKKA v1.14.6 and the predicted open reading frames (ORFs) were used as input for our in-house Jupyter Notebook Analysis pipeline v.0.0.2^4^ to identify matches to sequences present in 4 publicly available databases: CAZy (http://www.cazy.org/—version 07312019; Drula et al., 2022), NCycDB (Tu et al., 2019),^5^ SCycDB (Yu et al., 2020),^6^ and PCycDB (Zeng et al., 2022).^7^ The prokaryotic contigs were also binned with metaWRAP v.1.3.2 (Uritskiy et al., 2018) using the binning option with metaBat2 (Kang et al., 2019) and running checkM (option --run-checkm; Parks et al., 2015). Based on this approach, “High Quality” Bins are considered those with >75% completeness and <5% contamination. These HQ bins (or Metagenome-Assembled Genomes—MAGs) were also annotated using PROKKA v1.14.6 and the predicted open reading frames were used as input for the same Jupyter Notebooks as above.

## Results

The prokaryotic and eukaryotic diversity was investigated through sequence analyses of 16S and 18S rRNA gene amplifications. Three different sites in three different ikaite columns were studied and both analyses of DNA (i) extracted directly from the ikaite columns and (ii) extracted from enrichment cultures were conducted.

### Prokaryotic diversity inside the ikaite columns

**Figure 2:**
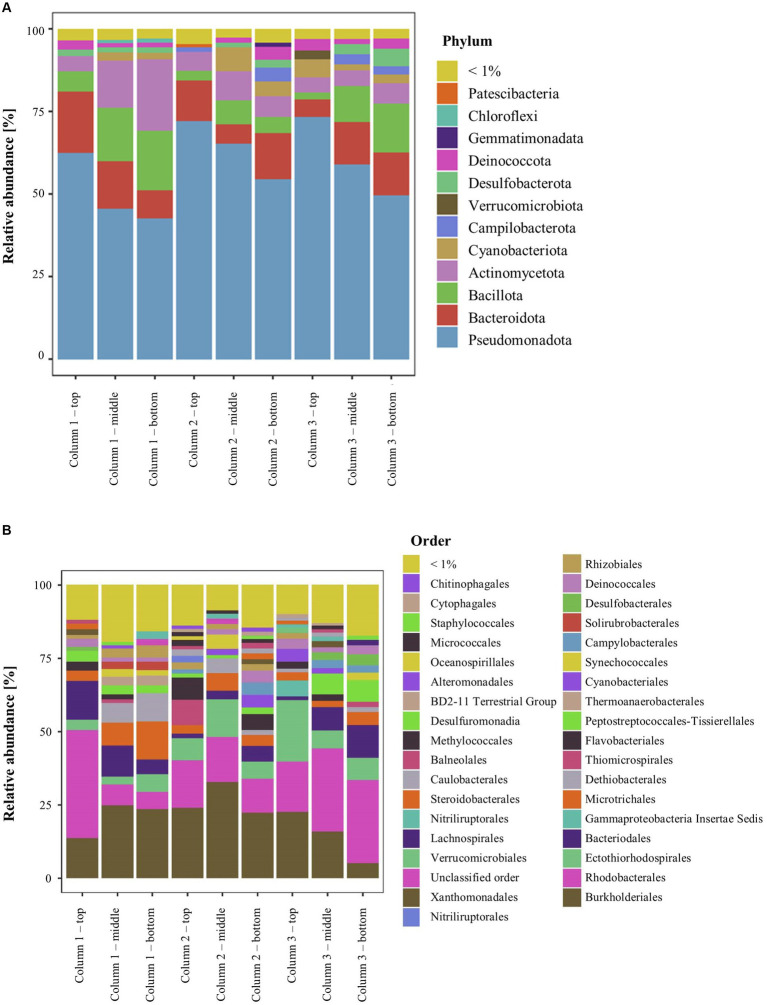
Relative abundance of 16S rRNA genes in the ikaite columns at **(A)** phylum and **(B)** order level.

In DNA extracted directly from ikaite material, 12 bacterial phyla had at least a total abundance of 1% across the whole 16S rRNA gene dataset. The relative abundance at phylum level showed that all samples were dominated by Pseudomonadota, Bacteroidota, Bacillota, and Actinomycetota (Figure 2A). The relative abundance of Pseudomonadota decreased from top to bottom samples, whereas the relative abundance of Bacillota increased. The Pseudomonadota mainly consisted of the orders Burkholderiales and Rhodobacterales, with the Rhodobacterales being highly abundant especially in the top samples with up to 37% of the relative abundance in the top fragment of column 1. Furthermore, all samples had a relatively high abundance of the orders Ectothiorhodospirales, Bacteroidales, and Microtrichales (Figure 2B). The middle and bottom samples furthermore contained a relatively high abundance (up to 13%) of bacteria belonging to the family Dethiobacteraceae, a novel family of obligately anaerobic and alkaliphilic bacteria, where the only two characterized strains are isolated from saline soda lakes (Sorokin and Merkel, 2022a,b). However, most bacterial taxa could be identified in numerous samples, supporting previous descriptions of a large prokaryotic diversity inside this cold and highly alkaline environment (Stougaard et al., 2002; Schmidt et al., 2006a; Glaring et al., 2015; Heatmap, Supplementary Figure S2).

**Figure 3:**
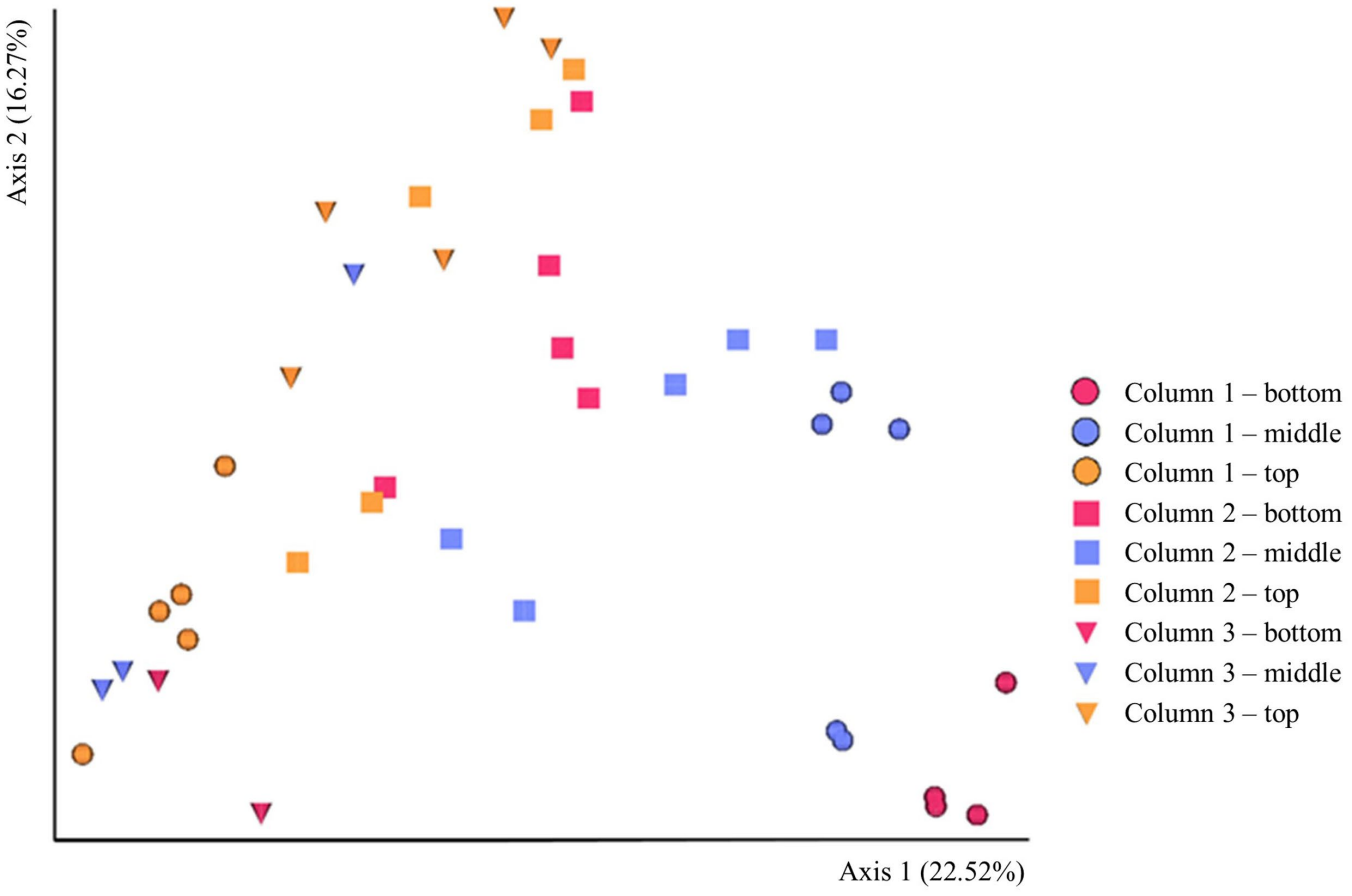
PCoA plot of the beta-diversity based 16S rRNA gene amplicons in DNA extracted directly from ikaite. Spheres: Column 1. Squares: Column 2. Triangles: Column 3. Red: Bottom samples. Blue: Middle samples. Orange: Top samples. Each mark represents an individual sample of extracted DNA.

The beta-diversity of the prokaryotic communities across the samples of DNA extracted from the ikaite columns based on 16S rRNA gene amplicons is represented by a PCoA plot (Figure 3). Each of the different fragments of ikaite had significantly different prokaryotic community composition (pairwise PERMANOVA; all *p* < 0.05), except for column 3 middle and bottom samples (*p* = 0.108; Supplementary Figure S3). On average, the samples from inside the ikaite contained between 144 (±17) and 369 (±88) ASVs (Supplementary Table S2). The number of observed ASVs were significantly different dependent on the location inside the columns (*p* = 0.006), with samples from the top of the ikaite columns containing a significantly lower number of observed ASVs than the samples collected from the bottom of the columns (*p* = 0.00098). Furthermore, the number of observed ASVs was significantly different between the three columns (*p* = 0.04; Supplementary Figure S4).

Five archaeal phyla were identified along with an unknown phylum in the low-abundance 16S rRNA gene sequences. These archaeal phyla were Crenarchaeota, Halobacterota, Nanoarchaeota, Aenigmarchaeia, and Thermoplasmatota. The most abundant archaeal orders were Nitrosopumilales (Crenarchaeota) and Woesearchaeales (Nanoarchaeota), which were found in almost all samples, and especially in the samples from column 2 (Supplementary Table S3).

### Eukaryotic diversity inside the ikaite columns

**Figure 4:**
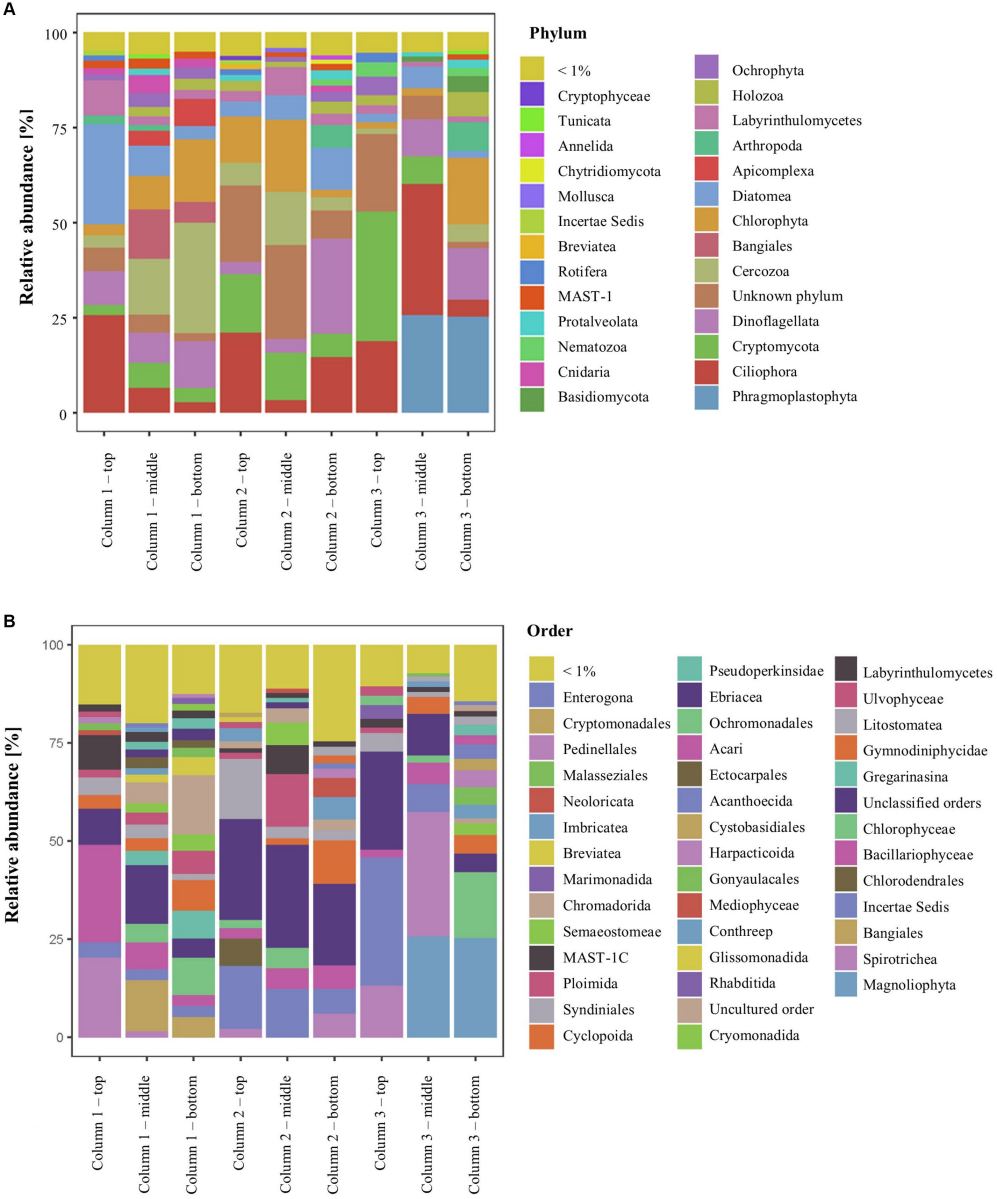
Relative abundance of 18S rRNA genes in the ikaite columns at **(A)** phylum and **(B)** order level.

The ikaite samples contained 27 eukaryotic phyla with relative abundance > 1% (Figure 4A), and among the dominating orders were Spirotrichia (ciliate protozoa), Bacillariophyceae (diatoms), and Gymnodiniphycidae (dinoflagellate; Figure 4B). In total, we identified 52 phyla plus a relatively large abundance of unclassified eukaryotes, including, e.g., MAST-stramenophiles (a complex group that includes photosynthetic algae, heterotrophic flagellates, parasites, and organisms resembling fungi; Yoon et al., 2009), and uncultured eukaryotes (Table 1).

**Table 1:** Relative abundance of eukaryotic taxa identified in the ikaite columns.

Column 1	Column 2	Column 3	Taxa	Common name/description
9.3382	11.0318	7.0444	Chlorophyta	Algae (green)
0.3308	0.2973	17.2174	Phragmoplastophyta	Algae (green)/Plants
2.7406	1.4091	2.1320	Ochrophyta	Algae (red or brown)
1.3167	2.2594	2.7097	Arthropoda	Arthropods
2.8097	0.5957	0.1742	Cnidaria	Cnidaria
0.1039	0.0719	0.1105	Ctenophora	Comb jellies
12.6123	7.1107	3.2281	Diatomea	Diatom algae
0.1179	0.1010	0.1019	Echinodermata	Echinoderms
0.0037	0.0000	0.0000	Vertebrata	Fish (g: Teleostei)
0.0109	0.1218	0.0026	Bicosoecida	Flagellates
0.0043	0.0254	0.0137	Kathablepharidae	Flagellates
0.0738	0.1352	0.0087	Platyhelminthes	Flatworms
0.0005	0.0940	0.0000	Aphelidea	Fungi
0.1010	0.0307	0.1044	Ascomycota	Fungi
0.3150	0.2272	1.8360	Basidiomycota	Fungi
0.4831	0.6073	0.2605	Chytridiomycota	Fungi
4.2090	11.3062	13.8765	Cryptomycota	Fungi
0.0057	0.0094	0.0000	Mucoromycota	Fungi
0.2584	0.2672	0.0204	Peronosporomycetes	Fungi
0.0097	0.1624	0.0131	Nucleariidae and Fonticula group	Fungi (amoeba)
0.0031	0.0000	0.0000	Stramenophiles	Heterokonts
0.0049	0.0000	0.0000	Porifera	Marine sponge
0.0766	0.4797	0.1173	Cryptophyceae	Microalgae
0.0000	0.0017	0.0000	Pav3	Microalgae
0.0035	0.0112	0.0015	Pavlovophyceae	Microalgae (haptophytes)
0.0490	0.0951	0.0374	Prymnesiophyceae	Microalgae (haptophytes)
0.1433	0.5043	0.2790	Mollusca	Molluscs
0.5571	0.9769	1.9845	Nematozoa	Nematodes
15.7453	7.7195	2.0443	Cercozoa	Protists
0.0438	0.1185	0.0227	Amoebozoa	Protists
0.0192	0.0027	0.0000	Ancyromonadida	Protists
3.7253	0.0348	0.0274	Apicomplexa	Protists
0.1739	0.5839	0.0151	Breviatea	Protists
0.0717	0.3907	0.0252	Centrohelida	Protists
0.0068	0.0038	0.0067	Discoba	Protists
0.0000	0.0295	0.0046	Euglenozoa	Protists
0.0005	0.0074	0.0347	Heterolobosea	Protists
2.1106	2.4770	3.1308	Holozoa	Protists
4.6668	4.4296	1.6960	Labyrinthulomycetes	Protists
0.0309	0.1503	0.1309	Picozoa	Protists
0.9302	1.5635	1.0559	Protalveolata	Protists
0.0017	0.0533	0.0071	Retaria	Protists
0.0180	0.0511	0.0083	SAR	Protists
11.6997	13.0555	19.1998	Ciliophora	Protists (ciliates)
9.8419	10.5645	8.2374	Dinoflagellata	Protists (dinoflagellates)
0.0019	0.0118	0.0019	Apusomonadidae	Protozoans
6.1004	0.1025	0.1309	Bangiales	Red algae
0.0000	0.0003	0.0000	Florideophycidae	Red algae
0.0285	0.0160	0.0059	Nemertea	Ribbon worms
0.5862	0.5906	1.1978	Rotifera	Rotifers
0.2231	0.3932	0.3357	Annelida	Segmented worms
0.5427	0.0596	0.4025	Tunicata	Tunicates
7.7051	19.6039	10.5654	Eukaryota	Unclassified eukaryotes

Numbers represent an average percentage per column. Darker colors highlight the most abundant taxa.

The number of ASVs ranged from 98 ± 37 to 370 ± 48 (Table S4) and was not affected by location inside the columns (*p* = 0.13; Supplementary Figure S5). Our study showed that the dominating phyla (% relative abundance per ikaite column) were representing Chlorophyta (green algae; 7.0%–11.0%) and Phragmoplastophyta (green algae/plants; 0.3–17.2), Bangiales (red algae; 0.1%–6.1%), and Diatomea (diatom algae; 3.2%–12.6%). Fungi (kingdom) were represented by eight different phyla, including Cryptomycota (4.2%–13.9%) and Basidiomycota (0.2%–1.8%) as the most dominant. The diverse group of microorganisms commonly referred to as protists contained 17 different phyla including Cercozoa (2.0%–15.7%), Ciliophora (11.7%–19.2%), Dinoflagellata (8.2%–10.6%), and Labyrinthulomycetes (1.7%–4.7%). These phyla represent mainly motile organisms, which may explain their overall distribution throughout the ikaite columns. We furthermore observed 18S rRNA genes from, e.g., cnidaria, comb jellies, flagellates, marine sponges, rotifers, and different phyla of microalgae, which showed that there is indeed a diverse eukaryotic community inside the ikaite columns but given the size of some of the living organisms, some of the DNA must evidently originate from the outside environment, perhaps due to organisms being encased during ikaite precipitation and column growth.

**Figure 5:**
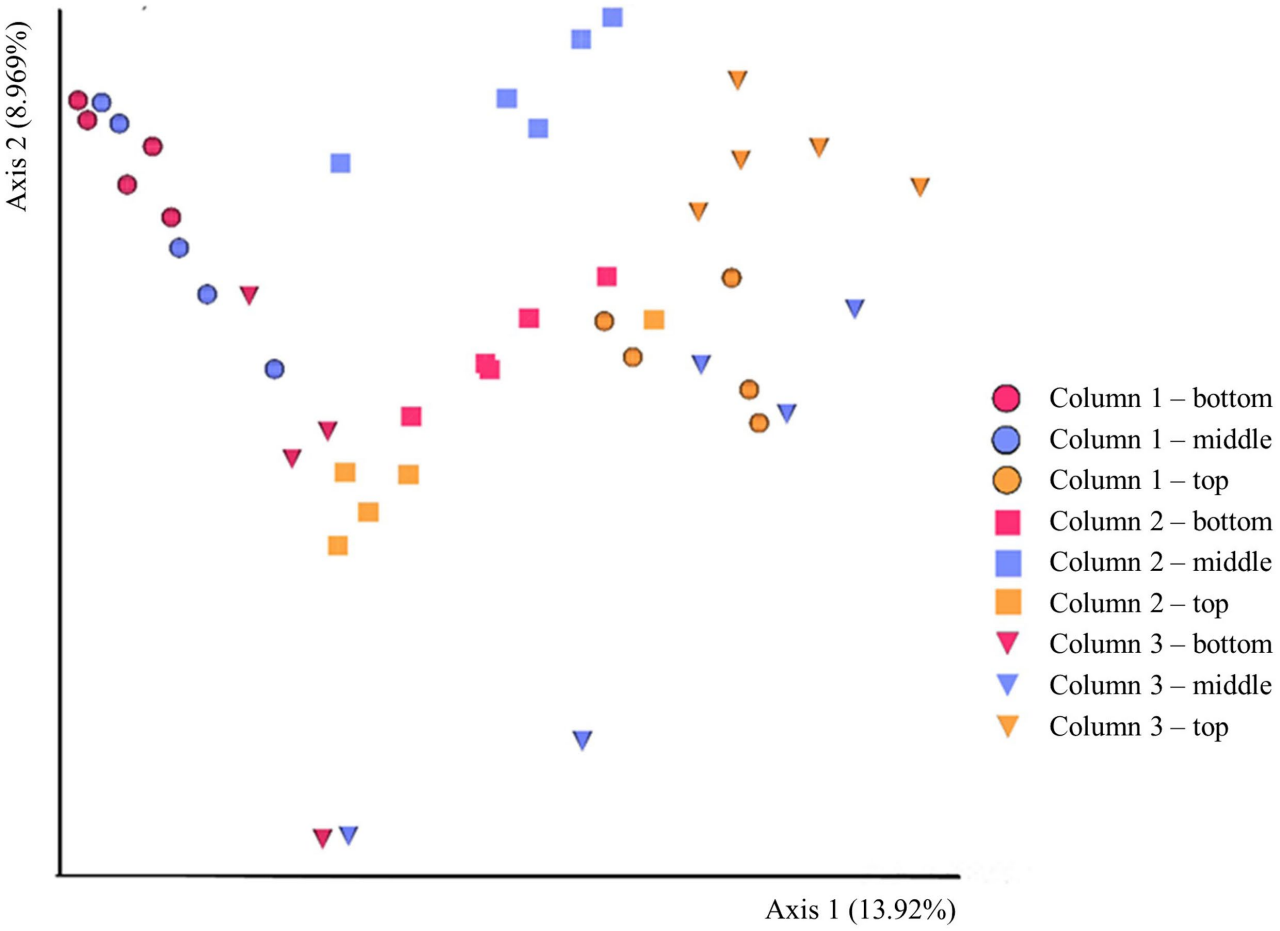
PCoA plot of the beta-diversity based 18S rRNA gene amplicons in DNA extracted directly from ikaite. Spheres: Column 1. Squares: Column 2. Triangles: Column 3. Red: Bottom samples. Blue: Middle samples. Orange: Top samples. Each mark represents an individual sample of extracted DNA.

The beta-diversity of the eukaryotic communities across the samples of DNA extracted from the ikaite columns based on 18S rRNA gene amplicons is represented by a PCoA plot (Figure 5). Each of the different locations (top, middle, bottom) in a single column had a different eukaryotic community composition (pairwise PERMANOVA; all *p* < 0.05), except for middle and bottom samples in column 1 (*p* = 0.112) and column 3 (*p* = 0.131). The alpha-diversity regarding the richness shows that there is a significant difference in number of ASVs between the columns (*p* = 0.018; Supplementary Figure S6), with column 3 having a significantly lower number of ASVs than columns 1 and 2, the distribution of the most abundant taxa in each sample is illustrated in the heatmap in Supplementary Figure S7.

### Microbial diversity of ikaite enrichment cultures

The culturing approach only enriched for prokaryotic organisms as evident from the PCR amplification. Amplification with 16S rRNA gene-specific primers resulted in good amplification, whereas 18S rRNA gene-specific primers did not result in visible bands on the agarose gel and was therefore excluded from further analysis. We sampled the cultures for DNA extraction after 195 days (T1) and again after 565 days (T2). After 195 days of incubation, the number of ASVs were substantively reduced in all substrates (Supplementary Table S5), and since neither the beta-diversity nor the community composition hardly changed from T1 to T2 (Supplementary Figures S8–S10), we chose in the following to focus on the diversity in the T1 samples as compared to the diversity inside the ikaite column (T0).

Overall, the ikaite sample (T0, *n* = 5), which represents DNA extracted directly from the ikaite columns before inoculation (column 1, middle), had a higher prokaryotic diversity than the enriched samples. A heatmap of the 50 most abundant taxa across the dataset of T0 and T1 samples with different substrates can be seen in Supplementary Figure S11.

**Figure 6:**
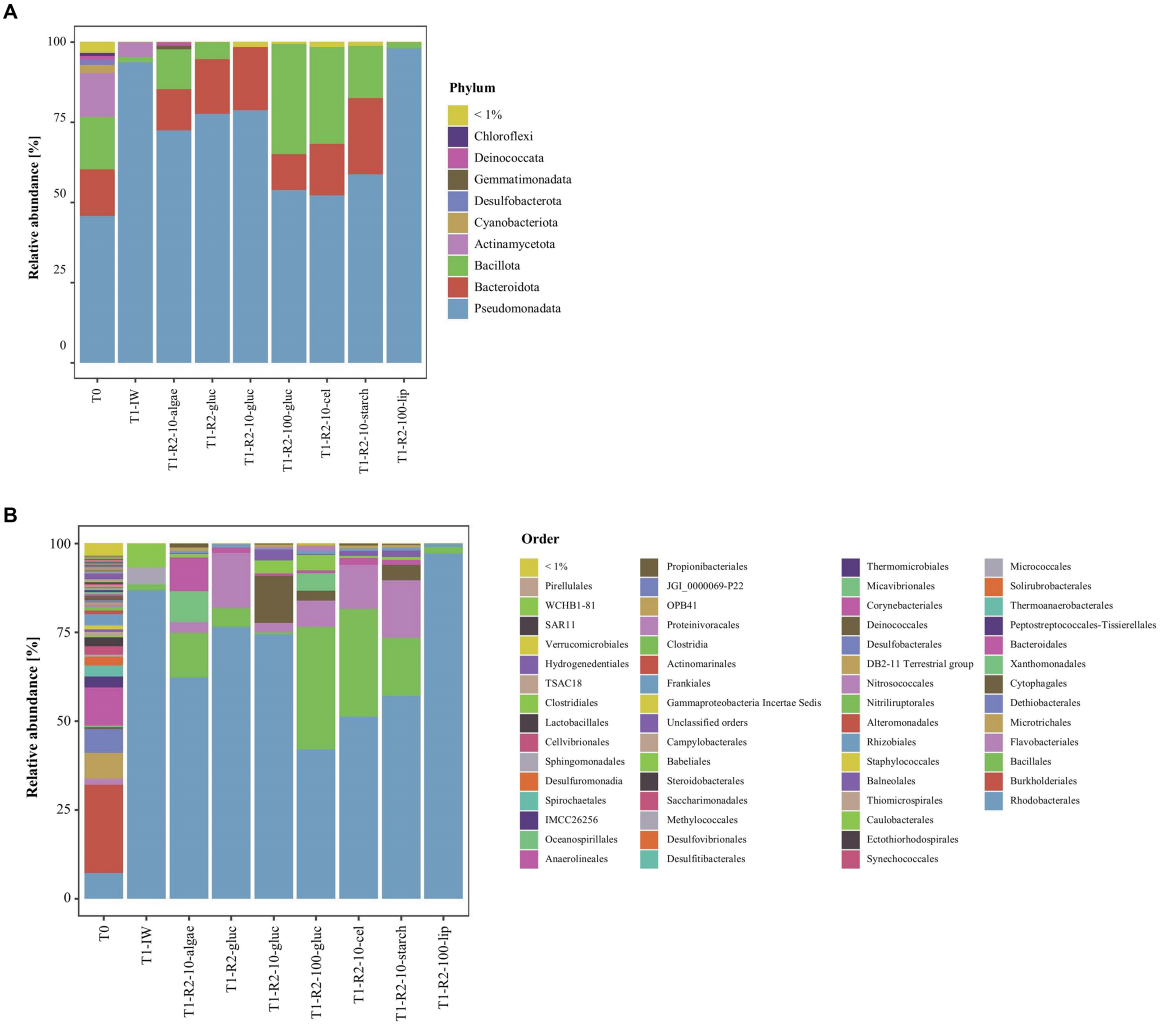
Relative abundance of 16S rRNA genes in the enrichment cultures at **(A)** phylum and **(B)** order level. DNA was sampled on the day of inoculation (T0) and after incubation in a range of substrates for 195 days (T1).

Community composition analysis revealed six phyla with relative abundances >1% were identified in the enrichment cultures (T1): Pseudomonadota, Bacteroidota, Bacillota, Actinomycetota, Gemmatimonadota, and Deinococcota (Figure 6A). Cyanobacteriota and Chloroflexi were only identified in the ikaite samples (T0). Pseudomonadota, Bacteroidota, and Bacillota by far dominated all cultures. In the R2-based substrates containing glucose, cellulose, starch, and lipid, the Pseudomonadota were dominated by an unclassified genus belonging to the order Rhodobacterales (Figure 6B). The T1-IW-samples, which were incubated in alkaline water extracted directly from the ikaite columns, therefore simulating the natural environment as closely as possible, were dominated by bacteria belonging to the genus Yoonia-Loktanella, also belonging to the Rhodobacterales. Bacteroidota was present in all samples with the exception for the IW-substrate and the R2_100_lip, indicating that these bacteria require other nutrients than provided in the IW-substrate, but were also not able to utilize lipids as a carbon source.

**Figure 7:**
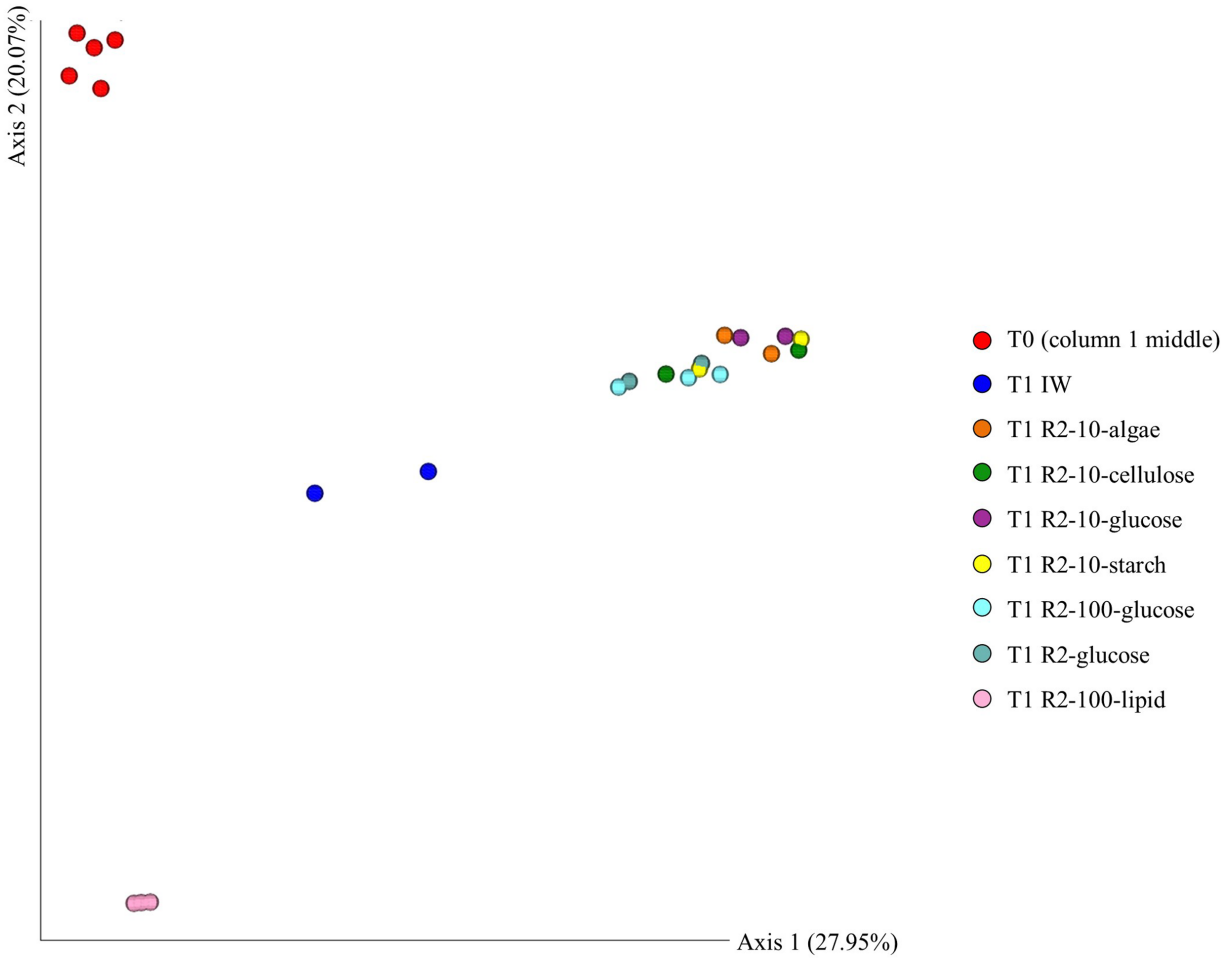
PCoA plot of the beta-diversity in ikaite enrichment cultures based on 16S rRNA gene amplicon sequencing. DNA was sampled on the day of inoculation (T0) and after incubation for 195 days (T1). Yellow spheres: Ikaite (T0). Dark blue spheres: ikaite water-based substrate (IW, T1). Dark red spheres: Oil-based substrate (R2-100-Lip, T1). Remaining spheres: R2-based substrates (T1).

The beta-diversity of the enrichments cultures as illustrated in the PCoA plot in Figure 7 showed that the prokaryotic communities identified in the T0-samples, the IW- and the lipid-based substrates are distinct from the remaining enrichments.

Figure 6B furthermore showed that the DNA extracted directly from the ikaite (T0) contained a relatively large proportion of Burkholderiales and Microtrichales, which were only seen in very low abundance in the enrichments, and Dethiobacterales, which were not found in any of the enrichments. Actinomycetota were only, with a very few exceptions and in very low abundance, observed in the T0-samples, indicating that these microorganisms are difficult to grow under our culturing conditions.

The lowest richness was observed in the enrichment cultures containing olive oil (R2-100-lip) with 7 ± 3 ASVs (Supplementary Table S5). These cultures contained only two phyla: Pseudomonadota and Bacillota. The Pseudomonadota were represented by the two orders Rhodobacterales and Rhizobiales. A single genotype within the Rhodobacterales, which could not be affiliated to any genus, made up 97.3% ± 1.4% of the relative abundance. Within the Bacillota, a bacterium within the genus *Salinarimonas* of the order Rhizobiales was identified to species level as *Alkalilactibacillus ikkense*, a cold-active, alkaliphilic bacterium previously isolated and characterized from the ikaite columns (Schmidt et al., 2012). This genotype was observed in all enrichment cultures, despite only being identified in very low abundance in amplicons from DNA isolated directly from the ikaite material (0.0038% relative abundance), indicating that it is an easily cultured bacterium when using R2-based substrates.

The highest number of ASVs was found in 100x diluted R2 supplemented with glucose (37 ± 9), in the 10x diluted R2 supplemented with soluble starch (33 ± 7), and in the 10x diluted R2 supplemented with glucose (32 ± 2; Supplementary Table S3). R2-100-gluc and R2-10-starch were, besides Rhodobacterales, dominated by Bacillales and Flavobacterales, despite these only being found in low abundances in the ikaite (T0). The substrate supplemented with algae polysaccharides (R2-10-algae) contained a relatively large proportion of Bacillales, Flavoracterales, Xanthomonadales, and Bacteroidales (Figure 6B).

### Microbial diversity in the ikaite metagenome

The diversity in the ikaite metagenome was evaluated based on assembled, full-length small subunit (SSU) rRNA genes. From 18,431,301 trimmed pair-end Illumina reads (5,56 gigabases), 71,472 reads (0.38%) were sorted out and 91 contigs were assembled. These 91 contigs comprised 77 prokaryotic and 14 eukaryotic rRNA gene sequences. In this context, calculating relative abundances does not provide biological meaning. The eukaryotic sequences belonged to two algal genera, namely *Bangia* (Rhodophyta) and *Acrosiphonia* (Chlorophyta), and 12 SAR taxa distributed between Ochrophyta (2), Dinoflagellata (4), Ciliophora (1) and Cercozoa (5), potentially representing novel organisms, except for *Pseudopedinella elastica* (Pedinellales, Ochrophyta), *Alexandirum* (Gonyaulacales, Dinoflagellata) and *Cryothecomonas* (Cryomonadida, Cercozoa). Compared to the amplicon sequencing approach, the ikaite metagenome showed significantly lower diversity. *Bangia* was found in 13 of the ikaite columns samples, while *Acrosiphonia* only in one. Eukaryotic taxa with resolved phylogeny down to the genus level represent organisms that have been found in cold waters, while *P. elastica* has been found in brackish waters as well. Compared to the 18S rRNA *Alexandrium* was found in 24 samples, *Cryothecomonas* in 33, and *Pseudopedinella* in none, but “Uncultured Pedinellales” were present in 25 samples, though in small relative abundances.

**Figure 8:**
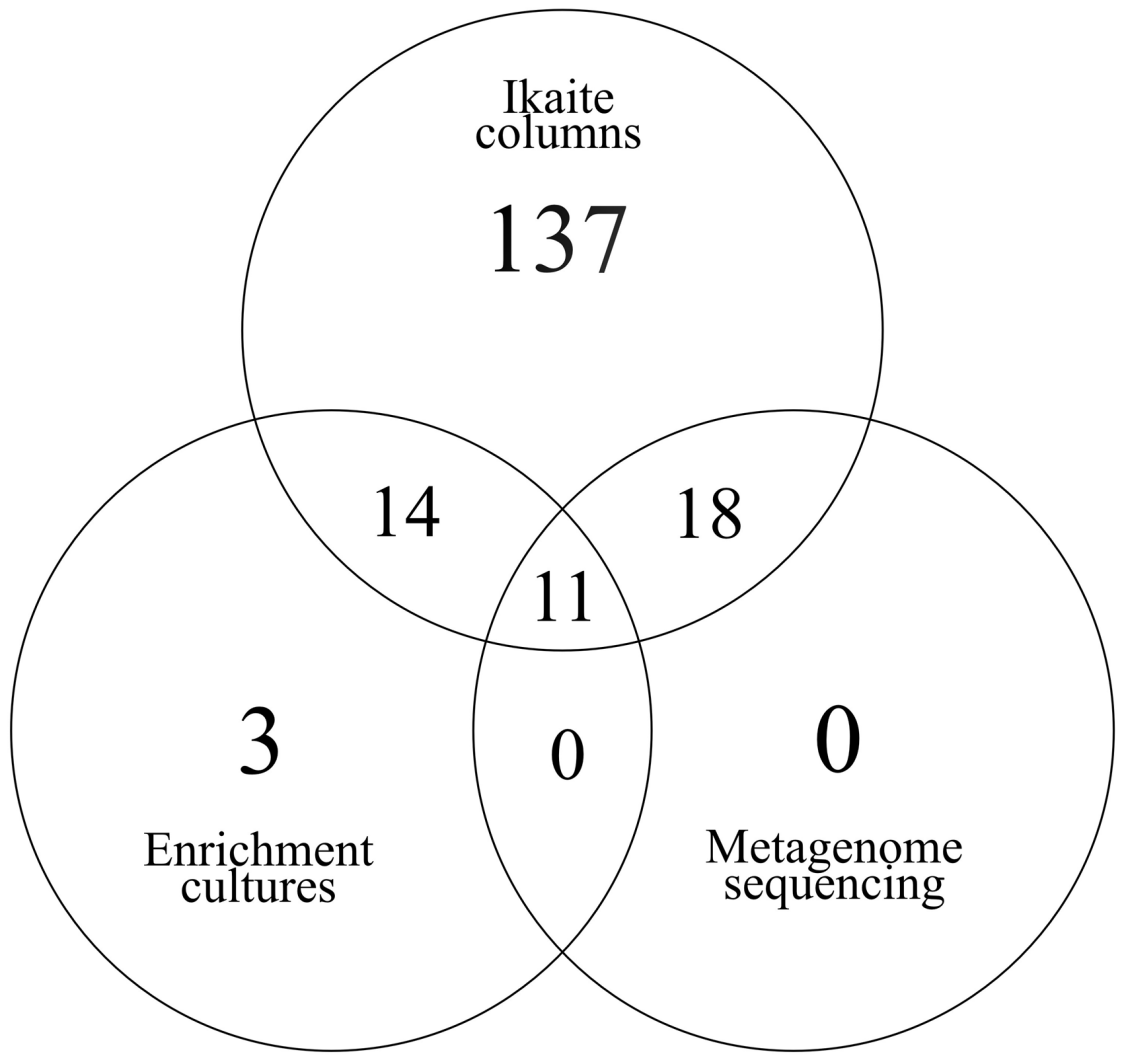
Venn diagram of 16S rRNA genes from the enrichment cultures (amplicons) and from DNA isolated directly from the ikaite material (amplicons and metagenome). The ikaite amplicons contained the highest number of unique sequences (137) and shared 25 sequences with the enrichment cultures and 29 sequences with the metagenome. The metagenome did not contain any unique sequences but shared all its sequences with the amplicons from the ikaite material, and 11 of its sequences with the enrichment cultures. The enrichments contained three unique sequences: one affiliated to Acidobacteriales (Actinomycetota), one affiliated to Ktedonobacterales (Chloroflexi), and one affiliated to Acidaminococcales (Bacillota).

The assembled prokaryotic 16S rRNA genes at the order-level were distributed between the different phyla as: Actinomycetota (5), Bacillota (8), Bacteroidota (3), Chloroflexi (2), Cyanobacteriota (2), Deinococcota (1), Desulfobacterota (1), Planctomycetota (1), and Pseudomonadota (8). These taxa represent 31/180 (17%) of the taxa (at order-level) found in the 45 amplicon sequencing datasets from the ikaite columns, while they account for 11/28 (39%) of the taxa found in the enrichment cultures. At this level, there were no taxa found in common between the metagenome and the enrichment cultures that were not also present in the ikaite amplicons (Figure 8). On the other hand, 18 prokaryotic taxa that were found in common between the metagenome and the ikaite columns were not present in the enrichment cultures and 14 taxa were present in the enrichment cultures and ikaite columns but not in the metagenome.

### Ikaite MAGs

Reads in the ikaite metagenome were binned to produce metagenome-assembled genomes (MAGs). This produced a total of 69 MAGs out of which 25 MAGs were considered by the program as high-quality (Supplementary Tables S6, S7). The 25 high-quality (HQ) MAGs were phylogenetically identified based on 16S rRNA, *gyr*B, *rpo*A, and *rpo*D gene sequences (Das et al., 2014), and belonged to the bacterial phyla Pseudomonadota (9), Bacteroidota (4), Actinomycetota (3), Bacillota (3), Cyanobacteriota (2), Chloroflexota (1), Nitrospirota (1), Thermodesulfobacteriota (1), and one inconclusive. The higher representation of Pseudomonadota, Bacteroidota, Bacillota, and Actinomycetota in the MAGs corresponds well with the 16S amplicon analyses, which were also dominated by sequences affiliated to these four phyla.

### Cycling of N, P, and S in the ikaite columns

**Figure 9:**
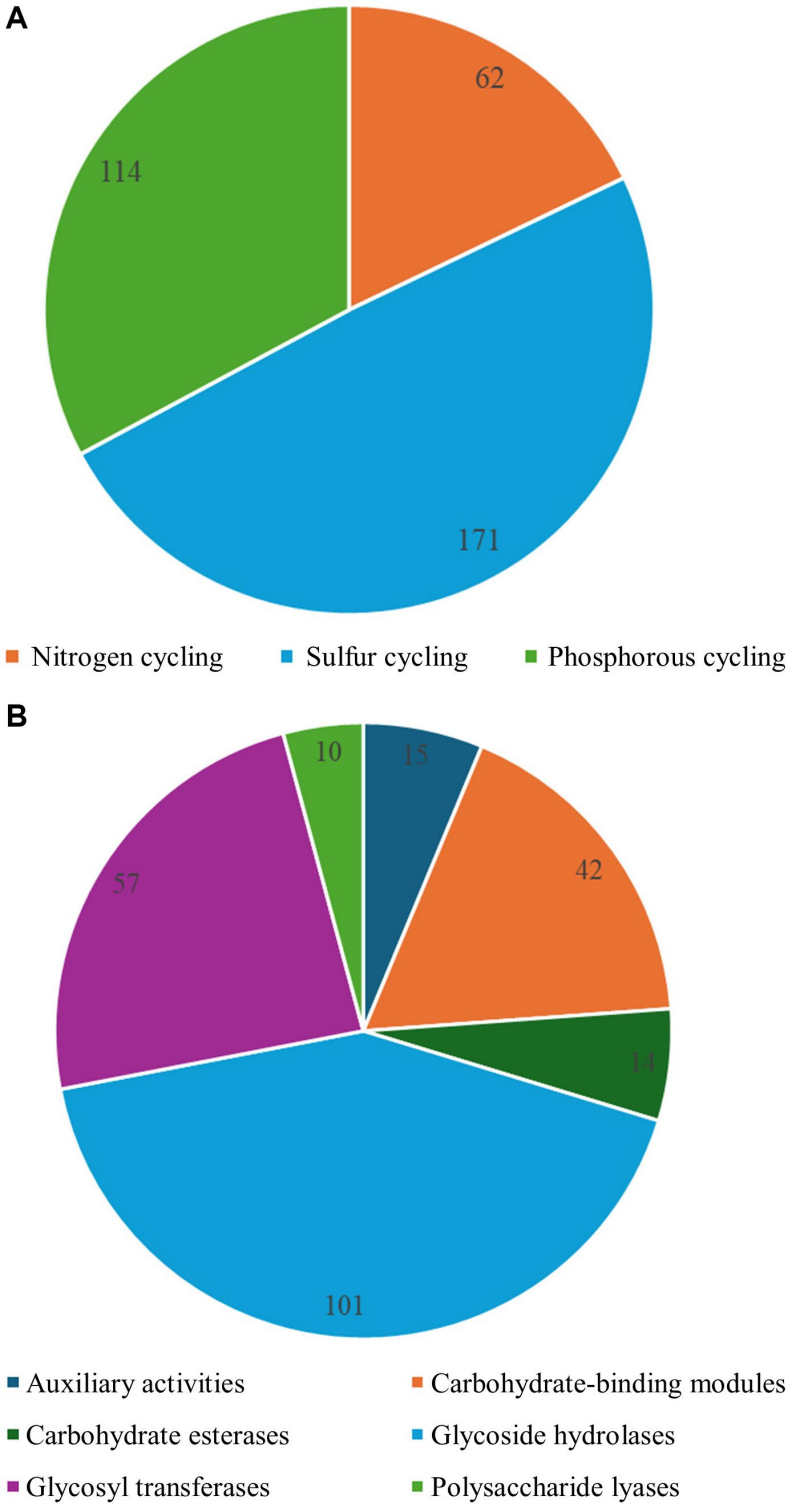
Distribution of **(A)** genes related to cycling of nitrogen, phosphorous and sulfur and **(B)** genes encoding carbohydrate-active enzymes (CAZymes) identified in the 25 high-quality MAGs from the metagenomic sequencing of DNA from the ikaite columns. A detailed table of the genes identified can be found in Supplementary Tables S8–S12.

As it was beyond the scope of the paper, we did not aim at analyzing metabolic pathways, however by looking at the presence of genes and their known activities, we were able to identify at least some of the processes potentially taking place inside the ikaite columns based on genes present in the assembled HQ MAGs. In the high-quality MAGs, we identified multiple genes potentially involved in the cycling of nitrogen (N) and phosphorous (P), and sulfur (S) (Figure 9A; Supplementary Tables S8–S11). All MAGs contained genes for assimilatory and dissimilatory nitrate reduction, denitrification, nitrogen fixation, or organic degradation and synthesis (Supplementary Table S10). The MAGS with the highest number of genes related to nitrogen cycling were MAGs 3, 16, 24, 33, and 37 affiliated to with the phylum Pseudomonadota and MAG 71 affiliated to Nitrospirota. Genes involved in denitrification included *nap*A/B/C, *nar*G/H/I/J/V/Y/Z *nir*K/S, *nor*B/C, and *nos*Z (Stewart et al., 2002; Chen and Strous, 2013; Sparacino-Watkins et al., 2014), and in nitrogen fixation *nif*D/H/K/W (Dos Santos et al., 2012; Pi et al., 2022). Furthermore, we identified genes involved in degradation of organic nitrogen (*asn*B, *gln*A, *gls*A, and *ure*A/B/C; Cruz-Ramos et al., 1997; Peng et al., 2024), assimilatory and dissimilatory nitrate reduction (*nar*A/CCG/H/I/J/V/Y/Z, *nas*A/B, *nir*A/B/D, *nrf*A/C/D, and NR), nitrification (*hao* and *nxr*B), and anammox (*hzs*B; Sorokin et al., 2009; Stackebrandt and Otten, 2014; D'Angelo et al., 2023).

The MAGS furthermore contained genes for phosphate transport (*phn*A/C/D/E/F/G, *pst*A/B/C/D, and *ugp*A/B/C/E; Voss et al., 2013; Adhikari et al., 2017), in mineralization of organic P (alkaline phosphatases *pho*A, *pho*D, and *pho*X), in mineralization of phosphonates (C-P lyases *phn*F/G/H/I/J/K/L/M; Voss et al., 2013; Seweryn et al., 2015), in solubilization of inorganic P (*gcd*, *ppa*, *ppx*; Wu et al., 2022), and as P-starvation response regulators (*pho*B/H/R/U; diCenzo et al., 2017; Siles et al., 2022; Bruna et al., 2023; Moreau, 2023).

We identified five MAGs and numerous 16S rRNA amplicons as potentially being affiliated to, e.g., *Thiobacillus*, *Thioalkalivibrio*, Desulfobacterales, Ectothiorhodospirales, Dethiobacterales, and Thiomicrospirales, which are anaerobic bacteria with an active role in sulfur cycling. In the HQ MAGs, we identified multiple genes involved in sulfur oxidation (*sox*A/B/C/D/L/X/Y/Z; Grabarczyk and Berks, 2017), genes involved in assimilatory sulfate reduction (*sat*, *cys*N-D, *cys*C/H/J/I, *sir*, *hdr*A/B/C/D) and dissimilatory sulfate reduction and oxidation (*sat*, *apr*A/B, *dsr*, *psr*A/B, *fcc*A/B, *soe*A/B/C, *qmo*A/B/C, *sor*; Guo et al., 2016; Nagar et al., 2022; Tanabe and Dahl, 2022), amino acid metabolism (*cys*E/K/M, *met*A/B/X/Z; Ono et al., 2017; Gai et al., 2021), cycling of organic sulfur compounds like DMSO and DMSP (*ddd*L, *dmd*A/B, *dms*A/B/C; Lidbury et al., 2016; Xiong et al., 2016; Tebbe et al., 2023), and methane metabolisms (*dmo*A, *mdd*A, *mts*A/B; Nagar et al., 2022; Shi et al., 2023). We furthermore identified gene clusters for sulfonate utilization (*tau*A/B/C/D and *ssu*A/B/C/D/E; van der Ploeg et al., 2001; Koch et al., 2005), and modification of bacterial tRNAs (*tus*A/B/C/D/E; Ikeuchi et al., 2006).

### CAZy genes in MAGs from the ikaite metagenome

The 25 high-quality MAGs were mined for carbohydrate-active enzyme (CAZy) genes. Genes were identified belonging to carbohydrate-binding module (CBM) families, carboxyl esterase (CE) families, glycosyl hydrolase (GH) enzyme families, glycosyl transferase (GT) families, and polysaccharide lyase (PL) families, excluding the genes not yet assigned to a family and genes encoding auxiliary activities (Figure 9B; Supplementary Table S12). Due to our current work on hydrolytic enzymes, we chose to focus our further analyses on the GH family enzymes identified in the high-quality MAGs. These MAGs contained genes from between 24 and 60 GH families. The MAGs with the highest number of different GH family enzyme genes were affiliated to Bacteroidota (MAG26 and MAG36), Cyanobacteriota (MAG29), and Actinomycetota (MAG1 and MAG65). GH families 2, 3, 6, 19, 23, and 37 were found in all 25 MAGs, whereas more exclusive families with only one or few known activities like, i.e., GH 50, 140, 141, and 165 were only identified in MAG26 affiliated to Bacteroidota. This MAG had genes belonging to 60 different GH families, potentially indicating a great potential for enzymatic hydrolysis of a range of complex carbohydrates. Unfortunately, it was not possible to identify the MAG below phylum-level based on the phylogenetic markers, however this would be a great candidate for *in vitro* gene expression and analyses of enzymatic activities.

## Discussion

### Biodiversity inside the ikaite columns

In this study, we show that the cold and alkaline ikaite columns from the Ikka Fjord in SW Greenland contain a great diversity of prokaryotic as well as eukaryotic microorganisms.

Despite previous efforts to apply a sequence-based approach to describe the total microbial diversity of the ikaite columns (Stougaard et al., 2002; Schmidt et al., 2006a; Glaring et al., 2015), results have been rather restricted due to either available sequencing technologies or lack of high-quality DNA for sequencing, and only a single study has previously used a 16S rRNA gene amplicon-based approach to describe the prokaryotic diversity (Glaring et al., 2015). In that study, the authors performed pyrosequencing on 16S rRNA genes in order to further describe the bacterial diversity of the columns. However, this was not separated into individual columns, but rather a pool of samples from the interior of multiple ikaite columns and related to the bacterial diversity on the surface of the column, the seawater surrounding the columns, and sediment from the Ikka Fjord. As this study showed that the interior of the ikaite columns, the surface of the ikaite columns, the seawater, and sediments around the ikaite columns contained very distinct prokaryotic populations (Glaring et al., 2015), we chose to focus on comparison of the microbial diversity between the interior of three individual ikaite columns. Another previous study attempted to compare the microbiomes between different ikaite columns (Schmidt et al., 2006a). This was carried out in 2006 using terminal-restriction fragment length polymorphism (T-RFLP) analysis of 16S rRNA gene fragments amplified from DNA isolated from three individual ikaite columns. Despite the, by today’s standards, low resolution of this approach, it showed that the three sampled ikaite columns contained phylogenetically distinct microbial populations. Based on a 16S rRNA gene clone library, it was possible to link the T-RFLP to a few bacterial classes, dominated by Pseudomonadota.

In the current study, we compared the microbial biodiversity inside three ikaite columns as well as comparing the indigenous column biodiversity to that of *in vitro* enrichment cultures from the same column. No eukaryotes were identified in the enrichments, why we in the following make the comparisons between the prokaryotic communities alone. As shown in the VENN diagram, the metagenome did not contain any unique sequences, whereas the enrichment cultures had three unique sequences not identified in the ikaite amplicons nor in the enrichment culture amplicons. The three unique sequences belonged to three different phyla and were found in substrates based on 100x diluted R2 medium, supplemented with either glucose or olive oil, and only in very low numbers (0.015%–0.03% relative abundance). Despite the low number of unique sequences, this indicates that low-nutrient substrates are indeed applicable when attempting to increase culturability of novel bacterial taxa (Connon and Giovannoni, 2002; Imazaki and Kobori, 2010).

Amplicon sequencing of 16S rRNA genes in DNA isolated directly from the ikaite columns showed that the dominating phyla were Pseudomonadota (Gamma and Alpha), Bacteroidota, Bacillota, Actinomycetota, and Cyanobacteriota. Especially Pseudomonadota and Bacteroidota are often dominating in cold environments like sea ice (de Sousa et al., 2019; Strong et al., 2021; Winder et al., 2023), permafrost (Frey et al., 2016; Hu et al., 2016), and freshwater ecosystems (Cameron et al., 2012; Cavaco et al., 2019). Cyanobacteriota are abundant in sea ice, but can also be found in, e.g., permafrost samples (Frey et al., 2016; Hu et al., 2016) and cryoconite holes, where they are the main primary producers (Anesio and Laybourn-Parry, 2012). A similar role may be relevant in the ikaite columns, where cyanobacterial phycobiliproteins have been shown to be located at certain distances from the surface of the columns corresponding to the light penetration (Trampe et al., 2016, 2017).

Comparing the prokaryotic diversity in the enrichment culture amplicons to the ikaite column amplicons and MAGs, the amplicons contained a relatively large proportion of Burkholderiales and Microtrichales, only seen in very low abundance in the enrichments, and of Dethiobacterales, which were not found in the enrichments at all. Dethiobacterales is an order consisting only of a single family, Dethiobacteraceae, and the genus *Dethiobacter*, with only a single characterized species, *D. alkaliphilus*, which is a haloalkaliphilic, anaerobic sulfate reducer (Zavarzina et al., 2018; Sorokin and Merkel, 2022a,b). Since the species described is halotolerant, we assume that we have a new strain within the genus or family, due to the low salinity (0.9%) in the ikaite columns (Buchardt et al., 1997). But since Dethiobacterales is not identified in our enrichment cultures, which were incubated under aerobic conditions, we cannot conform this theory from the current experimental data. Several other orders containing anaerobic prokaryotes were identified from the ikaite amplicons and the MAGs: Ectothiorhodospirales, Thiobacillus, and Thiomicrospirales, which are part of the “purple photosynthetic” bacterial group and are capable of oxidizing inorganic sulfur compounds as energy sources, with Thiobacillus also able to use ferrous iron as an energy source (Kuenen and Tuovinen, 1981; Lane et al., 1985; Oren, 2014). Thioalkalivibrio is a group of haloalkaliphilic sulfur-oxidizing bacteria that includes species able to oxidize thiocyanate, a toxic compound found in industrial waste streams (Berben et al., 2017). Anaerolineales is an order of obligate anaerobes found in marine sediments or sludge (Yamada et al., 2006). Thermoanaerobacterales is an order belonging to the phylum Bacillota, and contain thermophilic, fermentative anaerobes capable of hydrolyzing a range of plant polysaccharides (Bing et al., 2023). Desulfovibrionales contains species that besides anaerobic dissimilatory sulfate-reduction, are capable of mercury methylation (Gilmour et al., 2011). And Clostridiales (re-classified as Eubacteriales) is a diverse order within the Clostridia of obligate anaerobic, spore-forming bacteria (Setlow and Doona, 2023). Only Thermoanaerobacterales and Clostridiales have previously been identified in the ikaite columns (Glaring et al., 2015). We did expect anaerobic sulfate reducers to be present in the ikaite columns due to a distinct smell of sulfur in freshly sampled ikaite columns, and the relative high abundance of particularly sulfate reducers show that this must be an important metabolic process of the bacteria inside the ikaite columns. Since our enrichments were incubated under aerobic conditions, it corresponds well that they did not appear in the enrichment amplicon sequences. We therefore suggest that future enrichment studies from ikaite columns should include anaerobic culture conditions to potentially promote these taxa.

Sequences affiliated to Deinococcales (Deinococcota) were identified in the enrichments (all 10x and 100x diluted R2, except for R2_100_lip) as well as in the ikaite amplicons and the metagenome. They could be identified as belonging to the genus *Truepera*, which to date only contains one classified species, *Truepera radiovictrix*. Further three strains have been isolated (Albuquerque et al., 2005) and all strains are characterized by optimal growth at around 50°C and pH 7.5–9.5 under aerobic conditions and being extremely tolerant of ionizing radiation. So, since we find bacterial sequences assumably affiliated with the genus *Truepera* inside the ikaite columns, these are expected to be able to grow at lower temperatures but in a similar pH-range as the type-strain, or potentially belong to another, but closely related, genus with different growth characteristics.

The most abundant archaeal phyla were the orders Nitrosopumilales (Crenarchaeota) and Woesearchaeales (Nanoarchaeota). Nitrosopumilales contains only a few cultured strains, mainly represented by marine, aerobic archaea (Qin et al., 2017; Bayer et al., 2019; Nakagawa et al., 2021), whereas Woesearchaeales has no officially classified members. They do seem, however, to be widely distributed, mainly in anoxic environments (Liu et al., 2018). But since the 16S sequences of the phylogenetically closest relatives of the ikaite archaea belonged to uncultured species and candidate genera, we are not able to speculate further in the functions of the archaea from ikaite columns. The presence of five archaeal phyla in the ikaite columns, despite being in relative low abundance, underscores the uniqueness of this ecosystem and raises questions about the functional roles of archaea under such extreme conditions. The large diversity of microorganisms inside the ikaite columns furthermore underlines the adaptability of a microbial community to extreme conditions.

Statistical tests of the prokaryotic diversity inside the ikaite columns demonstrated a microbial community composition dependent on both a specific ikaite column and location inside the column (Figure 4). The reason that column 3 middle and bottom samples are more identical than the remaining samples could be explained by the formation of the middle section, which was a branch off from the main column formed by more recent precipitation and perhaps therefore was more recently colonized by microbes from the bottom section due to the constant flow of water from the seabed. The clustering pattern could suggest that the microbial communities within each column exhibit some level of specialization based on their location from top to bottom within the column. This could be due to variations in environmental factors, such as availability of light, oxygen, and nutrients (Trampe, 2016; Trampe et al., 2016, 2017). The latter could be explained by our findings of anaerobic bacteria and archaea primarily in the middle and bottom sections of the columns. Also, as the relative abundance of Pseudomonadota decreased from top to bottom while the relative abundance of Bacillota increased, indicated a potential adaptation of different bacterial groups to different conditions inside the columns. Furthermore, it has previously been shown that ikaite crystals are covered in exopolymeric material (Trampe et al., 2017), which is likely contributing to a degree of local organization of some microorganisms inside the columns, whereas others may be free-living in the alkaline spring water resulting in a more universal distribution.

The ikaite columns are formed by precipitation of calcium carbonate due to mixing of seawater and alkaline spring water (Buchardt et al., 2001). The columns collected in 2019 consisted of not only ikaite, but to some extent also monohydrocalcite and aragonite, which are less hydrated carbonate minerals than ikaite (Stockmann et al., 2022). The age of the ikaite columns has been estimated to approx. 600 years (Tollefsen et al., 2019), and new material is still precipitated due to continued flow of spring water (Stockmann et al., 2022). Previous studies have analyzed the chemical composition of the water inside the ikaite columns and of the surrounding seawater, which showed that the conditions inside the columns are very distinct from the seawater in both pH, salinity, temperature, alkalinity, and ion composition (Buchardt et al., 2001; Stockmann et al., 2018). The eukaryotic diversity inside the ikaite columns have only briefly been investigated previously (Stougaard et al., 2002), where seven 18S rRNA genes were isolated and sequenced. Those seven sequences showed affiliation to Ascomycota, Annelida, Bacillariophyta (Diatomea), Chlorophyta, Ciliophora, Dinophyceae (Dinoflagellata), and Mesomycetozoa (re-classified as Ichthyosporea), which were all identified in the current study.

Studies of eukaryotic microbial diversity in freshwater environments are not as abundant as in marine environments but have been carried out in, e.g., Arctic and Antarctic cryoconite holes (Cameron et al., 2012; Jaarsma et al., 2023), Patagonian and Antarctic lakes (Schiaffino et al., 2016), Lake Vostoc in the Antarctic (Gura and Rogers, 2020) and ice-covered, freshwater lakes in Subarctic Russia (Galachyants et al., 2023). The studies in general describe a rich eukaryotic diversity, including Ciliophora (ciliates), Cryptophyceae (flagellated algae), Dinophyceae (dinoflagellates), Bacillariophyceae (diatoms), Basidio- and Ascomycota (fungi), and Chlorophyta (green algae), which aligns well with our findings except that we, within the fungi, find Cryptomycota in much higher abundance that the Basidio- and Ascomycota.

Ikaite column 3 furthermore contained a large relative abundance of Phragmoplastophyta of the order Magnoliophyta, which are classified as angiosperms, or seed-producing plants. However, the phylum Phragmoplastophyta contains Embryophytes (land plants) as well as green algae closely related to land plants, including Zygnematophyceae, Coleochaetophyceae, and Charophyceae. Some of the 18S rRNA genes from the ikaite columns classified as belonging to the Zygnematophyceae, whereas others classified as being more closely related to land plants. As marine vascular plants do exist, this may either be due to contamination of our samples or due to the limited content of 18S rRNA databases.

Using different substrates and different concentrations of nutrients did make a difference in the cultured prokaryotic population. For example, ranging from full-strength R2 over 10x diluted R2 to 100x diluted R2 showed a unique relative abundance of bacterial orders for each concentration, and three bacterial phyla were unique to the 100x diluted R2 substrates. Complex substrates like olive oil and algal polymers resulted in very distinct populations, also compared to the R2-based substrates. In a related study, the same samples were plated on R2-agar substrates containing the same range of carbohydrates. This resulted in a very low diversity of cultured isolates, but did lead to the discovery of a novel, amylase-producing bacterium, selected on a diluted R2-agar substrate supplemented with amylose as sole carbon source (Zervas et al., 2024; Bendtsen et al., n.d.).

Summarizing, this study of microbial diversity inside the ikaite columns provides novel insights into the community structures and microbial distribution inside the columns. These findings suggest that the microbial communities, especially the prokaryotic community, exhibit variations caused by different physical and/or chemical conditions throughout the column structure. The presence of multiple, yet-unclassified eukaryotic and prokaryotic microorganisms further highlights that this environment is largely understudied and contains a wide range of novel, uncharacterized microorganisms.

### Potential microbial metabolic pathways in the ikaite columns

The identification of multiple genes potentially involved in the cycling of nitrogen, phosphorus, and sulfur within the HQ ikaite MAGs provides valuable insights into the microbial processes occurring in the ikaite columns. While this study did not look further into a detailed analysis of metabolic pathways, the presence of these genes and their known activities shows the potential biochemical processes within this unique environment.

For nitrogen cycling, the presence of genes associated with denitrification as well as nitrogen fixation suggests a dynamic nitrogen metabolism within the columns. Additionally, genes involved in the degradation of organic nitrogen further highlight the diversity of nitrogen sources and the potential for organic nitrogen utilization by microorganisms in this ecosystem.

In terms of phosphorus cycling, the identification of numerous genes involved in phosphate transport, mineralization of organic phosphorus, mineralization of phosphonates, solubilization of inorganic phosphorus, and P-starvation response regulation indicates a robust phosphorus metabolism within the ikaite columns. The abundance of these genes suggests that microorganisms in this environment are well-equipped to adapt to varying phosphorus availability and efficiently utilize different phosphorus sources.

The most interesting find is the extensive presence of genes involved in sulfur cycling, suggesting the importance of sulfur utilization in the microbial community within the ikaite columns. The identification of an intact Sox sulfur oxidation pathway, genes associated with assimilatory sulfate reduction, and dissimilatory sulfate reduction and oxidation pathways emphasizes the versatility of sulfur metabolism in this environment. Furthermore, the presence of genes related to amino acid metabolism, cycling of organic sulfur compounds, methane metabolism, sulfonate utilization, and modification of bacterial tRNAs highlights the multifaceted nature of sulfur cycling and its integration into various essential microbial processes. The high abundance of sulfur cycling genes and the presence of sulfur in the water inside the ikaite columns (Buchardt et al., 2001) suggests that sulfur plays a crucial role in the metabolic strategies of microorganisms within the ikaite columns. This finding leads us to propose that future research could explore the specific contributions of sulfur metabolism to the overall microbial community dynamics and ecosystem function in this unique and extreme environment combined with more detailed measurements of oxygen availability at multiple locations inside the columns. Overall, the identified genetic repertoire provides a foundation for understanding the potential ecological roles and metabolic capabilities of microorganisms thriving in ikaite columns.

### Biotechnological potential in the ikaite columns

The potential of cold-active enzymes for industrial and biotechnological purposes is significant and has resulted in an increasing attention on enzyme discovery in low-temperature environments for applications within, e.g., the pharmaceutical industry, detergent industry, food industry, and within molecular biology and research (Barocca et al., 2017).

A small selection of enzymes from the ikaite columns have previously been identified and characterized from cultured bacterial isolates and from metagenomic DNA (Schmidt et al., 2007, 2010, 2012; Schmidt and Stougaard, 2010; Vester et al., 2014; Lylloff et al., 2016) including several papers in the pipeline (Bendtsen et al., n.d.; Nowak et al., n.d.; Oliva et al., 2024). Common for these enzymes are that they all have a high relative activity at low temperatures, and the extracellular enzymes furthermore tolerate a high pH with activities up to above pH 10. These results indicated that there would still be a great potential for enzyme discovery in the ikaite columns. Therefore, with the current study we chose to apply a metagenome sequence-based approach on enzyme discovery.

The identification of carbohydrate-active enzymes within the ikaite columns provides valuable insight into the potential and ecological functions of microbial communities associated with these unique mineral formations and showcased the diversity of enzymatic activities dedicated to carbohydrate metabolism. Among the various CAZyme categories, glycosyl hydrolases play an important role in catalyzing the hydrolysis of glycosidic bonds in diverse substrates. The identification of 97 different GH families within the 25 HQ MAGs alone highlights the significance of these enzymes in the ikaite microbiome. Among the most abundant GH families 1, 2, 3, 6, 13, 19, 23, 28, 37, and 43, 72, 73, and 133, we find range of activities including α-glucosidase, α/β-galactosidase, α/β-glucanase, β-xylanase, cellobiohydrolase, glycosyltransferase, galacturonase, trehalase, glucosaminidase, and transglucosidase, to mention a few. As a detailed study of sequence similarities to know enzyme sequences is beyond the scope of this paper, we will just conclude that the presence of multiple CAZyme genes and the dominance of specific GH families suggested a specialized and adapted enzymatic machinery that plays a crucial role in the microbial ecology of the ikaite microorganisms. Further investigations into the biochemical properties and ecological roles of these enzymes will undoubtedly contribute to our understanding of microbial community dynamics in extreme environments and may unlock novel biotechnological applications, including biofuel production, bioremediation, and the food industry.

In conclusion, this study demonstrates the great diversity of mainly uncharacterized prokaryotic and eukaryotic microorganisms within the ikaite columns in the Ikka Fjord in SW Greenland. As the majority of these microorganisms are expectedly adapted to life at constant low temperature and high pH, the metagenome furthermore shows a vastly untapped biotechnological potential of the resident microbial communities for enzymatic applications where low temperature or high pH is favorable.

## Data availability statement

The original contributions presented in the study are included in the article/Supplementary material, further inquiries can be directed to the corresponding author.

## Author contributions

MT: Conceptualization, Data curation, Formal analysis, Funding acquisition, Investigation, Methodology, Project administration, Supervision, Visualization, Writing – original draft. AZ: Data curation, Formal analysis, Investigation, Methodology, Software, Visualization, Writing – original draft. PS: Conceptualization, Supervision, Writing – review & editing. LE-J: Data curation, Formal analysis, Investigation, Methodology, Software, Visualization, Writing – original draft.

## Footnotes

^1^ www.rproject.org

^2^ https://zenodo.org/badge/latestdoi/546561474

^3^ https://github.com/rrwick/Porechop

^4^ https://zenodo.org/doi/10.5281/zenodo.10254965

^5^ https://github.com/qichao1984/NCyc

^6^ https://github.com/qichao1984/SCycDB

^7^ https://github.com/ZengJiaxiong/Phosphorus-cycling-database
