# Supplementary material for: Investigating eukaryotic and prokaryotic diversity and functional potential in the cold and alkaline ikaite columns in Greenland
Source: Front Microbiol. 2024 Apr 9;15:1358787. doi: 10.3389/fmicb.2024.1358787 (PMC11035741; doi:10.3389/fmicb.2024.1358787)
Supplement: Supplementary file 1 [file Data_Sheet_1.zip › Data Sheet 1 - 2024-03-11T121721.464.PDF]

Supplementary tables

Supplementary table S1. Location of ikaite column fragments samples for this study.

**Column ID**	**Location in column**	**Sample depth (m below surface)**
Column 1	Top	6.0
	Middle	10.3
	Bottom	13.3
Column 2	Top	2.7
	Middle	6.5
	Bottom	10.0
Column 3	Top	3.9
	Middle	10.1
	Bottom	13.3

Supplementary table S2. Alpha diversity of the prokaryotic communities in three ikaite columns at three different sample heights.

**Samples**	**observed_features**	**faith_pd**	**pielou_evenness**
Column1 - Top (n=5)	192 ± 49	24.1 ± 3.4	0.65 ± 0.06
Column1 - Middle (n=5)	369 ± 88	33.1 ± 4.3	0.74 ± 0.04
Column1 - Bottom (n=4)	255 ± 50	24.5 ± 4.1	0.78 ± 0.01
Column2 - Top (n=5)	177 ± 82	20.7 ± 6.5	0.72 ± 0.10
Column2 - Middle (n=5)	150 ± 43	17.5 ± 4.0	0.62 ± 0.02
Column2 - Bottom (n=5)	287 ± 114	29.7 ± 7.6	0.73 ± 0.05
Column3 - Top (n=5)	144 ± 17	18.2 ± 1.2	0.65 ± 0.05
Column3 - Middle (n=3)	175 ± 26	21.1 ± 2.1	0.71 ± 0.02
Column3 - Bottom (n=2)	351 ± 168	29.4 ± 7.3	0.73 ± 0.06

Supplementary Table S3. Relative abundance (%) of archaea in the ikaite columns identified with standard 16S rRNA primers. Archaeal phyla were identified in all column locations: Columns 1, 2 and 3; T (top), M (middle), and B (bottom) samples.

**Phylum**<br>**-** Order	**Col 1 T**	**Col 1 M**	**Col 1 B**	**Col 2 T**	**Col 2 M**	**Col 2 B**	**Col 3 T**	**Col 3 M**	**Col 3 B**
**Unknown archaea**	0	0	0	0	0	0.079	0	0	0
**Aenigmarchaeota**									
- Aenigmarchaeales	0.065	0	0	0	0	0	0	0	0
**Crenarchaeota**									
- Nitrosopumilales	0.021	0.278	0,021	0.024	0	0.815	0	0.151	0.268
**Halobacterota**									
- Methanomicrobiales	0	0	0	0	0	0	0	0.038	0
**Nanoarchaeota**									
- Woesearchaeales	0.177	0.247	0	0.537	0.014	0.884	0.006	0.074	0
**Thermoplasmatota**									
- Marine Group II	0.038	0.006	0	0	0	0.010	0	0.040	0

Supplementary table S4. Alpha diversity of the eukaryotic communities in three ikaite columns at three different sample heights.

Samples	observed_features	faith_pd	pielou_evenness
Column1 - Top (n=5)	301 ± 58	35.4 ± 4.8	0.65 ± 0.10
Column1 - Middle (n=5)	230 ± 62	28.9 ± 7.7	0.77 ± 0.11
Column1 - Bottom (n=5)	226 ± 43	28.8 ± 5.5	0.72 ± 0.05
Column2 - Top (n=5)	166 ± 92	23.0 ± 10.2	0.67 ± 0.04
Column2 - Middle (n=5)	157 ± 66	20.2 ± 6.4	0.64 ± 0.06
Column2 - Bottom (n=5)	370 ± 48	40.3 ± 4.3	0.78 ± 0.02
Column3 - Top (n=5)	98 ± 37	17.1 ± 5.7	0.54 ± 0.12
Column3 - Middle (n=5)	151 ± 125	19.3 ± 14.6	0.51 ± 0.22
Column3 - Bottom (n=4)	159 ± 175	18.6 ± 18.8	0.61 ± 0.23

Supplementary table S5. Alpha diversity of the prokaryotic communities in the enrichment cultures (T1=195 days) and the inculcation material (T0 = Column 1, Middle).

Sample / substrate	observed_features	faith_pd	pielou_evenness
T0 (n=5)	369 ± 87	28.0 ± 3.8	0.74 ± 0.04
T1_R2_gluc (n=2)	19 ± 8	3.4 ± 0.4	0.44 ± 0.24
T1_R2_10_gluc (n=2)	32 ± 2	4.5 ± 0.2	0.58 ± 0.06
T1_R2_100_gluc (n=3)	37 ± 9	4.8 ± 0.5	0.60 ± 0.06
T1_R2_10_starch (n=2)	33 ± 7	4.3 ± 0.7	0.62 ± 0.09
T1_R2_10_cel (n=2)	24 ± 4	3.9 ± 0.2	0.59 ± 0.00
T1_R2_10_algae (n=2)	23 ± 3	3.6 ± 0.4	0.62 ± 0.03
T1_IW_cas_pyr (n=2)	13 ± 3	2.5 ± 0.0	0.35 ± 0.16
T1_R2_100_lip (n=3)	7 ± 3	2.5 ± 0.2	0.18 ± 0.03

Supplementary table S6. Quality control of the high-quality MAGs identified in the ikaite metagenome.

			CheckM Results			
**Assembly**	**Total length (>= 0 bp)**	**# contigs**	**Marker lineage**	**# markers**	**Completeness**	**Contamination**
**bin_01**	4,573,710	143	k__Bacteria (UID1453)	171	94.21	2.99
**bin_02**	2,927,951	50	c__Betaproteobacteria (UID3959)	414	95.45	1.65
**bin_03**	3,788,500	72	c__Gammaproteobacteria (UID4274)	581	96.98	2.87
**bin_08**	3,216,012	154	c__Gammaproteobacteria (UID4267)	544	91.16	5.31
**bin_10**	2,984,529	182	o__Clostridiales (UID1120)	250	78.90	1.46
**bin_11**	2,385,581	41	p__Cyanobacteria (UID2143)	472	99.18	0.27
**bin_16**	3,363,257	80	f__Rhodobacteraceae (UID3340)	568	85.64	1.48
**bin_17**	2,936,558	147	p__Bacteroidetes (UID2605)	316	96.90	2.86
**bin_24**	4,071,152	23	c__Gammaproteobacteria (UID4274)	581	99.94	1.55
**bin_26**	5,257,215	275	p__Bacteroidetes (UID2605)	316	98.02	7.38
**bin_29**	7,920,047	32	p__Cyanobacteria (UID2192)	584	99.45	0.98
**bin_30**	3,675,055	33	f__Rhodobacteraceae (UID3340)	568	99.21	0.00
**bin_32**	3,541,196	60	k__Bacteria (UID1452)	163	94.24	3.64
**bin_33**	4,015,361	102	c__Gammaproteobacteria (UID4274)	581	96.55	2.39
**bin_36**	4,941,055	33	k__Bacteria (UID2569)	278	99.46	2.15
**bin_37**	4,002,428	208	o__Rhizobiales (UID3450)	513	94.77	5.05
**bin_38**	3,879,718	113	c__Deltaproteobacteria (UID3216)	247	96.00	3.87
**bin_39**	2,532,105	183	p__Firmicutes (UID241)	213	91.30	3.04
**bin_41**	2,029,254	83	p__Firmicutes (UID241)	213	89.41	2.76
**bin_48**	2,219,504	63	p__Actinobacteria (UID1454)	206	95.92	1.53
**bin_52**	4,070,254	402	o__Rhizobiales (UID3447)	416	93.37	8.02
**bin_61**	3,633,465	57	k__Bacteria (UID2569)	278	95.70	2.71
**bin_65**	3,916,373	56	k__Bacteria (UID1453)	171	94.37	3.42
**bin_68**	2,079,782	59	p__Firmicutes (UID241)	213	84.96	4.24
**bin_71**	3,239,933	76	c__Betaproteobacteria (UID3959)	413	99.04	1.38

Supplementary table S7. Phylogenetic identification of the high-quality MAGs identified in the ikaite metagenome based on BLAST result on identifier genes 16S rRNA, *gyr*B, *rpo*A, and *rpo*D.

Assembly	16S	gyrB	rpoA	rpoD
**bin_01**	Actinomycetota (Acidimicrobiales)	Actinomycetota (Acidimicrobiales)	Actinomycetota (Acidimicrobiales)	Actinomycetota (Acidimicrobiales)
**bin_02**	n/a	Pseudomonadota (Nitrosomonadales)	Pseudomonadota (Thiobacillus?)	Pseudomonadota (Thiobacillus?)
**bin_03**	n/a	Pseudomonadota (Methylococcaceae)	Pseudomonadota	Pseudomonadota (Thioalkalivibrio?)
**bin_08**	Pseudomonadota	Pseudomonadota	n/a	Pseudomonadota
**bin_10**	n/a	Inconclusive	Inconclusive	n/a
**bin_11**	n/a	Synechococcus	Synechococcus	Synechococcus
**bin_16**	n/a	Pseudomonadota (Rhodobacterales)	Pseudomonadota (Rhodobacterales)	Pseudomonadota (Rhodobacterales)
**bin_17**	n/a	Bacteroidota (Bacteroidales)	n/a	n/a
**bin_24**	Pseudomonadota (Thioalkalivibrio?)	Pseudomonadota	Pseudomonadota (Thioalkalivibrio?)	Pseudomonadota (Thioalkalivibrio?)
**bin_26**	n/a	Bacteroidota	n/a	n/a
**bin_29**	n/a	Cyanobacteria (Oscillatoria)	Cyanobacteria (Oscillatoria)	n/a
**bin_30**	n/a	Pseudomonadota (Rhodobacterales)	Pseudomonadota (Rhodobacterales)	Pseudomonadota (Rhodobacterales)
**bin_32**	Chloroflexota	Chloroflexota (Anaerolineales)	Chloroflexota (Anaerolineales)	Chloroflexota (Anaerolineales)
**bin_33**	n/a	Pseudomonadota (Ectothiorhodospiraceae)	Pseudomonadota (Ectothiorhodospiraceae)	Pseudomonadota (Thioalkalivibrio?)
**bin_36**	Bacteroidota	Bacteroidota (Flavobacteriales)	Bacteroidota (Flavobacteriales)	n/a
**bin_37**	n/a	Pseudomonadota (Mesorhizobium)	Mesorhizobium	Mesorhizobium
**bin_38**	n/a	Thermodesulfobacteriota (Desulfobacterales)	Thermodesulfobacteriota (Desulfobacterales)	Thermodesulfobacteriota (Desulfobacterales)
**bin_39**	n/a	Bacillota	Bacillota	n/a
**bin_41**	Bacillota (Paenibacillus?)	Bacillota (Paenibacillus?)	Bacillota (Oscillospiraceae)	n/a
**bin_48**	Actinomycetota (Coriobacteriales)	Actinomycetota	Actinomycetota	n/a
**bin_52**	n/a	Pseudomonadota (Methylococcaceae)	Pseudomonadota (Methylococcaceae)	Pseudomonadota (Rhizobiales)
**bin_61**	n/a	Bacteroidota (Bacteroidales)	Bacteroidota	n/a
**bin_65**	Actinomycetota (Acidimicrobiales)	Actinomycetota (Acidimicrobiales)	n/a	Actinomycetota
**bin_68**	n/a	Bacillota	n/a	n/a
**bin_71**	Nitrospirota (Nitrospirales)	Nitrospirota (Nitrospirales)	Inconclusive	Inconclusive

Supplementary table S8. Genes involved in cycling of nitrogen (N), phosphorous (P), and sulfur (P) identified in the ikaite HQ MAGs.

**N cycling**		**P cycling**				**S cycling**					
hzsB	nirB	ADE2	pgtA	phoX	purS	acuI	dddL	hdrB2	phsA	soxL	tsdB
NR	nirD	RegX3	pgtB	phpC	purT	acuN	dddP	hdrC1	phsB	soxX	ttrA
narB	nrfA	SenX3	phnA	phy	pyk	aprA	dddT	hdrC2	phsC	soxY	ttrB
narC	nrfC	adk	phnC	pit	pyrE	aprB	dmdA	hdrD	prpE	soxZ	ttrC
nasA	nrfD	cmk	phnD	ppa	pyrF	asrA	dmdB	hpsK	psrA	sqdB	tusA
nasB	hao	dcd	phnD_phnN	ppc	pyrG	asrB	dmdC	hpsL	psrC	sqdX	tusB
nirA	nxrB	deoB	phnE	ppd	pyrH	asrC	dmdD	hpsM	pta	sqr	tusC
napA	nifD	dut	phnF	ppdK	rpiA	betA	dmoA	hpsN	qmoA	sseA	tusD
napB	nifH	fomC	phnG	ppk	rtpR	betB	dmsA	hpsO	qmoB	ssuA	tusE
napC	nifK	gcd	phnH	pps	spoT	betC	dmsB	hpsP	qmoC	ssuB	xsc
narG	nifW	gdh	phnI	ppx	thyA	comA	dmsC	hydA	rdlA	ssuC	yihQ
narH	ansB	glpQ	phnJ	prsA	tmk	comB	doxD	hydB	rdsr	ssuD	
narI	asnB	glpT	phnK	pstA	ugpA	comC	dsrA	hydD	sat	ssuE	
narJ	gdh_K00260	gmk	phnL	pstB	ugpB	comD	dsrB	iseK	sbp	sudA	
narV	gdh_K00261	gnd	phnM	pstC	ugpC	comE	dsrC	iseL	sfnG	sudB	
narY	gdh_K00262	gnl	phnN	pstS	ugpE	cuyA	dsrE	iseM	sgpB	sulP	
narZ	gdh_K15371	gntK	phnO	ptsH	ugpQ	cysA	dsrF	isfD	sgpC	suyA	
nirK	glnA	guaA	phnP	ptsI	ushA	cysC	dsrH	mccB	shyA	suyB	
nirS	glsA	guaB	phnPP	ptxB		cysD	dsrK	mddA	shyC	tauA	
norB	gs_K00264	lysR	phnS	ptxD		cysE	dsrL	mdh	sir	tauB	
norC	gs_K00265	ndk	phnU	purA		cysH	dsrM	metA	slcC	tauC	
nosZ	gs_K00266	nrdA	phnV	purB		cysI	dsrN	metB	slcD	tauD	
napA	gs_K00284	nrdB	phnW	purC		cysJ	dsrO	metC	soeA	tauY	
napB	nao	nrdD	phnZ	purD		cysK	dsrP	metX	soeB	tauZ	
napC	nmo	nrdE	phny	purE		cysM	dsyB	metY	soeC	tbuB	
narG	ureA	nrdF	phoA	purF		cysN	fccA	metZ	sor	tbuC	
narH	ureB	nrdJ	phoB	purH		cysN_cysC	fccB	msmA	sorA	tmm	
narI	ureC	opd	phoD	purK		cysP	gdh	msmB	sorB	tmoC	
narJ	hcp	pbfA	phoH	purL		cysQ	glpE	mtsA	soxA	tmoF	
narV	pmoA	pckA	phoP	purM		cysU	hdrA1	mtsB	soxB	touF	
narY		pckG	phoR	purN		cysW	hdrA2	nrnA	soxC	tpa	
narZ		pepM	phoU	purQ		cysZ	hdrB1	otr	soxD	tsdA	

Supplementary table S9. Genes including function involved in cycling of nitrogen identified in each of the high-quality MAGs from the ikaite columns.

Function	Gene	M1	M2	M3	M8	M10	M11	M16	M17	M24	M26	M29	M30	M32	M33	M36	M37	M38	M39	M41	M48	M52	M61	M65	M68	M71
Anammox	hzsB	0	0	0	1	0	0	0	0	0	0	0	0	0	0	0	0	0	0	0	0	0	0	0	0	0
Assimilatory nitrate reduction	NR	1	1	1	1	0	1	1	0	1	1	1	1	1	1	0	1	1	0	1	1	0	0	1	1	1
Assimilatory nitrate reduction	narB	1	1	1	1	0	1	1	0	1	1	1	1	1	1	0	1	1	1	1	1	1	0	1	1	1
Assimilatory nitrate reduction	narC	1	0	1	1	0	1	1	0	1	0	1	1	1	1	0	1	0	0	0	0	1	0	1	0	1
Assimilatory nitrate reduction	nasA	1	1	1	1	1	1	1	1	0	1	1	1	1	1	1	1	1	1	1	1	1	0	1	1	1
Assimilatory nitrate reduction	nasB	1	1	1	1	1	1	1	1	1	1	1	1	1	1	1	1	1	1	1	1	1	1	1	1	1
Assimilatory nitrate reduction	nirA	1	1	1	1	1	1	1	1	1	1	1	1	1	1	1	1	1	1	1	1	1	1	1	1	1
Denitrification	napA	0	0	1	1	1	1	1	0	0	1	1	1	1	1	1	1	1	1	1	0	1	0	1	1	1
Denitrification	napB	0	1	1	0	0	0	0	0	1	0	0	0	0	1	0	1	0	0	0	0	0	0	0	0	0
Denitrification	napC	0	0	1	0	0	0	1	0	1	1	0	0	1	1	1	1	0	0	0	1	0	0	0	0	1
Denitrification	narG	0	0	1	0	0	0	0	0	0	1	0	0	0	1	0	1	0	1	0	0	1	0	0	0	1
Denitrification	narH	0	1	1	0	1	1	1	0	1	1	1	1	0	1	0	0	0	1	1	1	1	0	0	1	1
Denitrification	narI	0	1	1	0	1	0	1	0	1	0	0	1	0	1	0	1	1	0	1	0	1	0	0	0	1
Denitrification	narJ	0	1	1	1	1	0	1	0	1	0	0	0	0	1	1	1	1	0	0	0	1	1	1	0	1
Denitrification	narV	0	0	0	0	0	0	0	0	0	0	0	0	0	0	0	0	0	0	0	0	1	0	0	0	0
Denitrification	narY	1	0	0	0	0	1	0	0	0	0	0	0	0	1	0	0	1	0	0	0	0	0	1	0	0
Denitrification	narZ	0	1	0	0	0	0	1	0	1	0	0	0	0	0	0	0	0	0	0	0	0	0	0	0	0
Denitrification	nirK	1	1	1	1	1	1	1	1	1	1	1	1	1	1	1	1	1	1	1	1	1	1	1	1	1
Denitrification	nirS	1	1	1	1	1	1	1	1	1	1	1	1	1	1	1	1	1	1	1	1	1	1	1	1	1
Denitrification	norB	0	1	1	1	0	0	1	1	1	1	1	1	1	1	0	1	1	1	0	0	0	1	1	1	1
Denitrification	norC	0	0	0	1	0	0	1	0	0	0	0	0	0	0	0	0	0	0	0	0	0	0	0	0	0
Denitrification	nosZ	1	1	1	1	1	1	1	1	1	1	1	1	1	1	1	1	1	1	1	1	1	1	1	1	1
Dissimilatory nitrate reduction	napA	0	0	1	1	1	1	1	0	0	1	1	1	1	1	1	1	1	1	1	0	1	0	1	1	1
Dissimilatory nitrate reduction	napB	0	1	1	0	0	0	0	0	1	0	0	0	0	1	0	1	0	0	0	0	0	0	0	0	0
Dissimilatory nitrate reduction	napC	0	0	1	0	0	0	1	0	1	1	0	0	1	1	1	1	0	0	0	1	0	0	0	0	1
Dissimilatory nitrate reduction	narG	0	0	1	0	0	0	0	0	0	1	0	0	0	1	0	1	0	1	0	0	1	0	0	0	1
Dissimilatory nitrate reduction	narH	0	1	1	0	1	1	1	0	1	1	1	1	0	1	0	0	0	1	1	1	1	0	0	1	1
Dissimilatory nitrate reduction	narI	0	1	1	0	1	0	1	0	1	0	0	1	0	1	0	1	1	0	1	0	1	0	0	0	1
Dissimilatory nitrate reduction	narJ	0	1	1	1	1	0	1	0	1	0	0	0	0	1	1	1	1	0	0	0	1	1	1	0	1
Dissimilatory nitrate reduction	narV	0	0	0	0	0	0	0	0	0	0	0	0	0	0	0	0	0	0	0	0	1	0	0	0	0
Dissimilatory nitrate reduction	narY	1	0	0	0	0	1	0	0	0	0	0	0	0	1	0	0	1	0	0	0	0	0	1	0	0
Dissimilatory nitrate reduction	narZ	0	1	0	0	0	0	1	0	1	0	0	0	0	0	0	0	0	0	0	0	0	0	0	0	0
Dissimilatory nitrate reduction	nirB	1	1	1	1	1	1	1	0	1	1	1	1	1	1	1	1	1	1	1	1	1	0	1	1	1
Dissimilatory nitrate reduction	nirD	1	1	1	1	1	1	1	0	1	1	1	1	1	1	1	1	0	1	0	1	1	0	1	1	1
Dissimilatory nitrate reduction	nrfA	0	1	1	1	1	0	1	1	1	1	0	0	1	1	1	1	1	0	0	1	0	1	1	0	0
Dissimilatory nitrate reduction	nrfC	1	1	1	1	1	0	1	1	1	1	0	1	1	1	1	1	1	1	1	1	1	1	1	1	1
Dissimilatory nitrate reduction	nrfD	0	0	1	0	0	0	0	0	0	1	0	0	0	1	1	1	0	0	0	1	0	0	0	0	0
Nitrification	hao	0	0	0	1	1	0	0	0	0	0	0	0	0	0	0	0	0	0	0	1	0	0	0	0	0

Nitrification	nxrB	0	0	0	0	0	0	0	0	0	0	0	0	1	0	0	0	0	0	0	0	0	0	0	0	0
Nitrogen fixation	nifD	0	0	0	0	0	1	0	0	0	0	1	0	1	1	0	1	0	1	1	0	0	1	1	1	0
Nitrogen fixation	nifH	1	1	1	1	1	1	1	1	1	1	1	1	1	1	1	1	1	1	1	1	1	1	1	1	1
Nitrogen fixation	nifK	0	0	0	0	0	0	0	0	0	0	0	1	0	1	0	0	0	1	0	0	0	0	0	1	0
Nitrogen fixation	nifW	1	1	1	1	1	1	1	1	1	1	1	1	1	1	1	1	1	1	1	1	1	1	1	1	1
Organic degradation and synthesis	ansB	0	0	1	1	1	0	1	1	1	1	0	0	1	1	1	1	1	1	1	0	0	1	0	0	1
Organic degradation and synthesis	asnB	1	1	1	1	1	1	1	1	1	1	1	1	1	1	1	1	1	1	1	1	1	1	1	1	1
Organic degradation and synthesis	gdh_K00260	0	0	0	0	1	0	0	0	0	0	0	0	0	0	0	0	0	1	1	0	0	0	0	1	0
Organic degradation and synthesis	gdh_K00261	1	1	1	1	1	1	1	1	1	1	1	1	1	1	1	1	1	1	1	1	1	1	1	1	1
Organic degradation and synthesis	gdh_K00262	1	1	1	1	1	1	1	1	1	1	1	1	1	1	1	1	1	1	0	1	1	1	1	1	1
Organic degradation and synthesis	gdh_K15371	1	1	1	1	1	1	1	1	1	1	1	1	1	1	1	1	1	1	1	1	1	1	1	1	1
Organic degradation and synthesis	glnA	1	1	1	1	1	1	1	1	1	1	1	1	1	1	1	1	1	1	1	1	1	1	1	1	1
Organic degradation and synthesis	glsA	1	1	1	1	1	1	1	1	1	1	1	1	1	1	1	1	1	1	1	1	1	1	1	1	1
Organic degradation and synthesis	gs_K00264	0	0	0	0	0	0	0	0	0	0	0	1	1	0	0	1	0	0	0	0	1	0	0	0	0
Organic degradation and synthesis	gs_K00265	1	1	1	1	0	0	1	1	1	1	1	1	1	1	0	1	1	1	1	1	1	0	1	1	1
Organic degradation and synthesis	gs_K00266	1	1	1	1	1	1	1	1	1	1	1	1	1	1	1	1	1	1	1	1	1	1	1	1	1
Organic degradation and synthesis	gs_K00284	1	1	1	0	0	1	1	0	1	0	1	0	1	0	0	0	0	0	1	0	0	0	1	0	1
Organic degradation and synthesis	nao	0	0	0	0	0	0	0	1	0	0	0	0	0	0	0	0	0	0	0	0	0	0	0	0	0
Organic degradation and synthesis	nmo	1	1	1	1	1	1	1	1	1	1	1	1	1	1	1	1	1	1	1	1	1	1	1	1	1
Organic degradation and synthesis	ureA	1	0	1	1	1	1	1	0	0	1	1	1	1	1	1	1	0	1	1	1	1	1	1	1	1
Organic degradation and synthesis	ureB	1	1	1	1	1	1	1	1	1	1	1	1	1	1	1	1	1	1	1	1	1	1	1	1	1
Organic degradation and synthesis	ureC	1	1	1	1	1	1	1	0	1	1	1	1	1	1	1	1	1	1	1	1	1	1	1	1	1
Others	hcp	1	1	1	1	1	1	1	1	1	1	1	1	1	1	1	1	1	1	1	1	1	1	1	1	1
Others	pmoA	0	0	0	0	0	0	1	0	0	0	0	0	0	0	0	0	0	0	0	0	0	0	0	0	0

Supplementary table S10. Genes involved in cycling of sulfur identified in each of the high-quality MAGs from the ikaite columns.

Gene	M1	M2	M3	M8	M10	M11	M16	M17	M24	M26	M29	M30	M32	M33	M36	M37	M38	M39	M41	M48	M52	M61	M65	M68	M71
acuI	1	1	1	1	1	1	1	0	1	1	1	1	1	1	1	1	1	1	0	1	1	1	1	0	1
acuN	0	0	0	0	0	0	0	0	0	0	0	0	0	0	0	0	0	0	0	0	1	0	0	0	0
aprA	1	1	1	0	1	0	1	1	0	1	0	1	0	1	1	1	1	1	1	0	1	1	1	0	0
aprB	0	1	1	0	0	0	0	0	0	0	0	0	0	1	0	1	1	0	0	0	0	0	0	0	0
asrA	0	1	1	1	0	1	1	0	0	0	1	1	0	1	0	1	1	1	0	0	1	0	1	1	1
asrB	1	1	1	0	1	0	1	0	1	1	1	0	0	1	0	1	1	0	0	1	0	1	1	1	1
asrC	0	0	0	0	1	0	1	0	0	1	0	1	0	0	1	0	1	1	1	1	0	1	0	1	1
betA	1	1	1	1	1	1	1	1	1	1	1	1	1	1	1	1	1	1	1	1	1	1	1	1	1
betB	1	1	1	1	1	1	1	1	1	1	1	1	1	1	1	1	1	1	1	1	1	1	1	1	1
betC	1	1	0	0	0	1	1	0	0	1	1	1	1	1	1	1	0	1	0	0	1	0	1	0	1
comA	0	0	0	0	0	0	0	1	0	1	0	0	0	0	1	0	0	0	0	0	0	0	0	0	0
comB	1	1	1	1	1	1	1	1	1	1	1	1	1	1	1	1	1	0	1	0	0	1	1	1	1
comC	0	0	0	0	0	0	1	0	0	0	0	1	0	0	0	0	0	0	0	0	1	0	0	0	0
comD	0	0	0	0	0	0	0	0	0	0	0	0	0	0	0	1	0	0	0	0	0	1	0	0	0
comE	1	0	0	0	0	0	0	0	0	0	1	0	1	0	0	0	1	1	0	0	0	0	1	1	0
cuyA	1	1	1	1	1	1	1	1	1	1	1	1	1	1	1	1	1	1	1	1	1	1	1	1	1
cysA	1	1	1	1	1	0	1	1	1	1	1	1	1	1	1	1	1	1	1	1	1	1	1	1	1
cysC	1	1	1	1	1	1	1	1	1	1	1	1	1	1	1	1	1	1	1	1	1	1	1	1	1
cysD	1	1	1	1	1	1	1	1	1	1	1	1	1	1	1	1	1	1	1	1	1	1	1	1	1
cysE	1	1	1	1	1	1	1	1	1	1	1	1	1	1	1	1	1	1	1	1	1	1	1	1	1
cysH	1	1	1	1	1	1	1	1	1	1	1	1	1	1	1	1	0	1	1	1	1	1	1	0	1
cysI	1	1	1	1	1	1	0	1	1	1	1	1	1	0	1	1	1	1	1	1	1	1	1	1	1
cysJ	1	1	1	1	1	1	1	1	1	1	1	1	1	1	1	1	1	1	1	1	1	1	1	1	1
cysK	1	1	1	1	1	1	1	1	1	1	1	1	1	1	1	1	1	1	1	1	1	1	1	1	1
cysM	1	1	1	1	1	1	1	1	1	1	1	1	1	1	1	1	1	1	1	1	1	1	1	1	1
cysN	1	1	1	1	1	1	1	1	1	1	1	1	1	1	1	1	1	1	1	1	1	1	1	1	1
cysN_cysD	1	1	1	1	1	1	1	1	1	1	1	1	1	1	1	1	1	1	1	1	1	1	1	1	1
cysP	1	1	1	0	1	1	1	0	1	0	1	1	1	1	1	1	1	1	1	1	1	0	0	1	1
cysQ	1	1	1	1	1	1	1	1	1	1	1	1	1	1	1	1	1	1	1	1	1	1	1	1	1
cysU	1	1	1	1	1	1	1	1	1	1	1	1	1	1	1	1	1	1	1	1	1	1	1	1	1
cysW	1	1	1	1	0	0	1	0	1	1	1	1	1	1	1	0	0	0	0	1	0	0	1	0	1
cysZ	0	1	1	1	1	0	0	1	1	1	0	1	1	1	0	1	1	1	0	0	1	0	0	0	0
dddL	0	0	0	0	0	0	0	0	0	0	0	0	0	0	0	0	0	0	0	0	0	0	0	0	1
dddP	1	0	1	1	1	1	1	1	1	1	1	1	1	1	1	1	1	1	1	1	1	1	0	1	1
dddT	1	0	1	0	1	0	1	0	1	0	0	1	0	1	0	1	1	1	0	0	1	0	0	0	1
dmdA	1	1	1	1	1	1	0	1	1	1	1	1	0	1	1	1	0	1	1	1	0	1	1	1	1
dmdB	1	1	1	1	0	0	1	0	1	1	1	1	1	1	1	1	1	1	0	1	1	1	1	1	1
dmdC	1	0	1	1	0	0	1	1	1	1	0	1	1	1	1	1	1	1	0	0	1	1	1	1	1
dmdD	1	0	0	0	0	0	1	0	1	1	0	1	0	0	1	1	1	1	0	0	1	0	1	1	1
dmoA	0	0	0	0	0	0	1	0	0	0	0	1	0	0	0	0	0	0	0	0	0	0	1	0	1
dmsA	1	1	1	1	1	1	1	1	1	1	1	1	1	1	1	1	1	1	1	1	1	1	1	1	1

dmsB	1	1	1	0	1	0	0	1	1	1	1	0	1	1	1	1	1	1	1	1	1	1	1	1	1
dmsC	1	1	1	1	1	1	1	1	1	1	1	1	1	1	1	1	1	1	1	1	1	1	1	1	1
doxD	0	0	0	0	0	0	0	0	0	0	0	0	0	0	0	0	0	0	0	0	1	0	0	0	0
dsrA	1	1	1	1	1	0	1	1	0	1	1	1	1	1	1	1	1	1	1	0	1	1	0	1	1
dsrB	1	1	0	1	1	0	0	1	0	1	0	1	0	1	1	1	1	1	0	0	0	1	1	0	1
dsrC	0	1	0	0	0	0	0	0	0	0	0	0	1	0	0	0	1	0	0	0	0	0	0	0	1
dsrE	0	1	1	0	0	0	0	0	0	0	0	0	0	1	0	1	0	0	0	0	0	0	0	1	0
dsrF	0	1	1	0	0	0	0	0	0	0	0	0	0	1	0	1	0	0	0	0	0	0	0	1	0
dsrH	0	1	1	0	0	0	0	0	0	0	0	0	0	1	0	1	0	0	0	0	0	0	0	0	0
dsrK	0	1	1	0	0	0	0	0	1	0	0	0	0	1	0	1	1	0	0	0	0	0	0	0	0
dsrL	0	1	1	0	0	0	0	0	0	0	0	0	0	1	0	1	1	0	0	0	0	0	0	0	0
dsrM	0	1	1	0	0	0	0	0	1	0	0	0	0	1	1	1	1	0	0	0	1	0	0	0	1
dsrN	0	1	1	0	0	1	0	0	0	0	1	0	0	1	0	1	1	0	0	0	1	0	0	0	1
dsrO	0	0	1	0	0	0	0	0	0	0	0	0	0	1	0	1	0	0	0	0	0	0	0	0	0
dsrP	0	1	1	1	0	0	0	0	0	0	0	0	0	1	0	1	1	0	0	1	0	0	0	0	1
dsyB	1	1	1	0	0	0	1	0	1	0	1	0	0	1	0	1	1	0	1	1	1	0	1	0	1
fccA	0	1	1	1	0	0	0	0	1	0	0	1	0	1	0	1	0	0	0	0	0	1	0	0	1
fccB	1	1	1	1	0	1	1	1	0	1	1	1	0	1	1	1	0	1	0	0	0	0	0	1	1
gdh	1	0	1	1	1	0	1	1	1	1	1	1	1	1	1	1	1	1	1	1	0	1	1	1	1
glpE	1	1	1	1	1	1	1	1	1	1	1	1	1	1	1	1	1	1	1	1	1	1	1	1	1
hdrA1	0	0	0	0	1	0	0	1	1	1	0	0	0	1	1	1	1	1	0	0	0	1	1	1	0
hdrA2	0	0	0	0	0	0	1	0	0	0	0	0	1	0	0	0	1	1	1	0	0	0	0	1	0
hdrB1	0	0	0	0	0	0	0	0	0	0	1	0	1	1	0	0	1	1	0	1	0	0	0	1	0
hdrB2	0	1	0	0	0	0	0	0	0	0	0	0	0	1	0	1	1	0	0	0	0	0	0	0	0
hdrC1	0	0	0	0	0	0	0	0	0	0	0	0	0	1	0	1	1	0	0	0	0	0	0	0	0
hdrC2	0	1	0	0	0	0	0	0	0	0	0	0	1	0	0	0	1	1	0	0	0	0	0	1	0
hdrD	1	1	1	1	0	0	0	0	0	0	1	1	1	1	1	1	1	1	0	1	0	1	1	1	1
hpsK	0	0	0	0	1	0	1	0	0	0	0	1	0	0	0	0	0	1	0	0	0	0	0	0	1
hpsL	0	0	0	0	0	0	1	0	0	0	0	1	0	0	0	0	0	0	0	0	1	0	0	0	0
hpsM	0	0	0	0	1	0	1	0	0	1	0	1	0	0	1	0	0	1	0	0	1	0	0	0	1
hpsN	1	1	1	1	1	1	0	0	1	1	1	1	1	1	1	1	1	0	0	1	1	0	1	0	1
hpsO	0	0	1	1	0	0	1	0	0	1	1	1	0	0	0	0	1	0	0	0	0	0	0	0	1
hpsP	1	1	1	0	1	1	1	0	1	1	1	1	0	1	1	1	1	1	0	0	1	0	1	1	1
hydA	0	0	0	0	0	0	0	0	0	0	1	0	0	1	0	0	0	0	0	0	0	0	1	0	0
hydB	0	0	0	0	0	0	0	0	0	0	0	0	0	0	0	0	0	0	0	0	0	1	0	0	0
hydD	0	1	1	1	0	0	0	0	0	0	0	0	1	1	0	1	1	0	0	0	0	0	1	0	0
iseK	0	0	0	0	1	0	0	0	0	0	0	1	0	0	0	0	0	0	0	0	0	0	0	0	0
iseL	0	0	0	0	0	0	0	0	0	0	0	1	0	0	0	0	0	0	0	0	0	0	0	0	0
iseM	0	1	0	1	1	0	1	0	0	0	1	1	0	1	0	1	1	1	0	0	1	0	0	0	1
isfD	1	0	1	1	1	1	1	1	1	1	1	1	1	1	1	1	1	1	1	1	1	1	1	0	1
mccB	1	0	0	0	1	0	0	0	1	1	1	0	0	0	1	0	1	0	0	0	1	0	1	0	0
mddA	0	1	0	0	0	0	0	0	0	0	0	1	1	0	0	0	1	0	0	0	1	0	0	0	0
mdh	1	1	1	1	1	1	1	1	1	1	1	1	1	1	1	1	1	1	1	1	1	1	1	1	1
metA	1	1	1	1	1	0	1	0	1	0	1	1	1	1	1	1	0	1	0	1	1	0	0	0	1

metB	1	1	1	1	1	1	1	1	1	1	1	1	1	1	1	1	1	1	1	0	1	1	1	0	1
metC	1	1	1	1	1	1	1	1	1	1	1	1	1	1	1	1	1	1	1	1	1	1	1	1	1
metX	1	0	1	1	1	1	1	1	1	1	1	1	0	1	1	0	1	1	1	1	1	0	1	1	1
metY	1	1	1	1	1	0	1	0	1	1	1	0	1	1	1	1	1	1	0	1	1	0	1	1	1
metZ	1	1	1	1	1	1	1	1	1	1	1	1	1	1	1	1	1	1	1	1	1	1	1	1	1
msmA	1	0	1	0	0	0	1	0	0	0	0	1	0	0	0	0	0	0	0	0	0	0	1	0	1
msmB	0	0	1	0	0	0	0	0	0	0	0	0	0	0	0	1	0	0	0	0	0	0	0	0	0
mtsA	0	0	0	0	0	0	0	0	0	1	0	0	0	0	0	0	0	0	0	0	0	0	0	0	0
mtsB	0	0	0	0	1	0	1	0	1	1	0	1	1	0	0	0	1	1	0	1	0	0	0	1	1
nrnA	0	1	1	1	1	0	0	1	1	1	1	0	1	1	1	1	1	1	1	1	1	1	1	1	0
otr	0	0	0	0	0	0	0	0	0	1	0	0	1	0	0	0	0	0	0	0	0	1	0	0	0
phsA	1	1	1	1	0	0	0	0	1	1	0	0	1	1	1	1	1	0	0	0	0	0	0	0	0
phsB	0	1	1	1	0	0	0	0	1	0	0	0	0	1	0	1	0	1	0	0	0	0	0	0	0
phsC	0	0	1	0	0	0	0	0	0	1	0	0	1	0	0	0	0	0	0	0	1	1	0	0	1
prpE	1	1	1	1	0	1	1	1	1	1	1	1	1	1	1	1	1	1	0	1	1	1	1	1	1
psrA	0	0	0	0	0	0	0	0	0	0	0	0	0	0	0	0	0	0	0	0	0	0	0	1	0
psrC	0	0	0	0	0	0	0	0	0	0	0	0	0	0	1	0	0	0	0	0	0	0	0	0	0
pta	1	1	1	1	1	1	1	1	1	1	1	1	1	1	1	1	1	1	1	1	1	1	1	1	1
qmoA	0	1	0	0	0	0	1	0	0	0	0	1	0	0	1	0	1	0	1	0	1	0	0	0	1
qmoB	0	1	0	0	0	0	0	0	0	0	0	0	0	0	0	0	1	0	0	0	0	0	0	0	1
qmoC	0	0	0	0	0	0	0	0	0	0	0	0	0	0	0	0	1	0	0	0	0	0	0	0	0
rdlA	0	0	0	0	1	0	0	0	0	1	0	0	0	0	0	0	1	1	0	0	0	1	0	0	0
rdsr	0	0	1	0	0	0	0	0	0	0	0	0	0	1	0	1	0	0	0	0	0	0	0	0	0
sat	1	1	1	1	1	1	1	1	1	1	1	1	1	1	1	1	1	1	1	1	1	1	1	1	1
sbp	0	0	0	1	0	1	1	1	0	1	1	0	1	1	1	1	0	0	0	0	1	1	0	0	1
sfnG	0	0	0	0	0	0	0	0	0	0	0	0	0	0	0	0	0	0	0	0	0	0	1	0	0
sgpB	0	1	1	1	1	0	0	1	1	1	0	0	0	1	1	1	1	0	0	0	0	1	0	0	1
sgpC	0	1	1	1	0	0	1	0	1	1	0	1	1	1	1	1	1	0	0	0	1	1	0	0	1
shyA	0	1	1	1	0	0	0	0	0	0	0	0	1	1	0	1	0	0	0	1	0	0	0	0	0
shyC	0	1	1	0	0	0	0	1	1	0	0	1	0	0	0	1	1	1	0	1	0	1	1	1	0
sir	1	1	1	1	1	1	1	0	1	1	1	1	1	1	1	1	0	0	1	1	1	0	1	1	1
slcC	1	1	1	1	1	1	1	1	1	1	1	1	1	1	1	1	1	1	1	0	1	1	1	1	1
slcD	1	0	0	0	1	0	1	0	1	0	0	1	0	1	0	0	1	0	0	0	1	0	1	0	1
soeA	0	1	1	0	0	0	1	0	1	1	0	1	0	1	0	1	0	0	0	1	0	0	0	0	1
soeB	0	0	1	0	0	0	1	0	0	0	0	1	0	0	0	0	0	0	0	0	0	0	0	0	1
soeC	0	1	1	0	0	0	0	0	0	0	0	1	0	1	0	1	0	0	0	0	0	0	0	0	1
sor	0	0	1	0	0	0	0	0	0	0	0	0	0	1	0	1	0	0	0	0	0	0	0	0	0
sorA	1	0	0	0	0	0	0	0	0	0	0	0	0	1	0	0	0	0	0	0	0	0	1	0	1
sorB	0	0	0	0	0	0	1	0	1	0	0	0	0	0	0	0	0	0	0	0	0	0	0	0	1
soxA	0	1	1	1	1	0	0	0	1	0	0	1	1	1	1	1	0	1	0	1	0	1	0	0	1
soxB	1	1	1	0	1	1	1	0	1	1	1	1	1	1	1	1	1	1	1	0	1	1	1	1	1
soxC	1	0	1	1	0	1	1	0	1	0	1	1	1	1	0	1	0	0	0	0	1	0	1	0	1
soxD	1	1	1	0	0	1	0	0	1	0	0	1	0	1	0	1	0	0	0	0	0	0	0	0	1
soxL	0	0	0	0	0	0	0	0	0	0	0	0	0	0	0	1	0	0	0	0	0	0	0	0	0

soxX	1	1	1	1	0	0	1	0	1	0	1	1	0	1	1	1	0	0	0	0	1	0	0	1	1
soxY	0	1	1	0	1	0	0	0	0	0	0	1	0	1	1	1	0	0	0	0	1	1	0	1	1
soxZ	0	1	1	1	1	1	1	0	0	0	1	1	0	1	0	1	0	0	0	0	1	0	0	0	1
sqdB	1	0	0	0	0	1	1	1	1	1	1	1	1	1	1	1	1	0	1	0	1	1	1	0	1
sqdX	1	1	1	1	1	1	1	1	1	1	1	1	1	1	1	1	1	1	1	1	1	1	1	1	1
sqr	0	1	1	0	0	0	0	0	1	0	0	0	1	0	0	1	0	0	0	0	0	0	0	0	1
sseA	0	1	1	0	0	0	1	0	1	1	0	1	0	1	0	1	0	0	0	1	1	1	1	0	1
ssuA	1	1	1	1	1	1	1	1	1	1	1	1	1	1	1	1	1	1	1	1	1	1	1	1	1
ssuB	1	1	1	1	1	1	1	1	1	1	1	1	1	1	1	1	1	1	1	1	1	1	1	1	1
ssuC	1	1	1	1	1	1	1	1	1	1	1	1	1	1	1	1	1	1	1	1	1	1	1	1	1
ssuD	1	1	1	1	1	0	1	1	1	1	1	1	1	1	1	1	1	1	1	1	1	1	1	1	1
ssuE	1	1	0	1	1	1	1	1	1	1	1	1	0	1	1	1	1	1	1	0	1	1	1	1	1
sudA	1	1	1	1	1	1	1	1	1	1	1	1	1	1	1	1	1	1	1	1	1	1	1	1	1
sudB	0	0	0	0	1	0	0	0	1	0	0	0	0	1	0	0	1	1	1	1	0	0	0	0	0
sulP	1	1	1	1	1	1	1	1	1	1	1	1	1	1	1	1	1	1	1	1	1	0	1	1	1
suyA	0	0	0	0	1	0	0	0	0	0	0	1	0	0	0	0	0	1	0	0	0	0	0	0	1
suyB	0	0	0	0	0	0	0	0	0	0	0	1	1	0	0	0	0	1	0	0	0	0	0	0	1
tauA	1	1	1	1	1	1	1	0	1	0	1	1	1	1	1	1	1	1	1	1	0	1	1	1	1
tauB	1	1	1	1	1	1	1	1	1	1	1	1	1	1	1	1	1	1	1	1	1	1	1	1	1
tauC	0	0	1	0	0	0	0	0	0	0	0	1	1	0	0	0	0	0	0	1	1	0	0	0	1
tauD	1	1	1	1	1	1	1	1	1	1	1	1	0	1	1	1	1	1	0	1	1	1	1	1	1
tauY	0	0	0	0	0	0	0	0	0	0	0	1	0	0	0	0	0	0	0	0	1	0	0	0	0
tauZ	0	0	0	0	0	0	0	0	1	0	0	1	0	0	0	0	0	1	1	0	1	0	1	0	1
tbuB	0	0	0	0	0	0	0	0	1	0	0	0	0	0	0	0	0	0	0	0	0	0	0	0	0
tbuC	0	1	1	0	1	0	0	1	0	1	0	0	0	1	0	1	0	1	1	1	0	1	0	0	0
tmm	0	0	0	0	0	0	0	0	0	0	0	1	0	0	0	0	0	0	0	0	1	0	0	0	0
tmoC	1	0	1	0	0	0	0	0	0	0	0	0	1	1	0	1	0	0	0	1	0	0	1	0	1
tmoF	0	0	0	0	0	1	0	0	0	0	1	0	0	0	0	0	0	0	0	0	0	0	0	0	0
touF	0	1	0	0	0	0	1	0	0	0	0	0	0	0	0	1	0	0	0	0	0	0	0	0	0
tpa	1	1	1	1	1	1	1	1	0	1	0	1	1	1	1	0	1	1	1	1	1	1	1	1	1
tsdA	1	1	1	1	1	1	1	1	1	1	1	1	1	1	1	1	1	1	1	1	1	1	1	0	1
tsdB	0	1	1	1	0	0	0	0	1	0	0	1	0	1	0	1	0	0	0	0	1	0	0	0	0
ttrA	1	1	1	1	1	0	1	1	1	0	1	1	0	0	0	0	1	1	1	1	1	1	1	1	1
ttrB	0	1	1	0	1	1	0	0	0	1	1	0	1	1	1	1	1	1	1	1	0	1	0	1	1
ttrC	0	1	1	1	0	1	1	0	1	0	0	0	0	1	0	1	1	0	0	0	0	0	0	0	1
tusA	1	1	1	1	1	0	1	1	1	1	1	1	1	1	1	1	1	1	1	1	1	1	1	1	1
tusB	1	1	1	0	1	0	1	1	0	1	1	1	1	0	1	0	1	1	1	1	1	1	0	1	1
tusC	0	0	1	0	0	0	0	0	0	0	1	0	0	1	0	1	1	0	0	0	0	0	0	0	0
tusD	0	1	1	0	1	1	1	1	0	1	1	1	0	1	1	1	1	1	1	1	1	1	0	1	1
tusE	0	1	1	0	1	0	0	0	1	1	1	0	1	1	0	1	0	0	1	0	0	1	0	0	1
xsc	1	1	1	1	1	1	1	0	1	1	1	1	0	1	0	1	1	1	1	1	1	1	1	1	1
yihQ	1	0	0	0	0	0	0	0	0	1	0	0	1	0	0	0	0	0	0	0	0	0	0	0	0

Supplementary table S11. Genes involved in cycling of phosphorous identified in each of the high-quality MAGs from the ikaite columns.

Gene	M1	M2	M3	M8	M10	M11	M16	M17	M24	M26	M29	M30	M32	M33	M36	M37	M38	M39	M41	M48	M52	M61	M65	M68	M71
ADE2	0	0	0	0	0	0	0	1	0	1	0	1	0	0	0	0	0	0	1	1	0	1	0	0	1
RegX3	1	1	1	1	0	0	1	1	1	1	1	1	1	0	1	1	1	0	0	1	1	1	1	0	1
SenX3	1	0	0	0	0	1	0	1	0	0	1	1	1	1	0	1	1	1	0	1	0	1	0	1	1
adk	0	1	1	1	1	1	1	1	1	1	1	1	1	1	1	1	1	1	1	1	1	1	0	1	1
cmk	1	1	1	1	1	1	1	1	1	1	1	1	1	1	1	1	1	1	1	1	1	1	1	1	1
dcd	1	0	1	1	1	1	1	0	0	0	0	1	0	0	0	0	0	0	0	0	1	0	0	0	1
deoB	0	0	0	0	0	0	0	0	0	0	0	1	0	0	0	0	0	1	0	0	0	0	0	1	1
dut	1	1	1	1	1	1	0	1	1	1	1	1	0	1	1	1	1	1	1	1	1	1	1	1	1
fomC	0	0	0	0	0	0	0	0	0	1	0	0	0	0	0	0	0	0	0	0	0	0	0	0	0
gcd	1	1	1	0	1	0	0	0	0	0	1	1	0	1	1	0	1	0	1	0	1	0	0	0	1
gdh	1	0	0	0	0	0	0	0	1	1	1	1	0	0	0	0	1	1	0	0	1	0	0	0	1
glpQ	1	1	0	1	1	0	1	1	0	1	1	1	1	1	1	0	1	1	1	1	1	1	1	1	1
glpT	0	0	0	0	1	0	0	0	1	0	0	0	1	1	0	0	0	1	1	0	0	0	1	0	0
gmk	1	1	1	1	0	0	1	0	1	1	1	1	0	1	0	1	1	1	1	0	1	1	1	1	1
gnd	1	1	1	1	1	1	1	0	1	0	1	1	1	1	1	1	0	1	1	1	1	0	1	0	1
gnl	1	0	0	1	0	1	0	1	1	1	0	0	1	1	1	0	0	0	0	0	1	0	1	0	1
gntK	1	1	1	1	1	1	1	1	1	1	1	1	1	1	1	0	1	0	1	1	1	1	1	1	1
guaA	1	1	1	1	1	1	1	0	1	1	1	1	1	1	1	1	1	1	0	1	1	1	1	0	1
guaB	1	1	1	1	0	1	1	1	1	1	1	1	1	1	1	1	1	1	1	1	1	1	1	1	1
lysR	1	1	1	1	1	1	1	0	1	1	1	1	1	1	0	1	1	1	1	1	1	1	1	1	1
ndk	0	1	0	1	1	1	1	1	1	1	1	1	0	0	1	1	0	1	0	0	1	1	1	0	1
nrdA	0	1	1	1	0	0	1	0	1	0	0	1	1	1	0	1	0	0	0	0	1	0	1	0	1
nrdB	0	1	1	1	0	0	0	0	0	0	0	0	0	1	0	0	0	0	0	0	0	0	1	0	0
nrdD	0	1	0	0	1	0	0	0	0	0	0	0	0	0	0	0	1	1	1	1	0	0	0	1	0
nrdE	0	0	0	0	0	0	1	0	0	0	0	0	0	0	0	0	0	0	0	0	0	0	0	0	0
nrdF	1	0	0	0	0	0	1	0	0	0	0	1	0	0	0	0	1	0	0	0	0	0	0	0	1
nrdJ	0	0	0	0	0	0	1	1	1	1	1	1	0	0	1	0	0	1	1	1	1	1	1	0	0
opd	0	0	0	0	0	0	0	0	0	1	0	0	1	0	0	0	0	0	0	0	0	0	0	0	0
pbfA	1	1	1	1	1	1	1	1	1	1	1	1	1	1	1	1	1	1	1	1	1	1	1	1	0
pckA	0	0	0	0	1	0	1	1	1	1	1	1	0	0	1	0	1	0	1	0	0	1	0	0	1
pckG	1	1	1	1	0	1	1	1	0	1	1	1	1	1	1	1	1	1	1	0	1	1	1	1	1
pepM	0	1	1	1	0	1	0	1	1	1	1	1	0	1	1	1	1	1	1	0	1	1	1	0	1
pgtA	0	0	0	0	0	1	1	0	0	0	0	1	0	0	0	0	0	0	0	0	0	0	0	0	0
pgtB	0	0	0	0	0	0	0	0	0	0	0	0	0	0	0	0	0	0	1	0	0	0	0	0	0
phnA	0	0	0	0	0	0	0	0	0	0	0	0	0	0	0	0	0	0	0	0	0	0	0	0	1
phnC	1	0	1	0	0	1	0	0	1	0	0	1	1	1	0	1	1	0	0	0	1	0	1	0	1
phnD	0	0	0	0	0	0	0	0	0	1	0	1	0	1	0	0	0	0	0	0	1	0	0	0	1
phnD_phnN	0	0	1	0	0	1	0	0	0	0	0	0	0	1	0	0	0	0	0	0	0	0	0	0	1
phnE	0	0	0	0	0	1	0	0	0	0	0	1	0	1	0	1	0	0	0	0	1	0	0	0	1
phnF	1	0	0	0	1	0	1	0	0	0	0	1	1	0	1	0	0	1	0	0	1	0	0	1	1
phnG	0	0	0	0	0	0	0	0	0	0	0	1	0	0	0	0	0	0	0	0	0	0	1	0	1

phnH	0	0	0	0	0	0	0	0	0	0	0	1	0	0	0	0	0	0	0	0	0	0	1	0	1
phnI	1	0	0	0	0	0	0	0	0	0	0	1	0	0	1	1	0	0	0	1	0	0	1	0	1
phnJ	1	0	0	0	0	0	0	0	0	0	0	1	0	0	0	0	0	0	0	0	0	0	1	0	1
phnK	0	0	0	0	0	0	0	0	0	0	0	0	0	0	0	0	0	0	0	0	0	0	1	0	1
phnL	1	0	0	0	0	0	0	0	0	0	0	0	0	0	0	0	0	0	0	0	0	0	1	0	1
phnM	1	1	1	1	0	0	1	0	1	0	0	1	0	1	1	1	1	1	0	0	0	0	1	0	1
phnN	0	0	0	0	1	0	0	0	0	0	0	1	0	0	0	0	0	0	0	0	0	0	0	0	1
phnO	1	0	0	0	0	0	0	0	0	0	0	0	1	0	1	0	0	0	0	0	0	0	1	1	0
phnP	1	1	0	0	0	0	1	1	1	1	1	1	1	1	1	0	1	1	1	1	1	1	0	0	1
phnPP	0	0	0	0	1	1	0	0	0	1	1	1	0	0	0	0	1	1	0	1	0	1	0	1	0
phnS	1	0	1	0	1	1	0	0	1	1	0	1	0	0	1	0	0	0	0	0	1	1	0	0	1
phnU	0	0	0	0	0	0	0	0	1	0	0	0	0	0	1	0	0	0	0	0	0	0	0	0	0
phnV	0	0	0	1	0	0	1	0	0	0	1	1	0	0	0	0	0	0	0	0	0	0	0	0	1
phnW	1	0	1	0	1	1	0	0	1	0	1	1	1	1	1	1	1	0	1	1	1	1	1	1	1
phnZ	0	0	0	0	0	0	0	0	0	0	0	0	0	0	0	0	0	0	0	0	0	0	1	0	0
phny	0	0	0	1	0	0	0	0	0	0	0	0	0	0	0	0	0	0	0	0	0	0	0	0	1
phoA	1	0	0	0	1	0	0	0	0	1	1	0	0	0	1	0	1	0	0	1	0	1	0	1	0
phoB	1	1	1	1	1	1	1	1	1	1	1	1	1	1	1	1	1	1	1	1	1	1	1	1	1
phoD	1	0	1	0	0	0	0	0	0	1	1	0	0	1	1	1	0	0	0	0	0	1	1	0	0
phoH	1	1	1	1	1	1	1	1	1	1	1	1	0	1	1	1	1	1	1	0	1	1	1	1	1
phoP	1	1	1	1	1	1	1	1	1	1	1	0	1	1	1	1	1	1	1	1	1	1	1	1	1
phoR	1	1	1	1	1	1	1	1	1	1	1	1	1	1	1	1	1	1	1	1	1	1	1	1	1
phoU	1	0	1	1	1	0	1	0	0	0	1	1	1	1	0	1	1	1	1	1	1	0	1	0	1
phoX	1	0	1	1	0	0	0	1	0	1	1	1	0	1	0	1	0	0	0	0	0	1	1	0	1
phpC	1	1	0	1	1	0	1	0	1	0	1	1	0	1	1	1	1	1	0	0	1	1	1	1	1
phy	1	1	0	1	1	0	1	0	1	0	1	0	1	0	0	0	0	0	0	0	0	0	0	0	0
pit	0	1	1	0	1	0	1	0	1	1	1	1	0	1	1	0	1	1	0	0	1	1	0	0	1
ppa	1	0	0	0	1	1	1	1	1	1	1	1	1	0	1	1	1	1	1	1	1	0	0	1	1
ppc	0	0	0	1	0	1	0	0	0	1	1	0	0	0	1	0	0	0	0	0	1	0	1	0	0
ppd	1	0	0	0	1	0	0	0	1	0	0	0	1	0	1	0	0	0	0	0	0	0	0	1	0
ppdK	1	0	0	0	1	0	1	0	0	0	0	1	1	0	0	0	1	0	1	1	1	1	1	0	1
ppk	1	0	1	1	1	1	1	1	0	1	1	1	0	0	1	0	0	0	0	0	1	1	1	0	1
pps	1	1	1	1	1	1	1	1	1	1	1	1	1	1	1	1	1	1	1	1	1	1	1	1	1
ppx	1	0	0	1	1	1	1	1	1	1	1	1	0	0	1	0	0	1	0	1	1	1	1	0	1
prsA	1	1	1	1	1	1	1	1	1	1	1	1	1	1	1	1	1	1	1	1	1	1	1	1	1
pstA	1	1	1	1	1	1	1	1	1	0	1	1	1	1	0	1	1	1	1	1	1	1	1	0	1
pstB	0	1	0	1	1	1	0	0	0	0	1	1	1	0	0	0	0	1	1	0	1	0	0	0	0
pstC	1	1	1	1	1	1	1	1	1	0	1	1	1	1	0	1	1	1	0	1	1	1	1	0	1
pstS	1	1	1	1	1	1	1	1	1	1	1	1	1	1	1	1	1	1	1	1	1	1	1	1	0
ptsH	1	1	1	1	1	1	1	1	1	1	1	1	1	1	0	1	1	1	1	1	1	1	1	0	1
ptsI	0	1	1	1	0	0	1	1	0	1	0	1	1	1	1	1	1	0	0	1	0	1	1	0	1
ptxB	0	1	0	0	0	0	0	0	0	0	0	0	0	0	0	1	0	0	0	0	0	0	0	0	0
ptxD	1	0	1	1	1	1	1	0	1	1	0	1	0	1	1	1	0	1	1	0	1	0	1	1	1
purA	0	1	0	1	1	1	0	0	0	0	1	1	0	0	0	0	0	1	1	0	1	0	1	1	1

purB	1	1	1	1	1	1	1	1	1	1	1	1	1	1	1	1	1	1	0	0	1	1	1	0	1
purC	1	1	1	1	1	1	1	1	1	1	1	1	1	1	1	1	1	0	1	0	1	1	1	0	0
purD	0	1	1	1	1	1	1	1	1	1	1	1	1	1	1	0	1	0	1	1	1	1	1	1	1
purE	1	0	1	0	0	1	0	0	0	0	1	1	0	0	0	0	1	1	0	0	0	0	0	1	1
purF	0	1	1	1	1	1	1	0	1	1	1	1	1	1	1	1	1	1	0	1	1	0	0	1	1
purH	0	0	0	1	0	1	0	1	0	1	1	1	1	0	0	0	1	1	0	0	1	0	0	0	1
purK	0	0	1	1	0	1	1	1	1	1	1	1	1	1	0	1	1	1	1	0	1	0	0	0	1
purL	1	1	1	1	1	1	1	1	1	1	1	1	0	1	1	1	1	1	1	0	1	1	1	1	1
purM	0	0	0	1	1	0	0	0	0	0	1	1	1	0	0	0	0	1	0	0	1	0	0	0	1
purN	0	0	1	1	1	1	0	1	0	1	1	1	0	1	1	1	1	0	1	0	1	1	1	1	1
purQ	0	0	0	0	0	1	0	0	0	0	1	1	0	1	0	0	1	1	0	0	1	0	0	0	1
purS	1	0	0	0	0	1	0	0	0	0	1	1	0	0	0	0	0	0	0	0	1	0	0	0	1
purT	0	0	1	0	0	0	0	1	0	0	1	0	0	1	0	0	0	0	0	0	0	0	0	1	0
pyk	1	1	1	1	1	1	0	1	1	1	1	1	1	1	1	1	1	1	1	1	1	1	1	1	1
pyrE	1	1	1	1	0	1	1	0	1	0	1	1	1	1	0	1	0	0	1	0	1	1	1	0	1
pyrF	1	0	0	1	0	1	0	1	1	0	1	1	1	0	1	0	0	1	0	0	0	0	0	1	1
pyrG	1	1	1	1	1	1	1	1	1	1	1	1	1	1	1	1	1	1	1	1	1	1	1	1	1
pyrH	0	0	1	1	0	1	0	0	0	0	1	1	0	0	0	0	0	0	0	0	1	0	0	0	1
rpiA	1	1	1	1	1	1	1	1	1	1	1	1	1	1	1	1	0	1	1	1	1	1	0	1	1
rtpR	1	0	1	0	1	1	0	0	0	0	1	0	0	1	1	0	1	0	0	0	0	0	0	0	0
spoT	1	1	1	1	1	1	1	1	1	1	1	1	1	1	1	1	1	1	1	1	1	1	1	1	1
thyA	1	1	1	1	1	1	1	1	1	1	1	1	1	1	0	1	1	0	1	1	1	1	1	1	1
tmk	1	1	1	1	1	1	1	1	1	0	1	1	1	1	1	1	1	1	1	1	1	1	1	1	1
ugpA	1	0	0	0	1	1	1	0	0	0	1	1	1	1	0	0	0	0	0	0	1	0	1	0	1
ugpB	1	1	0	1	0	0	1	0	0	1	0	1	0	0	0	0	1	0	0	0	0	0	1	0	1
ugpC	0	1	1	1	1	1	1	1	1	1	1	1	1	1	1	1	0	1	1	1	1	1	1	1	1
ugpE	1	0	0	0	1	1	0	0	0	0	1	1	1	1	0	0	0	0	0	1	1	0	1	0	1
ugpQ	0	0	0	0	0	0	0	0	1	0	1	0	1	0	0	0	0	0	1	0	0	0	0	0	1
ushA	0	1	1	0	1	0	1	0	0	1	1	1	1	1	1	1	1	1	1	0	1	0	0	1	1

Supplementary table S12. Genes encoding carbohydrate-active enzymes (CAZymes) identified in each of the high-quality MAGs from the ikaite columns.

	CAZy family	M1	M2	M3	M8	M10	M11	M16	M17	M24	M26	M29	M30	M32	M33	M36	M37	M38	M39	M41	M48	M52	M61	M65	M68	M71
Auxiliary Activities	AA	0	0	0	0	0	0	0	0	1	0	0	1	0	0	0	0	0	1	0	0	0	0	1	0	1
	AA1	1	1	1	1	1	1	1	1	1	1	1	1	1	1	1	1	1	1	1	1	1	1	1	1	1
	AA10	0	0	0	0	1	0	0	0	0	1	0	0	0	0	0	0	0	0	0	0	0	1	0	0	0
	AA12	0	1	0	0	0	0	0	0	0	0	0	0	0	0	0	0	0	0	0	0	1	0	0	0	0
	AA15	0	0	0	0	0	0	0	0	0	0	0	0	0	0	0	0	0	0	0	0	0	0	1	0	0
	AA2	0	0	0	0	0	0	1	0	1	0	0	1	0	1	0	1	0	0	0	1	0	0	0	0	1
	AA3	0	0	0	0	0	0	1	0	0	1	0	1	0	0	0	0	0	0	0	0	1	0	0	0	0
	AA4	1	1	1	1	1	0	1	0	1	0	0	1	1	0	1	1	1	1	1	0	1	0	1	1	1
	AA5	0	1	1	0	1	0	0	1	1	0	1	0	1	1	1	1	1	0	0	0	1	1	0	1	1
	AA6	0	1	1	0	0	0	0	0	1	0	0	0	0	1	0	1	1	0	0	0	1	0	0	0	0
	AA7	1	0	0	0	0	0	0	0	1	0	0	0	1	1	1	1	0	0	0	0	1	0	1	0	0
	AA8	0	0	0	0	0	0	0	0	0	0	0	0	1	0	0	0	0	0	0	0	0	0	0	0	0
	AA10	0	0	0	0	1	0	0	0	0	1	0	0	0	0	0	0	0	0	0	0	0	1	0	0	0
	AA12	0	1	0	0	0	0	0	0	0	0	0	0	0	0	0	0	0	0	0	0	1	0	0	0	0
	AA15	0	0	0	0	0	0	0	0	0	0	0	0	0	0	0	0	0	0	0	0	0	0	1	0	0
Carbohydrate-Binding Modules	CBM0	0	0	0	0	0	0	0	0	1	1	1	0	0	0	0	0	0	0	0	0	0	0	1	0	0
	CBM2	1	1	1	1	1	1	0	1	1	1	1	1	1	1	1	1	1	1	1	1	1	1	1	0	1
	CBM3	0	0	1	0	1	0	0	0	0	1	1	0	0	1	1	0	1	1	0	0	0	1	0	0	0
	CBM4	0	0	0	0	0	0	1	0	1	1	0	0	0	0	1	0	0	0	0	0	0	1	0	0	0
	CBM5	1	1	1	1	1	1	0	1	1	1	1	1	1	1	1	1	1	1	1	1	1	1	1	1	0
	CBM6	1	1	1	1	0	1	1	1	1	1	1	1	1	1	1	1	1	1	0	1	0	1	1	1	1
	CBM8	0	0	0	0	0	0	0	0	0	0	0	0	0	0	1	0	0	0	0	0	0	0	1	0	0
	CBM9	0	1	0	0	0	0	1	1	0	1	0	0	1	0	1	1	0	1	1	1	0	1	0	0	0
	CBM10	0	0	0	0	0	0	0	0	0	0	0	0	0	0	1	0	0	0	0	1	0	0	0	0	1
	CBM11	1	0	0	0	0	0	0	0	0	0	0	0	0	0	0	0	0	0	0	0	0	0	0	0	0
	CBM12	0	0	0	1	1	0	1	0	0	0	0	0	0	0	1	0	0	0	1	0	0	1	0	0	1
	CBM13	1	1	1	1	1	1	1	1	1	1	1	1	1	1	1	1	1	1	1	1	1	1	1	1	1
	CBM14	0	1	1	0	1	1	0	1	0	0	0	1	0	0	0	0	0	0	1	0	1	0	1	1	0
	CBM16	0	0	0	0	0	0	0	1	0	1	0	0	0	0	0	0	0	0	0	0	0	0	0	1	0
	CBM20	1	1	1	1	1	1	1	1	1	1	1	1	1	1	1	1	1	1	1	1	1	1	1	1	1
	CBM22	0	0	0	0	0	0	0	1	0	1	0	0	1	1	0	1	1	1	0	0	1	1	0	0	1
	CBM23	0	0	0	0	0	1	0	0	1	1	1	0	1	0	0	0	1	0	0	0	0	0	1	0	1
	CBM25	0	0	0	0	0	0	0	0	0	0	0	0	0	0	1	0	0	1	0	0	0	1	0	0	0
	CBM26	0	0	0	0	0	0	0	0	0	0	0	0	0	0	1	0	0	0	0	0	0	1	0	0	0
	CBM32	1	1	0	1	1	1	1	1	0	1	1	1	1	1	1	1	1	1	0	0	1	1	1	0	1
	CBM34	0	0	0	0	1	0	0	0	0	0	0	0	0	0	0	0	0	0	0	0	0	1	0	0	0
	CBM35	0	1	0	1	1	0	0	0	1	1	0	0	0	0	1	0	1	0	0	0	1	1	0	0	1
	CBM37	0	0	0	0	0	0	0	0	0	1	0	0	0	0	1	0	0	1	1	0	0	0	0	0	0
	CBM38	1	0	0	0	0	0	0	0	1	1	0	0	0	0	1	0	0	0	0	0	1	0	1	0	0
	CBM41	1	1	1	1	1	0	1	0	1	0	1	1	0	1	0	1	1	0	0	1	0	1	1	1	1
	CBM42	1	0	0	1	1	0	0	0	0	1	0	0	0	0	0	0	0	0	0	0	0	0	1	0	0
	CBM45	1	0	0	0	0	0	0	0	0	0	0	0	0	1	0	1	0	0	0	0	0	0	0	0	0

	CBM47	1	0	1	0	0	0	0	1	0	1	0	0	0	0	1	1	0	0	0	1	0	1	0	0	0
	CBM48	1	1	1	1	1	1	1	1	1	1	1	1	1	1	1	1	1	1	1	1	1	1	1	1	1
	CBM50	1	1	1	1	1	1	1	1	1	1	1	1	1	1	1	1	1	1	1	1	1	1	1	1	1
	CBM51	1	0	0	1	0	1	1	1	1	1	1	1	1	1	1	1	1	1	1	1	1	1	1	1	1
	CBM54	1	1	0	0	1	0	0	0	0	1	1	1	1	0	1	0	1	1	0	0	0	1	0	1	1
	CBM56	0	0	0	0	0	0	0	0	0	0	0	0	0	0	0	0	0	0	0	0	1	0	0	0	0
	CBM57	0	0	0	0	0	1	0	1	1	1	1	0	0	0	1	1	1	0	0	0	1	1	0	0	0
	CBM61	0	0	0	0	0	0	0	0	0	0	0	0	1	0	0	0	0	1	0	0	0	1	0	0	0
	CBM62	0	0	0	0	0	0	0	0	0	0	1	0	0	1	1	0	0	0	0	0	0	0	0	0	0
	CBM66	1	1	1	1	1	0	1	0	0	1	1	0	1	1	1	1	0	1	1	1	0	0	1	0	0
	CBM67	1	0	0	0	0	1	1	0	0	1	0	1	0	0	0	1	0	0	0	0	0	0	0	0	0
	CBM70	0	0	0	0	1	0	0	0	0	0	0	0	0	0	0	0	0	0	0	0	0	0	0	0	0
	CBM73	1	0	0	0	0	0	0	1	0	0	0	1	0	0	0	0	0	0	0	0	0	0	0	0	0
	CBM77	0	0	0	0	0	0	0	1	0	0	0	0	0	0	0	0	0	0	0	0	0	0	0	0	0
	CBM84	0	0	0	0	0	0	0	0	0	0	0	0	0	0	0	0	0	0	0	0	0	1	0	0	0
Carbohydrate Esterases	CE0	1	0	1	0	0	0	0	1	1	1	1	0	0	1	1	1	0	1	1	0	0	1	1	0	0
	CE1	1	1	1	1	0	1	1	0	1	1	1	1	1	1	1	1	1	0	1	0	1	1	1	1	1
	CE2	0	0	0	0	0	0	0	0	0	0	0	0	0	0	1	0	0	0	0	0	0	0	0	0	0
	CE3	0	1	0	0	0	0	0	0	0	1	1	1	0	1	1	0	1	0	0	0	0	0	0	0	0
	CE4	1	1	1	1	1	1	1	1	1	1	1	1	1	1	1	1	1	1	1	1	1	1	1	1	1
	CE6	1	0	0	0	0	0	0	0	0	1	0	0	0	0	0	0	0	0	0	0	0	0	0	0	0
	CE7	0	0	0	0	0	0	0	0	1	1	0	0	1	0	0	0	1	0	1	0	0	0	1	1	0
	CE8	1	0	0	0	0	0	1	1	1	1	1	0	0	0	1	0	1	0	0	0	0	1	1	1	1
	CE9	1	0	1	0	1	1	1	1	1	1	1	1	1	0	1	0	1	1	1	1	1	1	1	1	1
	CE11	1	1	1	1	1	1	1	1	1	1	1	1	1	1	1	1	1	1	1	1	1	1	1	1	1
	CE12	1	0	1	0	0	0	0	1	1	0	0	1	1	1	1	1	0	0	0	0	0	1	1	0	1
	CE14	1	0	1	1	1	0	1	0	1	1	1	1	1	1	1	1	1	1	1	1	1	1	1	1	1
	CE15	0	0	0	0	0	0	0	0	0	1	0	0	0	0	0	0	0	0	0	0	0	0	0	0	0
	CE16	0	0	0	0	1	0	0	0	0	0	0	0	0	0	0	0	0	0	0	0	0	0	0	0	0
Glycoside Hydrolases	GH0	1	1	1	1	1	1	1	1	1	1	1	1	1	1	1	1	1	1	1	1	1	1	1	1	1
	GH1	1	1	1	1	1	1	0	1	1	1	1	1	1	1	1	1	1	1	1	1	1	1	1	1	1
	GH2	1	1	1	1	1	1	1	1	1	1	1	1	1	1	1	1	1	1	1	1	1	1	1	1	1
	GH3	1	1	1	1	1	1	1	1	1	1	1	1	1	1	1	1	1	1	1	1	1	1	1	1	1
	GH4	1	0	0	0	0	0	0	0	0	0	1	1	1	0	0	0	1	0	0	0	0	0	1	0	0
	GH5	1	0	1	0	0	0	0	0	0	0	1	1	1	0	0	0	0	0	0	0	0	0	1	0	1
	GH6	1	1	1	1	1	1	1	1	1	1	1	1	1	1	1	1	1	1	1	1	1	1	1	1	1
	GH8	0	0	0	0	0	0	1	1	0	0	0	1	0	0	1	0	0	0	0	0	0	1	0	0	0
	GH9	0	0	0	0	0	0	0	0	0	1	0	0	0	1	1	0	1	0	1	0	0	1	1	0	0
	GH10	1	0	0	0	0	0	1	1	0	1	1	0	1	1	1	1	1	1	0	0	1	1	1	0	1
	GH11	0	0	1	0	0	0	0	0	0	0	0	0	0	0	0	0	0	0	0	0	0	0	0	0	0
	GH12	0	1	0	0	0	0	1	0	0	0	1	1	0	0	0	0	0	0	0	0	1	0	1	0	1
	GH13	1	1	1	1	1	0	1	1	1	1	1	1	1	1	1	1	1	1	1	1	1	1	1	1	1
	GH15	1	0	1	0	0	1	0	0	1	1	0	0	1	0	0	0	1	0	0	0	1	0	0	0	0
	GH16	1	0	0	1	1	1	1	1	1	1	1	1	1	1	1	0	1	1	0	0	1	1	1	1	1
	GH17	0	0	0	0	1	1	0	0	1	1	1	1	1	1	1	1	1	1	1	0	0	1	0	1	0
	GH18	1	1	1	1	1	1	1	1	1	1	1	1	1	1	1	0	1	1	1	1	1	1	1	0	1

GH19	1	1	1	1	1	1	1	1	1	1	1	1	1	1	1	1	1	1	1	1	1	1	1	1	1
GH20	1	0	0	0	1	0	0	1	0	1	1	0	1	0	1	0	0	1	1	0	0	0	1	1	0
GH23	1	1	1	1	1	1	1	1	1	1	1	1	1	1	1	1	1	1	1	1	1	1	1	1	1
GH24	0	1	0	1	1	1	1	0	1	0	1	1	0	1	0	0	0	0	0	0	1	1	0	0	1
GH25	1	0	0	0	0	0	1	1	0	0	1	1	0	0	0	0	0	0	0	0	1	0	1	0	1
GH26	0	0	0	0	1	0	1	0	0	0	0	0	1	0	0	0	0	0	0	0	0	0	0	0	1
GH27	0	0	0	0	0	0	0	0	0	0	0	0	1	0	0	0	0	0	0	0	0	0	0	0	0
GH28	1	1	1	1	1	1	1	1	1	1	1	1	1	1	1	1	1	1	1	1	1	1	1	1	1
GH29	1	0	0	0	0	0	0	0	0	1	0	0	0	0	1	0	0	0	0	0	0	0	1	0	0
GH30	1	0	0	0	0	0	0	0	0	0	0	0	0	0	0	0	0	1	0	0	0	0	1	0	0
GH31	1	1	1	1	1	0	1	0	1	1	1	1	1	1	1	1	1	1	1	1	1	1	1	1	1
GH32	1	0	0	0	1	1	0	0	0	1	1	1	0	0	1	0	0	0	1	0	0	0	1	0	1
GH33	1	1	1	0	1	1	1	0	0	1	1	0	0	1	1	1	1	1	0	1	1	1	0	0	0
GH35	1	0	0	0	0	0	0	1	0	1	0	1	0	0	1	0	0	0	0	0	0	1	0	0	0
GH36	1	1	0	1	0	1	1	1	1	1	1	1	1	1	1	1	1	1	0	0	1	0	1	1	1
GH37	1	1	1	1	1	1	1	1	1	1	1	1	1	1	1	1	1	1	1	1	1	1	1	1	1
GH38	1	0	0	0	0	1	0	0	0	1	1	0	0	0	1	0	0	0	1	0	0	0	1	0	0
GH39	0	1	0	0	0	0	1	0	1	0	1	0	1	0	1	0	0	0	0	0	1	1	1	0	1
GH42	0	0	0	0	0	0	0	1	0	1	0	1	0	0	0	0	0	0	0	0	0	0	0	0	0
GH43	1	0	0	0	0	0	0	0	0	1	0	0	0	1	0	0	0	0	0	0	1	0	1	0	0
GH47	0	1	1	1	0	0	0	0	1	0	0	1	0	1	0	1	0	0	0	0	1	0	0	0	0
GH50	0	0	0	0	0	0	0	0	0	1	0	0	0	0	0	0	0	0	0	0	0	0	0	0	0
GH51	0	0	0	0	0	0	0	0	0	1	0	0	1	0	1	0	0	0	1	0	0	0	1	0	0
GH53	1	1	1	1	1	0	0	0	1	0	1	1	1	1	1	1	1	1	0	1	1	1	1	1	1
GH55	1	0	0	1	1	1	1	1	0	1	1	1	1	0	1	1	1	1	1	1	1	1	1	0	1
GH57	1	1	1	1	0	1	0	1	0	1	1	0	0	1	1	1	0	1	0	0	0	1	0	0	0
GH58	0	1	0	0	0	0	0	0	0	0	0	0	0	0	0	0	0	0	0	0	0	0	0	0	0
GH63	1	0	0	0	0	0	0	0	0	0	0	0	1	0	0	0	0	0	0	0	0	0	0	0	0
GH64	0	0	0	0	0	0	0	0	0	0	0	0	0	0	1	0	0	0	0	0	0	0	0	0	0
GH65	1	1	1	1	1	0	1	1	1	1	1	1	1	1	1	1	1	0	1	1	1	1	1	0	1
GH66	0	0	0	0	0	0	0	0	0	0	1	0	0	0	0	0	0	0	0	0	0	0	0	0	0
GH71	0	1	1	1	0	0	0	0	1	0	0	0	0	1	0	1	0	0	0	1	0	0	1	0	0
GH72	1	1	1	1	1	1	1	1	1	1	1	1	1	1	1	1	0	1	1	1	1	1	1	1	1
GH73	1	1	1	1	1	1	1	1	1	1	1	0	1	1	1	1	1	1	1	0	1	1	1	1	1
GH74	0	0	0	0	1	0	0	0	0	0	1	0	0	0	1	0	1	0	0	0	0	0	0	0	0
GH76	1	0	0	0	0	0	0	0	0	1	1	0	0	0	0	0	0	0	0	0	0	0	1	0	1
GH77	1	1	1	0	1	1	0	1	1	1	1	0	1	1	1	1	1	1	0	0	1	1	1	0	1
GH78	1	1	1	1	1	1	1	1	1	1	1	0	1	1	1	1	1	1	0	0	1	1	0	1	1
GH81	0	0	0	0	0	0	0	0	0	0	0	0	0	0	0	0	1	0	0	0	0	0	0	0	0
GH84	1	0	0	0	0	0	0	0	1	0	0	0	1	1	0	0	0	0	0	0	0	0	0	0	0
GH86	0	0	0	0	0	0	0	0	0	0	0	0	0	0	1	0	0	0	0	0	0	0	0	0	0
GH88	0	0	0	0	0	0	0	0	0	1	0	0	0	0	0	0	0	0	0	0	0	0	0	0	0
GH89	0	0	0	0	0	0	0	0	0	0	1	0	0	0	1	0	0	0	0	1	0	0	0	0	0
GH92	1	0	1	0	0	0	0	0	0	1	1	1	0	1	1	1	0	1	1	0	1	1	1	0	0
GH93	0	0	0	0	0	0	0	0	0	0	1	1	0	0	0	0	0	1	0	0	1	0	0	0	0
GH94	0	1	1	1	0	0	1	0	1	1	0	1	1	1	1	0	1	0	0	1	0	1	0	0	1

	GH95	1	0	0	0	1	1	0	1	1	1	1	0	0	0	1	0	0	0	1	0	0	1	1	0	1
	GH97	0	0	0	0	0	0	0	1	0	0	0	0	0	0	0	0	0	0	0	0	0	1	0	0	0
	GH99	0	0	0	0	0	1	0	0	1	1	0	0	1	1	1	1	0	0	0	1	1	0	0	0	1
	GH100	0	0	0	0	0	1	0	0	0	0	1	0	0	0	0	0	0	0	0	0	0	0	0	0	0
	GH101	1	0	0	0	1	0	1	1	1	1	0	1	1	0	1	0	1	1	1	0	1	1	1	1	1
	GH102	1	1	1	1	0	1	1	0	1	0	0	1	0	1	0	1	1	0	0	1	1	0	1	1	1
	GH103	0	1	1	1	0	0	1	0	1	0	0	1	0	1	0	1	1	0	0	0	1	0	1	0	1
	GH104	0	0	1	0	0	1	0	0	0	0	1	0	0	0	0	0	0	0	1	0	0	0	0	0	0
	GH105	0	0	0	0	1	1	1	0	0	1	0	0	1	1	1	0	0	1	1	0	1	0	0	0	1
	GH106	0	0	0	0	0	0	0	0	0	1	0	0	0	0	0	0	0	0	0	0	0	0	0	0	0
	GH108	0	0	1	1	0	0	1	0	0	0	1	1	0	0	0	1	0	0	0	0	0	0	0	0	0
	GH109	1	0	0	0	1	1	1	0	1	1	0	1	0	0	1	0	0	0	0	0	0	1	1	1	1
	GH114	0	1	0	1	0	0	0	1	0	1	1	0	0	0	1	0	1	0	1	0	0	1	0	0	1
	GH116	0	0	0	0	0	1	0	0	0	0	1	0	0	0	0	0	0	0	0	0	0	0	0	0	0
	GH117	0	0	0	0	0	0	0	0	0	1	0	0	0	0	0	1	0	0	0	0	0	0	0	0	0
	GH123	0	0	0	0	0	0	0	0	0	1	0	0	0	0	0	0	0	0	0	0	0	0	0	0	0
	GH127	0	0	0	0	0	0	0	0	0	0	0	0	0	0	1	0	0	0	0	0	0	0	0	0	0
	GH128	0	0	0	0	0	0	0	0	0	0	1	0	0	0	0	0	0	0	0	0	0	0	1	0	0
	GH130	1	1	1	1	1	0	1	1	1	1	1	1	1	1	1	0	1	1	0	1	0	1	1	1	0
	GH131	0	0	0	0	0	0	0	1	0	0	0	0	0	0	1	0	0	0	0	0	0	1	0	0	0
	GH133	1	1	1	1	1	0	1	1	1	1	1	0	1	1	1	1	1	1	1	1	1	1	1	1	1
	GH135	0	0	0	1	0	1	0	0	1	0	1	1	0	0	0	1	0	0	0	0	0	0	0	0	1
	GH136	0	0	0	0	1	0	0	0	0	0	0	0	0	0	1	0	0	0	0	0	1	0	0	0	0
	GH140	0	0	0	0	0	0	0	0	0	1	0	0	0	0	0	0	0	0	0	0	0	0	0	0	0
	GH141	0	0	0	0	0	0	0	0	0	1	0	0	0	0	0	0	0	0	0	0	0	0	0	0	0
	GH144	0	0	0	0	0	0	0	1	0	1	0	0	1	0	1	0	0	0	0	0	0	1	0	0	1
	GH145	0	0	1	0	0	0	0	0	0	1	0	0	0	0	0	0	0	0	0	0	0	0	0	0	0
	GH146	0	0	0	0	0	0	0	0	0	1	0	0	0	0	1	0	0	0	1	0	0	0	0	0	0
	GH147	0	0	0	0	0	0	0	0	0	0	0	0	0	0	1	0	0	0	0	0	0	0	0	0	0
	GH148	0	0	0	0	0	0	0	0	0	1	0	0	0	0	0	0	0	0	0	0	0	0	0	0	0
	GH149	0	0	0	0	0	0	0	0	1	0	0	0	0	0	1	0	0	0	0	0	0	0	0	0	0
	GH151	0	0	0	0	0	0	0	0	0	1	0	0	0	0	0	0	0	0	0	0	0	0	0	0	0
	GH152	1	0	0	0	0	0	0	0	0	0	1	0	0	0	0	0	0	0	0	0	0	0	1	0	0
	GH154	1	0	1	0	0	0	0	0	0	1	0	0	0	1	0	0	0	0	0	0	1	0	1	0	0
	GH158	0	0	0	0	0	0	0	0	0	1	0	0	0	0	1	0	0	0	0	0	0	0	0	0	0
	GH161	0	0	0	0	0	0	0	0	0	0	0	0	1	0	0	0	0	0	0	0	0	0	0	0	0
	GH163	0	0	0	0	0	0	0	0	0	0	0	0	0	0	1	0	0	0	0	0	0	0	0	0	0
	GH165	0	0	0	0	0	0	0	0	0	1	0	0	0	0	0	0	0	0	0	0	0	0	0	0	0
Glycosyl Transferases	GT0	1	1	1	1	1	1	1	1	0	1	1	1	1	1	1	1	1	1	1	1	1	1	1	1	1
	GT1	1	1	1	1	1	1	1	1	1	1	1	1	1	1	1	1	1	1	1	1	1	1	1	1	1
	GT2	1	1	1	1	1	1	1	1	1	1	1	1	1	1	1	1	1	1	1	1	1	1	1	1	1
	GT3	0	0	0	0	0	0	0	1	0	0	0	0	0	0	1	0	0	0	0	0	0	1	0	0	0
	GT4	1	1	1	1	1	1	1	1	1	1	1	1	1	1	1	1	1	1	1	1	1	1	1	1	1
	GT5	1	1	1	1	1	1	1	1	1	1	1	1	1	1	1	1	1	1	1	1	1	1	1	1	1
	GT7	1	1	1	1	1	1	1	1	1	1	1	1	1	1	1	1	1	1	1	1	1	1	1	1	1
	GT8	1	1	1	1	1	1	1	1	1	1	1	1	0	1	1	1	1	1	1	1	1	1	1	1	1

GT9	1	1	1	1	0	1	1	1	1	1	1	1	1	1	1	1	1	1	1	0	1	1	1	1	1
GT10	0	0	1	0	1	0	0	1	1	1	1	0	0	1	1	1	1	1	1	0	0	1	0	1	0
GT11	0	1	1	0	0	1	0	0	1	0	1	1	0	1	0	0	0	0	0	0	0	0	0	0	0
GT13	0	1	1	1	1	1	1	1	1	1	1	1	1	1	1	1	1	1	1	1	1	1	1	1	1
GT14	0	0	0	0	0	0	1	0	0	0	0	1	0	1	0	0	0	0	1	0	0	0	0	0	0
GT17	0	0	0	0	0	0	0	0	0	0	0	0	0	0	1	0	0	0	0	0	0	1	0	0	0
GT19	1	1	1	0	1	1	1	1	1	1	1	1	0	1	1	1	1	0	0	1	1	1	1	0	1
GT20	1	1	1	0	1	1	1	0	1	1	0	1	0	1	1	1	0	1	0	1	1	0	1	0	1
GT21	0	0	0	0	0	0	1	0	0	0	0	1	1	0	0	0	0	0	0	0	0	0	0	0	1
GT22	0	1	1	1	1	1	1	1	0	1	1	1	1	1	1	0	1	1	0	1	1	1	1	1	1
GT23	0	0	0	0	0	0	0	0	0	0	1	0	0	0	0	0	0	0	0	0	0	0	0	0	0
GT24	0	1	0	0	0	0	0	1	0	0	0	0	1	0	0	0	0	0	0	0	0	0	0	0	0
GT25	0	0	0	0	0	0	0	0	0	0	0	1	0	1	0	1	0	0	0	0	0	0	0	0	1
GT26	1	1	0	1	1	1	1	1	1	1	1	1	0	1	0	1	1	0	1	1	0	0	1	0	1
GT27	1	1	1	1	1	1	1	0	1	1	1	1	0	1	0	1	1	0	0	1	1	1	1	0	1
GT28	1	1	1	1	1	1	1	1	1	1	1	1	1	1	1	1	1	1	1	1	1	1	1	1	1
GT29	1	1	1	1	1	1	1	1	0	1	1	1	1	1	1	1	1	1	1	1	1	1	1	1	1
GT30	0	1	1	1	1	1	1	1	1	1	1	1	1	1	1	1	1	0	0	0	1	1	1	0	1
GT32	1	0	1	0	0	1	1	1	0	1	1	1	1	1	1	1	1	0	1	1	1	1	1	1	0
GT34	0	0	0	0	0	0	0	0	0	0	0	0	1	0	0	0	0	0	0	1	0	0	0	0	0
GT35	1	1	1	1	1	1	0	1	1	1	1	0	1	1	1	1	1	1	1	0	0	1	1	0	0
GT41	1	1	1	1	0	0	1	0	1	1	1	0	0	1	1	1	1	1	1	0	1	0	0	0	0
GT45	0	0	0	0	0	0	0	0	0	0	0	0	0	0	0	1	0	0	0	0	0	0	0	0	0
GT47	0	1	1	1	1	1	1	1	1	1	1	1	1	1	1	1	1	1	1	1	1	1	1	1	1
GT49	0	0	0	1	0	0	1	1	1	1	0	0	0	0	1	1	0	0	0	0	0	1	0	0	1
GT51	1	1	1	1	1	1	1	1	1	1	1	1	1	1	1	1	1	1	1	1	1	1	1	1	1
GT57	0	0	0	1	1	0	0	0	0	0	0	0	0	0	1	0	0	0	0	0	1	0	0	0	0
GT60	0	0	0	0	0	0	0	0	0	0	1	0	0	0	0	0	0	0	0	0	0	0	0	0	0
GT61	1	1	1	1	1	1	1	0	1	0	1	1	1	1	0	1	1	1	1	1	1	0	1	1	1
GT64	0	0	0	0	0	0	0	0	0	0	0	0	0	0	0	0	0	0	0	0	0	1	1	0	0
GT66	1	1	1	1	0	1	1	1	1	1	1	1	0	1	1	1	1	1	1	1	1	1	1	1	1
GT68	1	1	1	1	1	1	1	1	1	1	1	1	1	1	1	1	1	1	1	1	1	1	1	1	1
GT74	0	0	0	0	1	0	0	0	0	0	0	0	0	0	0	0	0	0	0	0	0	0	0	0	0
GT80	1	1	1	1	0	0	0	0	1	0	0	0	0	1	0	1	0	0	0	0	0	0	0	0	1
GT81	1	1	0	1	1	0	1	1	0	1	0	1	0	0	1	0	1	1	0	0	0	1	1	0	0
GT82	0	0	0	0	0	0	0	0	0	0	1	0	0	0	0	0	0	0	0	0	0	0	0	0	0
GT83	1	1	1	0	0	1	1	1	1	1	1	1	1	1	1	1	1	0	1	1	1	1	1	1	1
GT84	0	1	1	1	0	0	1	0	1	0	0	1	1	1	0	0	1	0	0	1	0	0	0	0	1
GT87	1	0	0	0	0	0	0	0	0	0	0	0	0	1	0	1	0	0	0	0	0	1	1	0	0
GT89	1	0	0	0	0	0	0	0	0	0	0	0	0	0	0	0	0	0	0	0	0	0	0	0	0
GT90	1	0	1	0	0	0	1	1	0	1	0	0	0	1	1	1	1	0	0	0	0	1	1	0	1
GT93	0	0	0	0	0	0	0	0	0	0	0	0	0	0	0	1	0	0	0	0	0	0	0	0	0
GT95	0	1	1	1	0	1	0	1	0	1	0	0	0	1	1	1	1	1	0	0	1	1	0	0	0
GT96	0	0	0	0	0	1	0	0	0	0	1	0	0	0	0	0	0	0	0	0	0	0	0	0	0
GT101	0	0	0	0	0	0	1	0	0	0	0	0	0	0	0	0	0	0	0	0	0	0	0	0	0
GT102	1	0	0	0	0	0	0	0	0	0	0	0	0	0	0	0	0	0	0	0	0	0	0	0	0

	GT103	1	0	0	0	0	0	0	0	0	0	0	0	0	0	0	0	0	0	0	0	0	0	0	0	0
	GT104	1	1	0	1	0	1	0	0	0	0	1	0	0	0	0	0	0	0	0	0	0	0	0	0	0
	GT107	0	0	1	0	0	0	1	0	0	0	0	1	0	0	0	0	0	0	0	0	0	0	0	0	0
Polysaccharide Lyases	PL0	1	1	0	1	0	0	1	1	0	1	1	1	1	1	1	1	0	0	0	0	1	0	0	0	1
	PL1	0	0	0	0	0	0	0	1	0	1	1	0	0	0	1	0	0	0	0	0	0	1	0	0	0
	PL6	0	0	0	0	0	0	0	0	0	0	0	0	0	0	1	0	0	0	0	0	0	0	0	0	0
	PL8	0	0	0	0	1	0	0	1	0	1	0	0	0	0	1	0	0	0	0	0	0	1	0	0	0
	PL11	0	0	0	0	0	0	0	0	0	1	0	0	0	0	0	0	0	0	0	0	0	0	0	0	0
	PL12	1	0	0	1	0	0	0	1	0	1	0	0	1	0	0	0	1	1	1	1	1	0	0	0	0
	PL14	0	0	1	0	0	0	0	0	0	0	0	0	0	0	0	0	1	0	0	0	1	0	0	0	0
	PL28	0	0	0	0	0	0	0	0	0	0	0	0	0	0	1	0	0	0	0	0	0	1	0	0	0
	PL31	0	0	0	0	0	0	1	0	0	0	0	0	0	0	0	0	0	0	0	0	0	0	0	0	0
	PL35	0	0	0	0	0	0	0	0	0	1	0	0	0	0	0	0	0	0	0	0	0	0	0	0	0
