# Supplementary figures and images for: Investigating eukaryotic and prokaryotic diversity and functional potential in the cold and alkaline ikaite columns in Greenland
Source: Front Microbiol. 2024 Apr 9;15:1358787. doi: 10.3389/fmicb.2024.1358787 (PMC11035741; doi:10.3389/fmicb.2024.1358787)

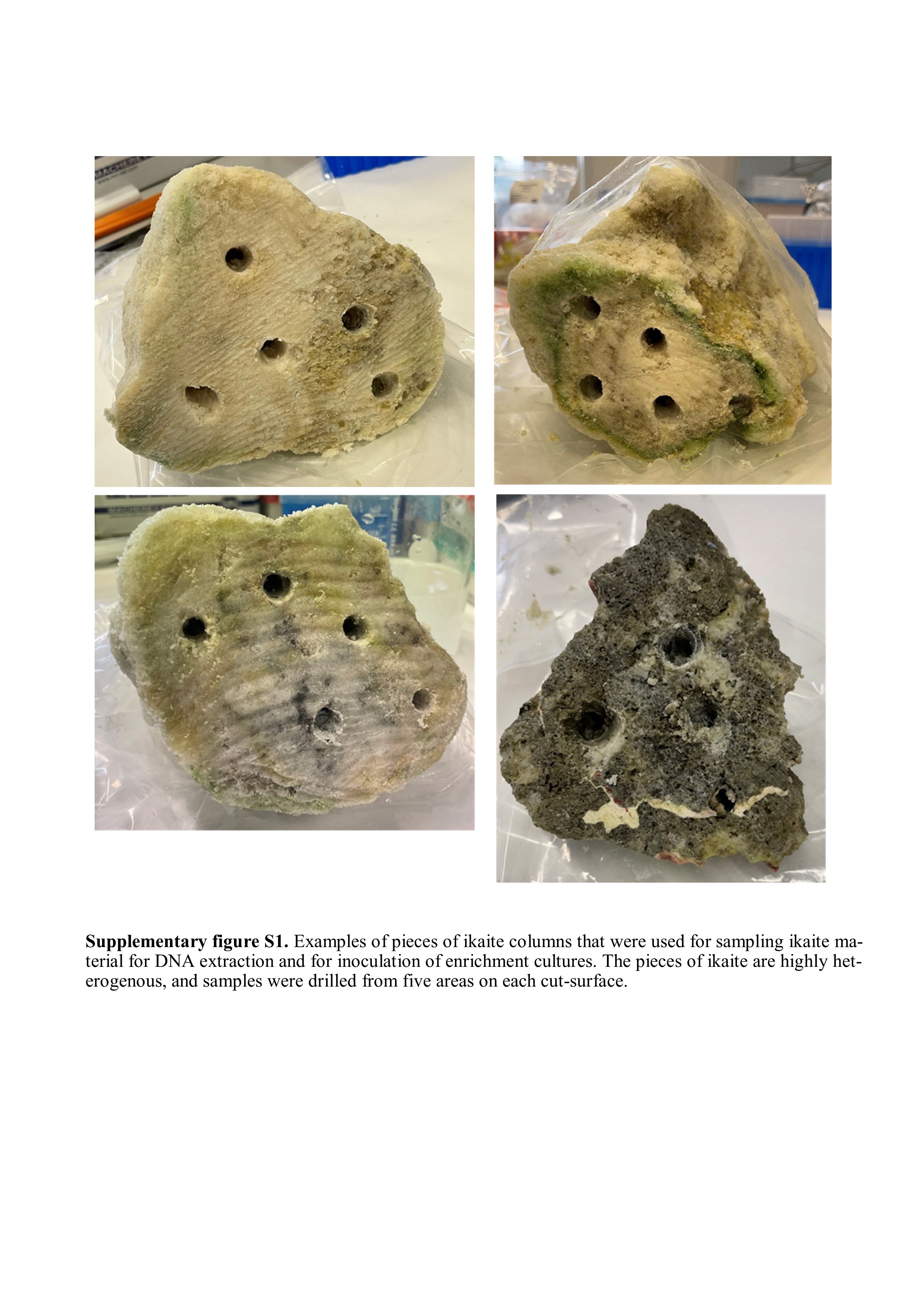

Supplement: Supplementary file 1 [file Data_Sheet_1.zip › Image 1.JPEG]

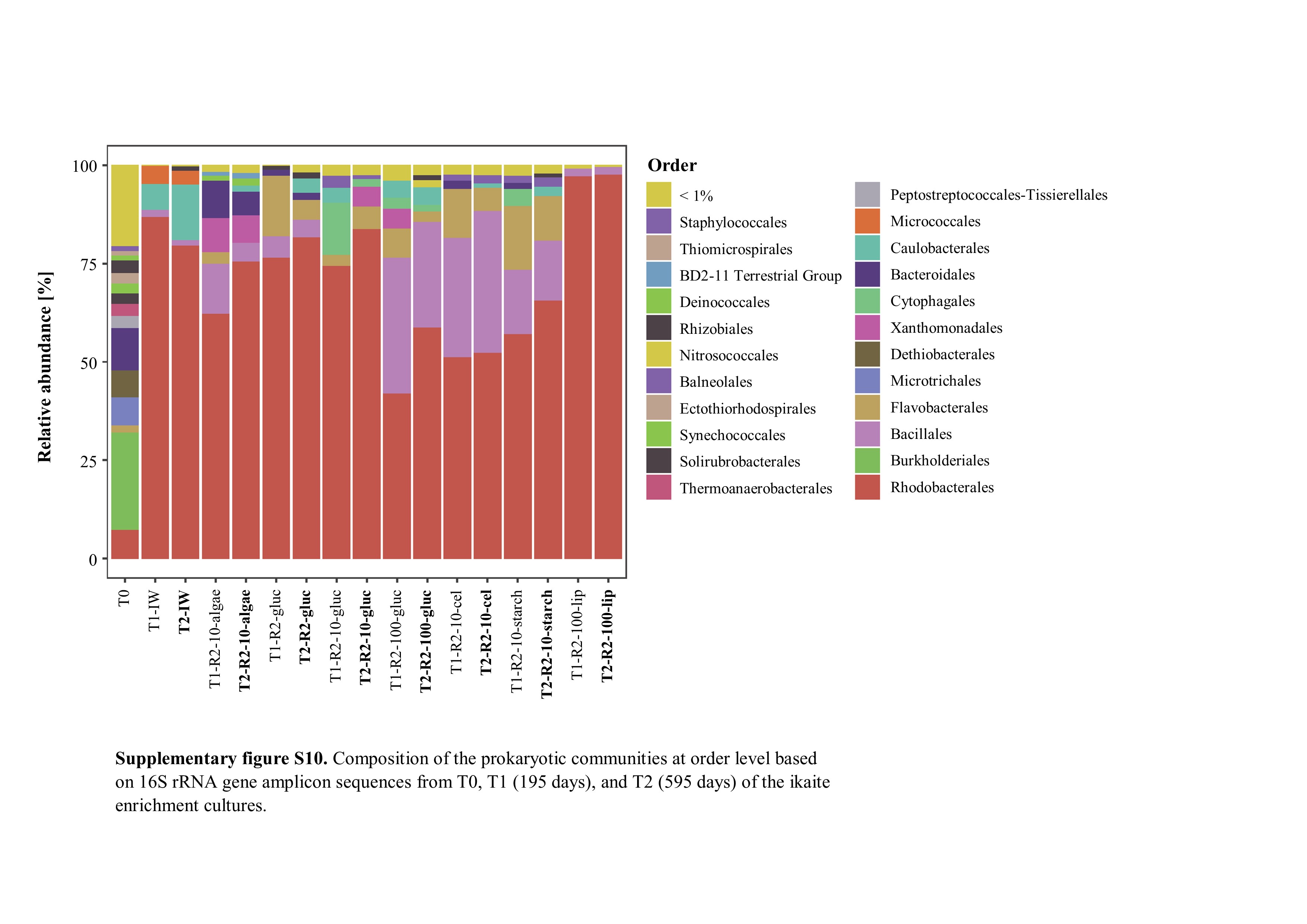

Supplement: Supplementary file 1 [file Data_Sheet_1.zip › Image 10.JPEG]

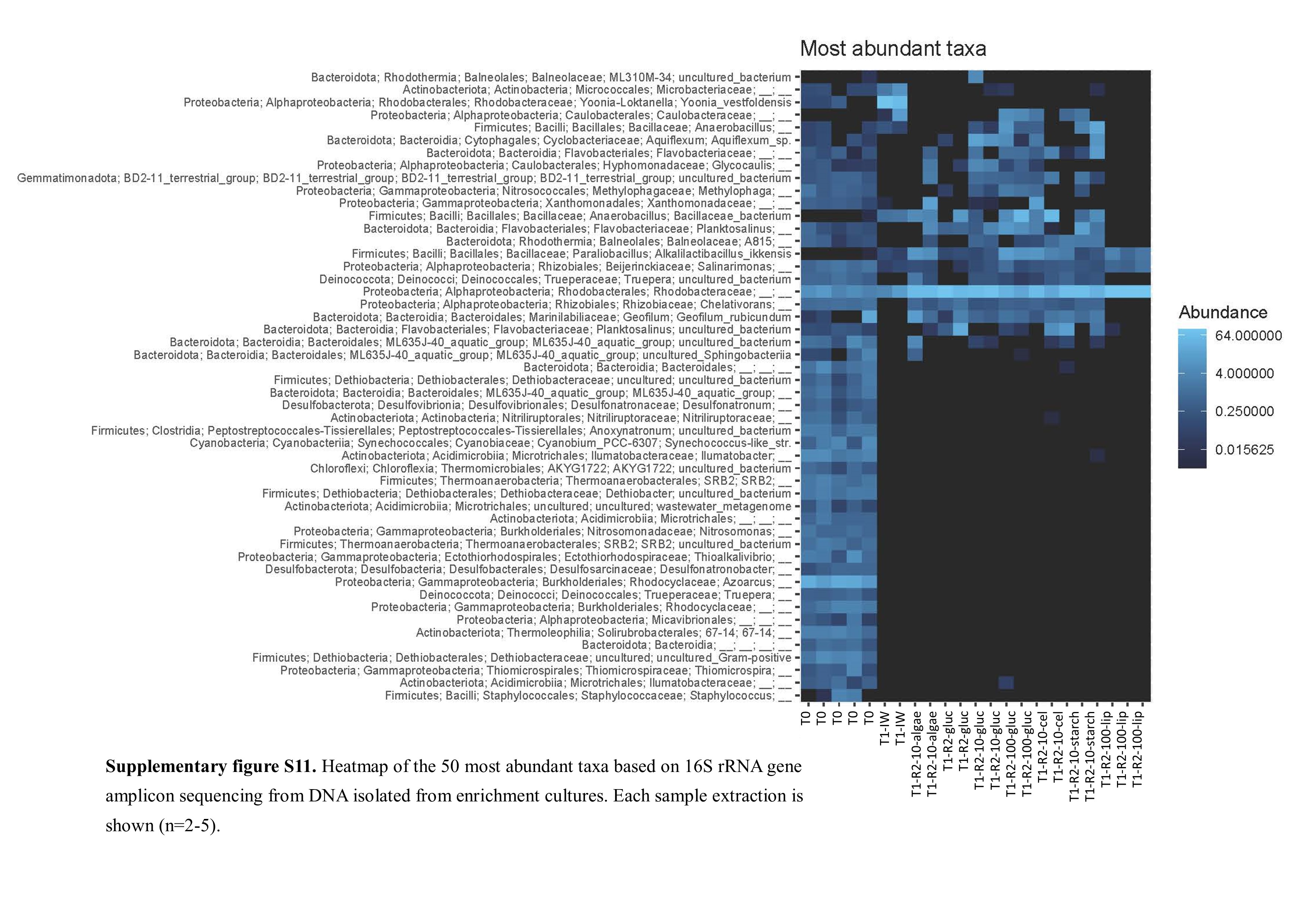

Supplement: Supplementary file 1 [file Data_Sheet_1.zip › Image 11.JPEG]

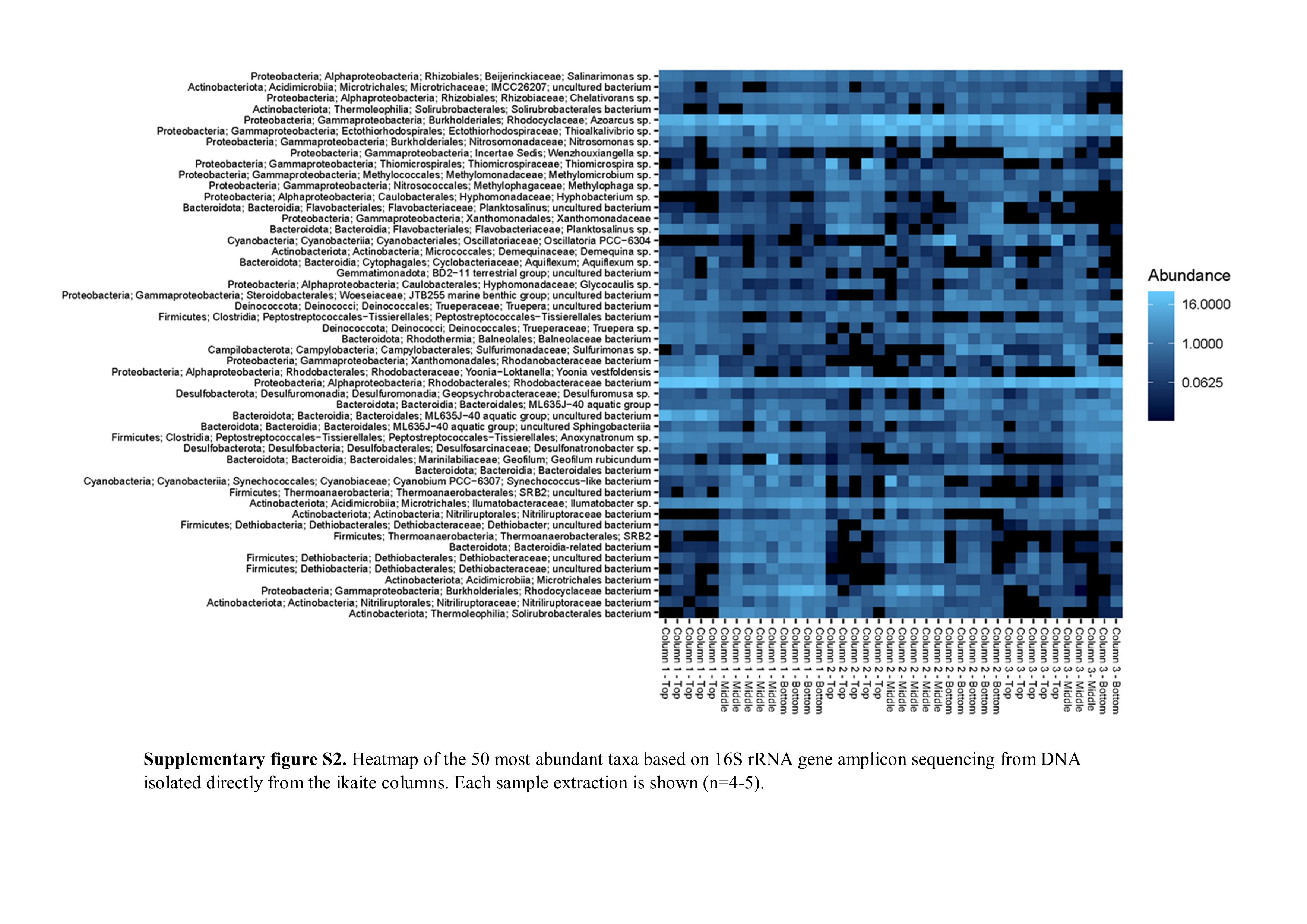

Supplement: Supplementary file 1 [file Data_Sheet_1.zip › Image 2.JPEG]

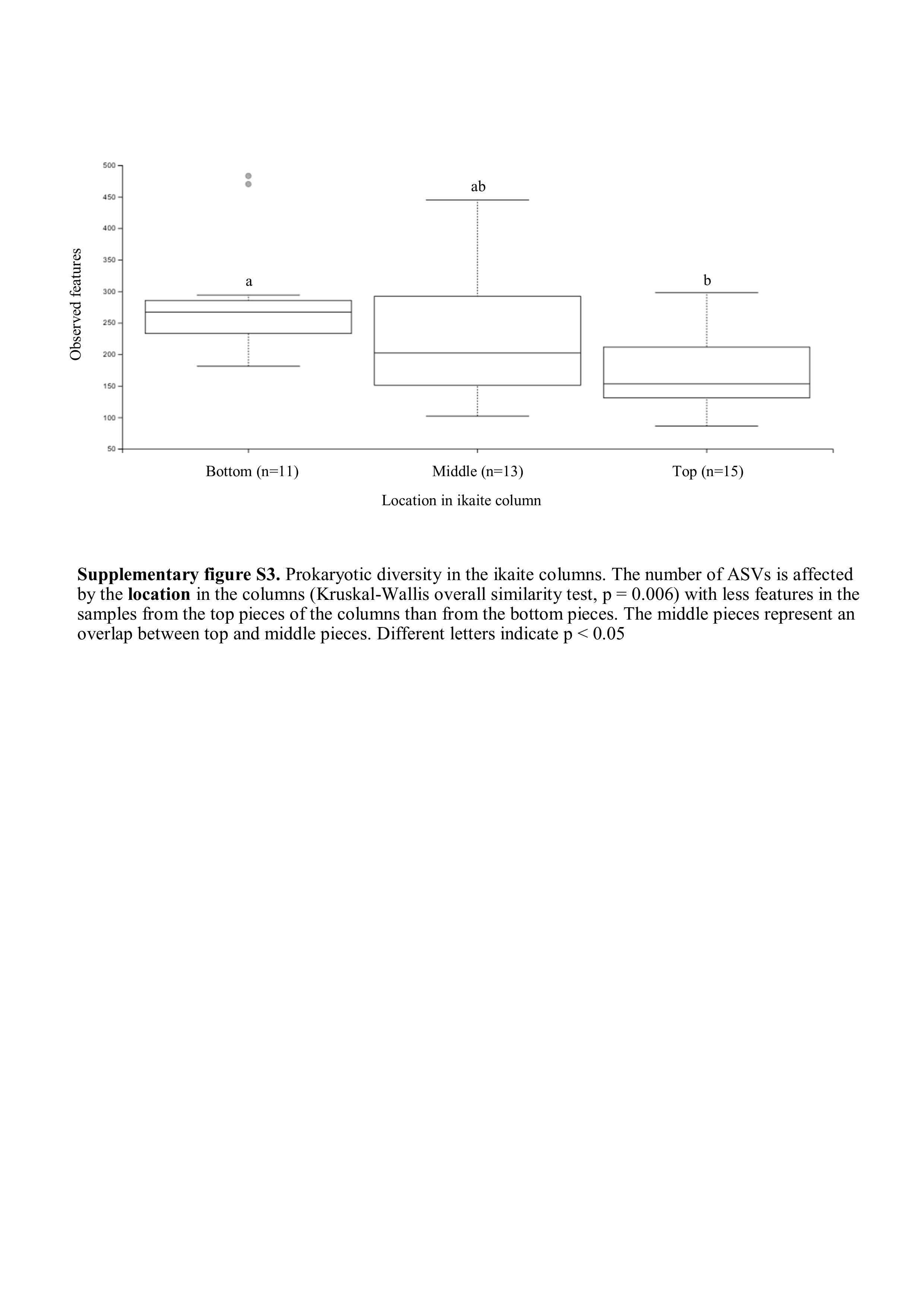

Supplement: Supplementary file 1 [file Data_Sheet_1.zip › Image 3.JPEG]

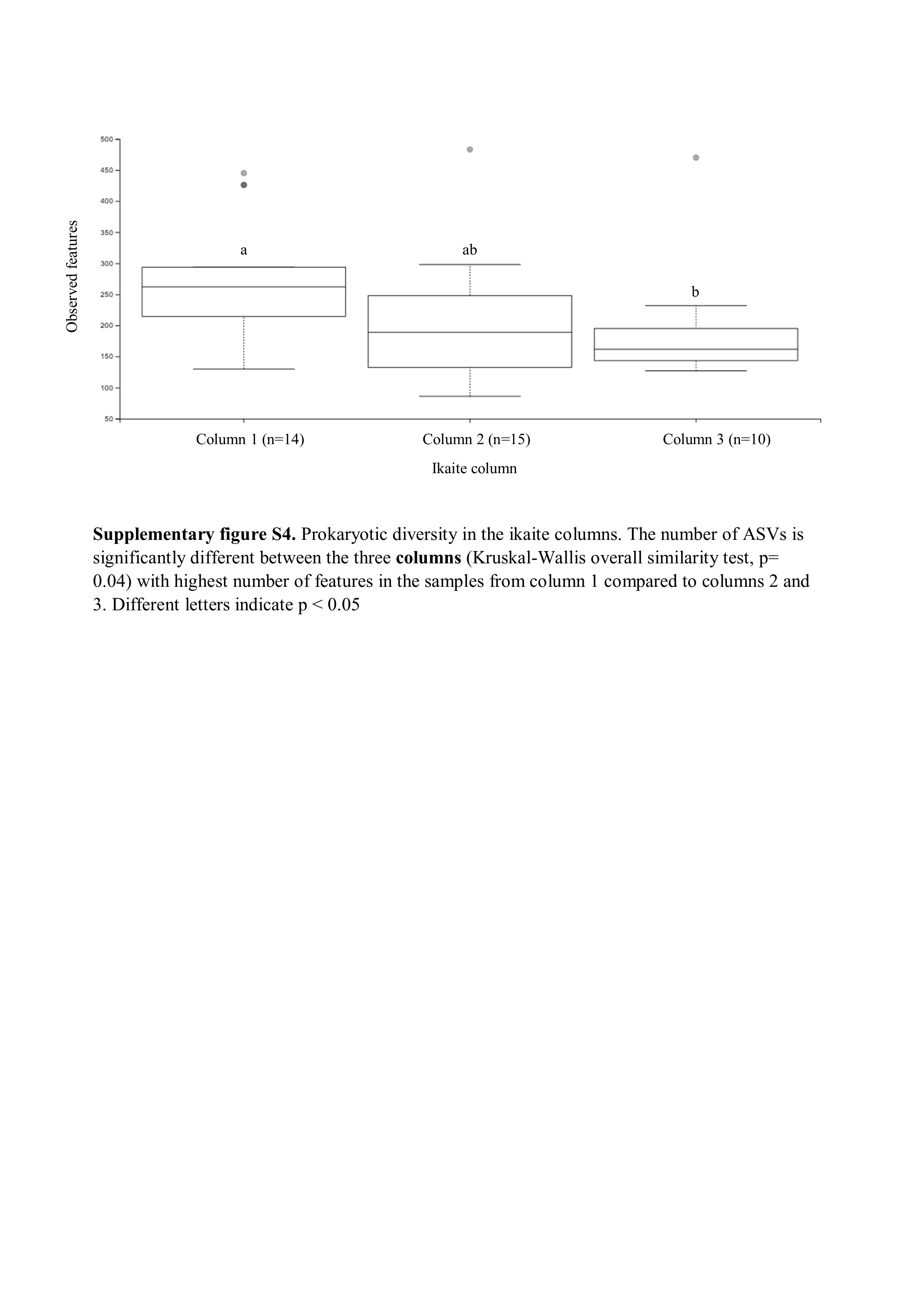

Supplement: Supplementary file 1 [file Data_Sheet_1.zip › Image 4.JPEG]

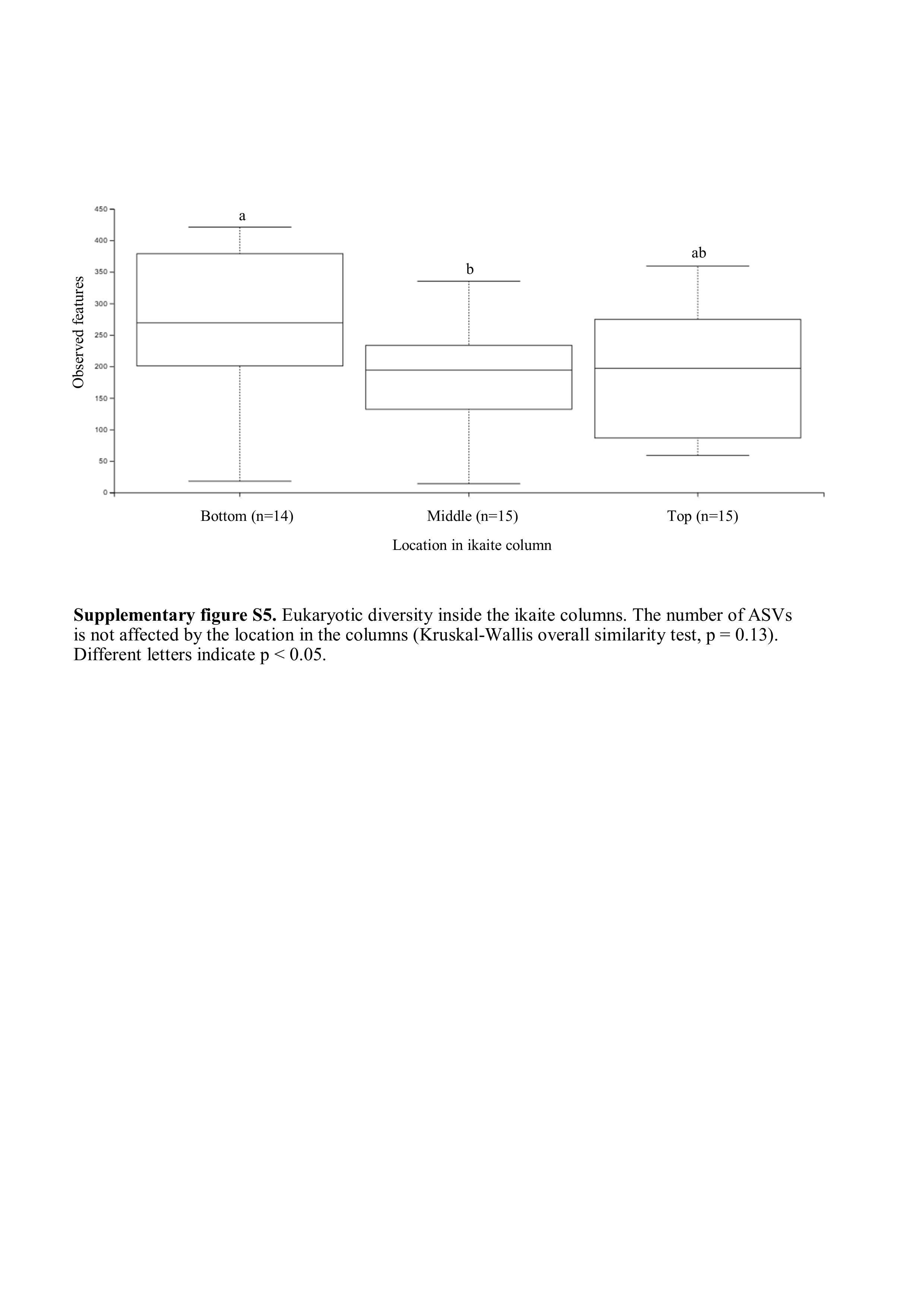

Supplement: Supplementary file 1 [file Data_Sheet_1.zip › Image 5.JPEG]

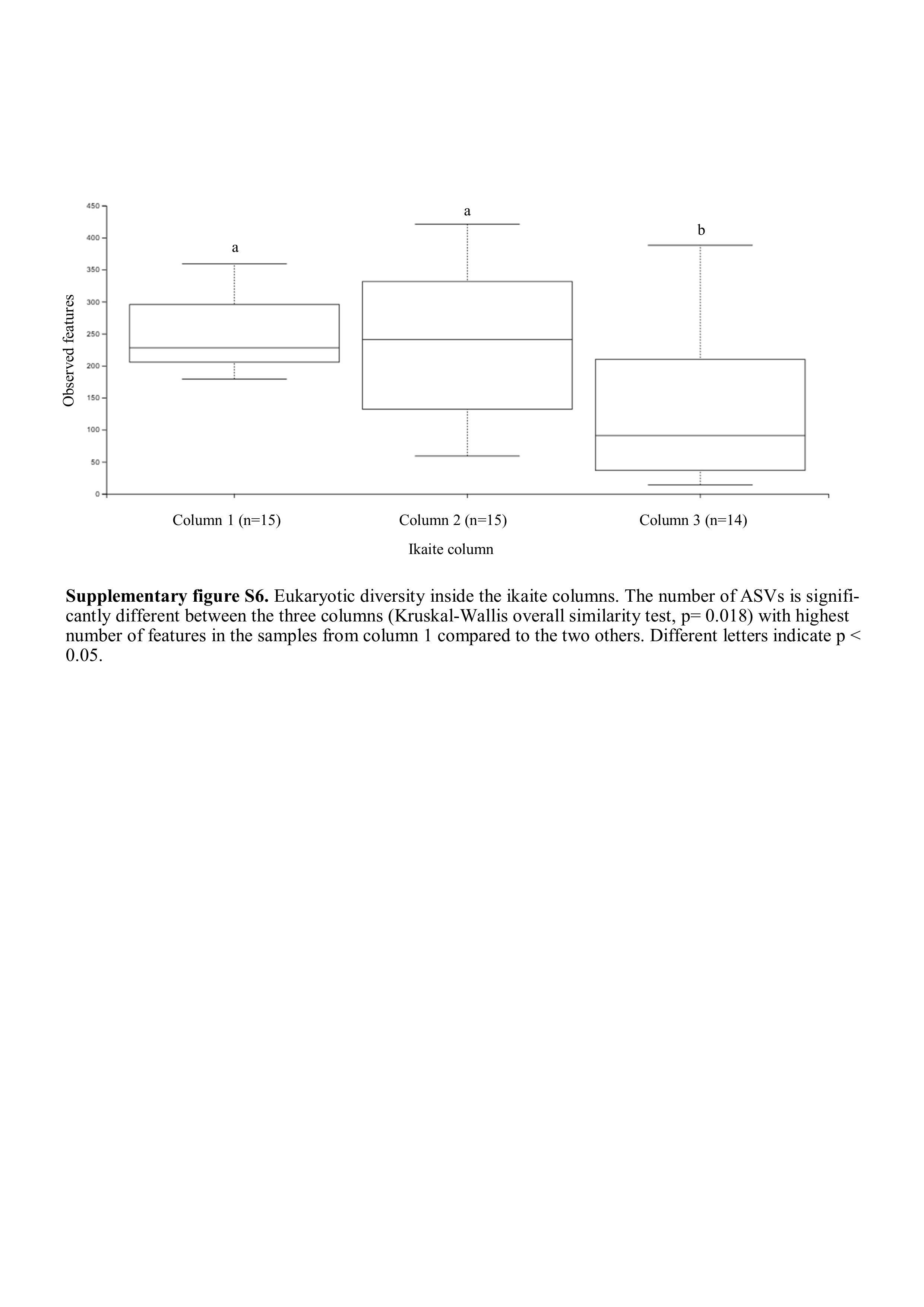

Supplement: Supplementary file 1 [file Data_Sheet_1.zip › Image 6.JPEG]

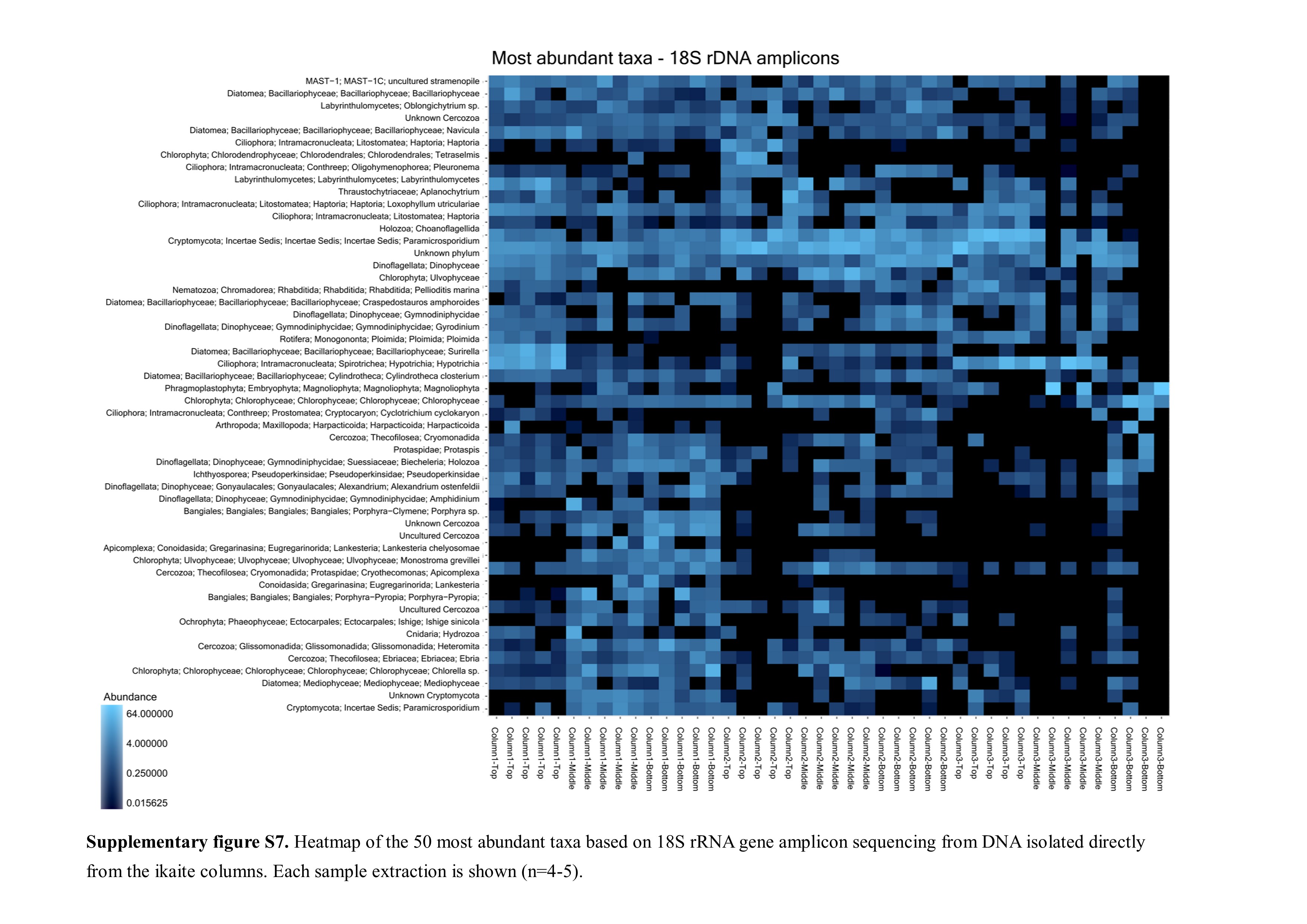

Supplement: Supplementary file 1 [file Data_Sheet_1.zip › Image 7.JPEG]

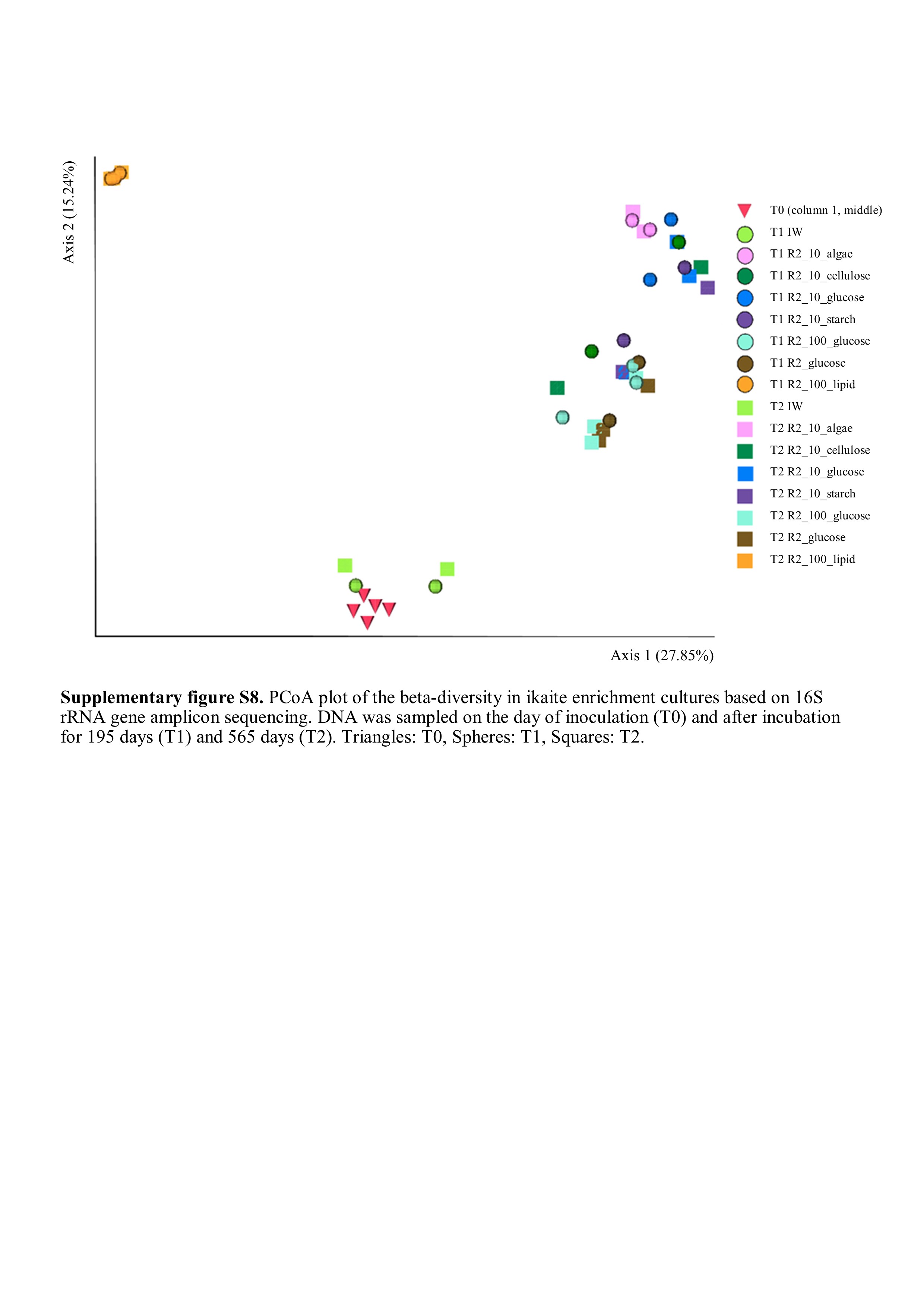

Supplement: Supplementary file 1 [file Data_Sheet_1.zip › Image 8.JPEG]

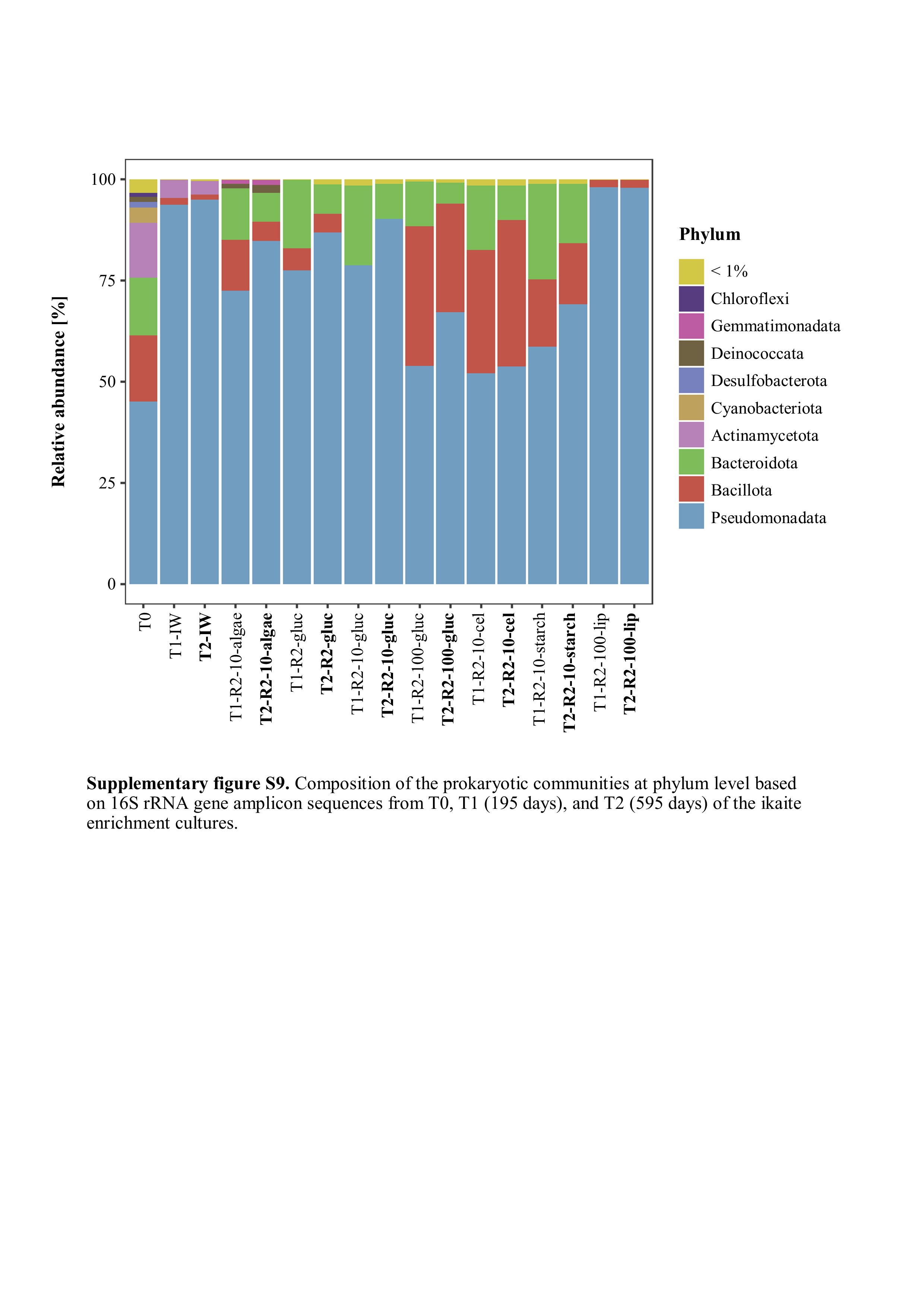

Supplement: Supplementary file 1 [file Data_Sheet_1.zip › Image 9.JPEG]
